# Supplementary material for: Global burden and regional disparities of rheumatoid arthritis among the working-age population: A comprehensive analysis from 1990 to 2021 with projections to 2040
Source: PLoS One. 2025 Jun 4;20(6):e0325127. doi: 10.1371/journal.pone.0325127 (PMC12136291; doi:10.1371/journal.pone.0325127)
Supplement: S3 Fig — (DOCX) [file pone.0325127.s003.docx]

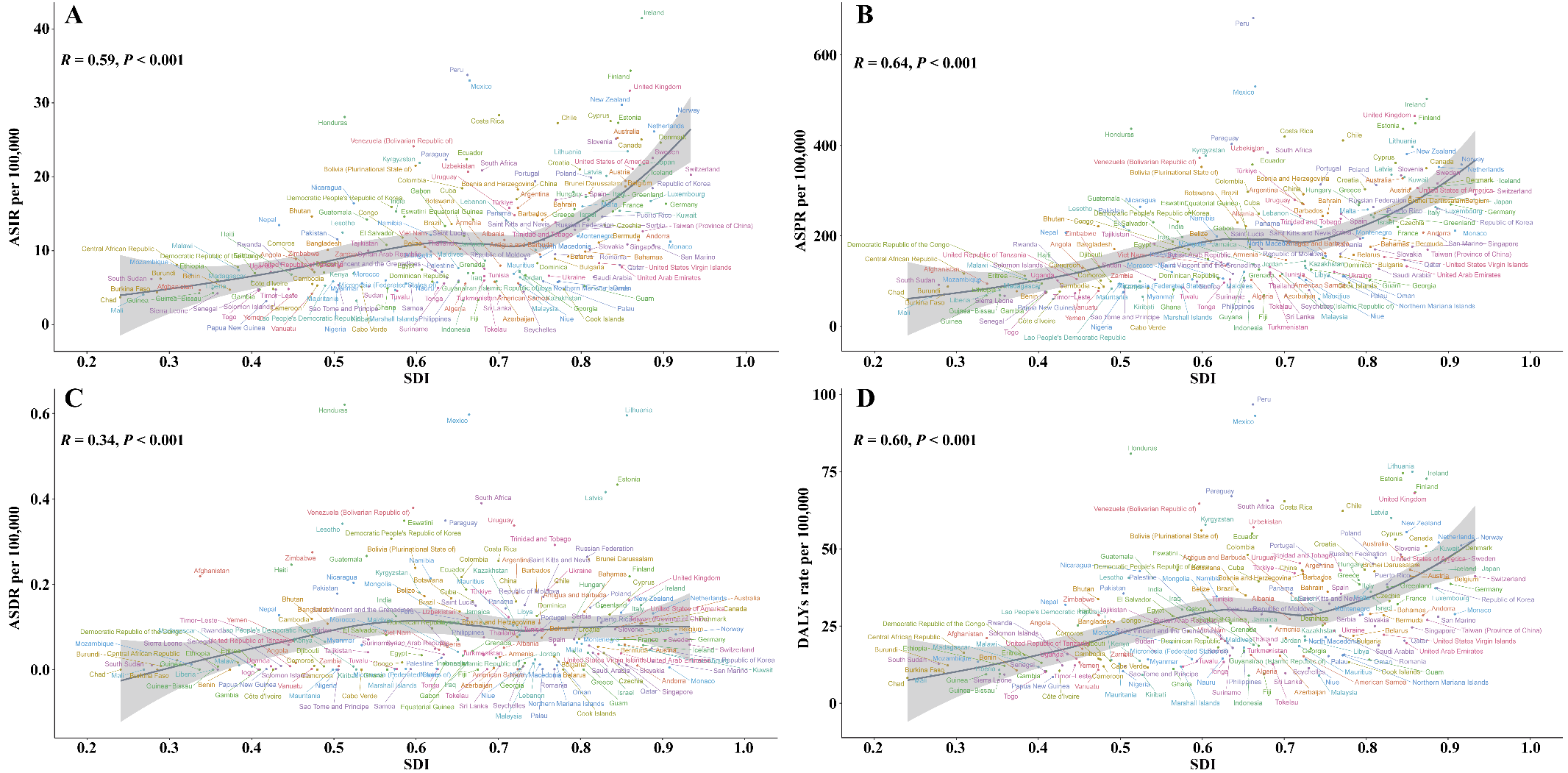


**S3 Fig.** Associations between SDI and ASIR (A), ASPR (B), ASDR (C) and age-standardized DALYs (D) rate of rheumatoid arthritis among the working-age population across 204 countries in 2021.
