# Supplementary material for: Global burden and regional disparities of rheumatoid arthritis among the working-age population: A comprehensive analysis from 1990 to 2021 with projections to 2040
Source: PLoS One. 2025 Jun 4;20(6):e0325127. doi: 10.1371/journal.pone.0325127 (PMC12136291; doi:10.1371/journal.pone.0325127)
Supplement: S4 Fig — (DOCX) [file pone.0325127.s004.docx]

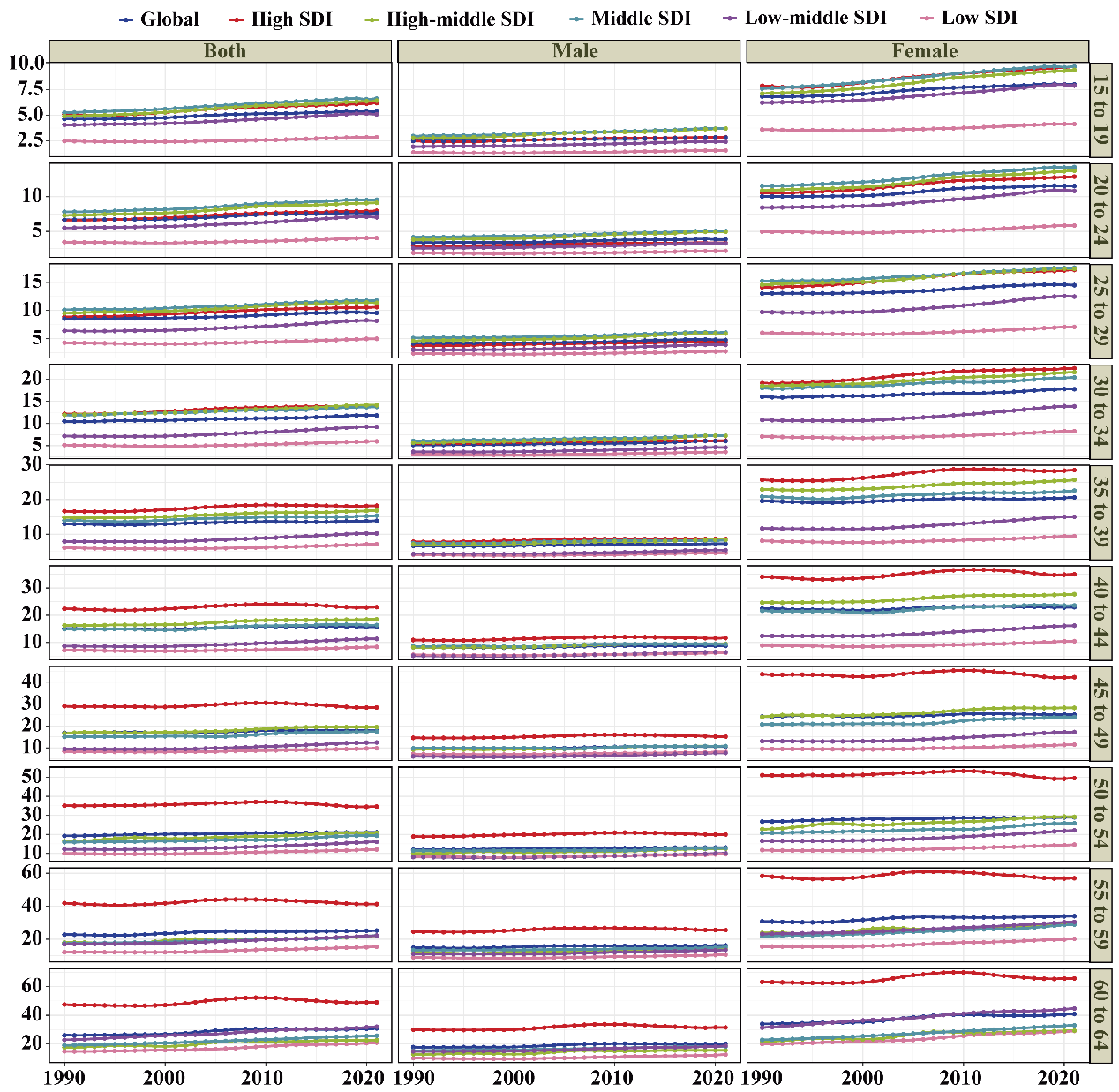


**S4 Fig.** The temporal trends of rheumatoid arthritis incidence rate among the working-age population across different age groups, globally and in the SDI regions from 1990 to 2021.
