# Supplementary material for: Global burden and regional disparities of rheumatoid arthritis among the working-age population: A comprehensive analysis from 1990 to 2021 with projections to 2040
Source: PLoS One. 2025 Jun 4;20(6):e0325127. doi: 10.1371/journal.pone.0325127 (PMC12136291; doi:10.1371/journal.pone.0325127)
Supplement: S1 Table — (DOCX) [file pone.0325127.s016.docx]

**S1 Table.** National trends in the burden of rheumatoid arthritis incidence among working-age population: 1990−2021

| **Location** | **1990** | | **2021** | | **EAPC (95% CI)** |
| --- | --- | --- | --- | --- | --- |
|  | **Number** | **ASR** | **Number** | **ASR** |  |
| Afghanistan | 185.21 (112.56, 277.45) | 3.84 (2.32, 5.73) | 765.57 (479.15, 1107.49) | 4.67 (2.88, 6.78) | 0.73 (0.55, 0.92) |
| Albania | 132.53 (77.87, 200.04) | 6.95 (4.08, 10.49) | 219.69 (130.97, 330.01) | 11.47 (6.81, 17.17) | 1.87 (1.79, 1.95) |
| Algeria | 459.06 (277.22, 696.00) | 3.52 (2.09, 5.37) | 1578.74 (953.92, 2315.60) | 5.53 (3.36, 8.12) | 1.59 (1.48, 1.69) |
| American Samoa | 1.26 (0.71, 1.95) | 4.49 (2.51, 7.00) | 1.77 (1.02, 2.73) | 5.59 (3.22, 8.60) | 0.60 (0.55, 0.66) |
| Andorra | 4.05 (2.42, 6.10) | 10.21 (6.11, 15.38) | 7.80 (4.71, 11.56) | 11.39 (6.85, 16.96) | 0.44 (0.41, 0.47) |
| Angola | 304.19 (185.65, 452.06) | 6.84 (4.25, 10.03) | 1235.33 (763.09, 1805.80) | 8.87 (5.58, 12.82) | 0.96 (0.85, 1.06) |
| Antigua and Barbuda | 2.34 (1.36, 3.56) | 6.64 (3.87, 10.13) | 6.28 (3.67, 9.61) | 9.49 (5.56, 14.55) | 1.09 (0.99, 1.18) |
| Argentina | 2044.37 (1225.86, 3016.00) | 10.26 (6.16, 15.12) | 4827.60 (2943.49, 6999.90) | 15.77 (9.61, 22.92) | 1.24 (1.13, 1.36) |
| Armenia | 113.99 (66.19, 174.14) | 5.23 (3.03, 7.99) | 165.56 (97.27, 247.60) | 7.98 (4.70, 11.93) | 1.51 (1.45, 1.56) |
| Australia | 2546.64 (1602.90, 3718.55) | 22.68 (14.30, 33.13) | 4930.97 (3006.18, 7219.39) | 25.26 (15.22, 37.26) | 0.45 (0.26, 0.64) |
| Austria | 1049.40 (643.53, 1526.87) | 18.92 (11.53, 27.63) | 1361.00 (835.29, 1977.75) | 19.45 (11.78, 28.46) | 0.17 (0.01, 0.33) |
| Azerbaijan | 204.56 (120.13, 310.29) | 4.55 (2.65, 6.92) | 437.51 (261.20, 667.50) | 5.80 (3.47, 8.83) | 1.08 (0.78, 1.39) |
| Bahamas | 11.93 (6.95, 18.02) | 7.60 (4.42, 11.48) | 27.48 (16.46, 41.70) | 9.88 (5.93, 14.98) | 0.91 (0.79, 1.02) |
| Bahrain | 27.56 (15.43, 42.95) | 8.30 (4.63, 12.96) | 172.82 (96.14, 264.80) | 14.18 (7.91, 21.89) | 1.64 (1.57, 1.72) |
| Bangladesh | 3404.40 (2096.85, 4962.85) | 6.44 (3.95, 9.42) | 10676.25 (6687.53, 15617.73) | 10.12 (6.33, 14.83) | 1.67 (1.47, 1.88) |
| Barbados | 16.24 (9.39, 24.86) | 10.36 (5.97, 15.87) | 32.30 (18.60, 49.33) | 14.76 (8.53, 22.50) | 1.03 (0.93, 1.14) |
| Belarus | 415.56 (241.18, 629.17) | 5.90 (3.43, 8.91) | 628.49 (375.84, 935.96) | 9.22 (5.51, 13.74) | 1.51 (1.37, 1.66) |
| Belgium | 1224.19 (749.72, 1809.82) | 16.79 (10.19, 24.94) | 1606.61 (980.25, 2361.66) | 18.70 (11.22, 27.75) | 0.40 (0.35, 0.45) |
| Belize | 6.59 (3.91, 9.96) | 7.35 (4.34, 11.11) | 29.07 (17.32, 43.08) | 10.50 (6.24, 15.56) | 1.21 (1.15, 1.26) |
| Benin | 76.05 (42.35, 119.78) | 3.79 (2.12, 5.94) | 295.29 (167.85, 455.77) | 4.72 (2.69, 7.23) | 0.76 (0.67, 0.86) |
| Bermuda | 3.48 (2.01, 5.42) | 8.07 (4.66, 12.57) | 4.99 (2.84, 7.88) | 11.09 (6.38, 17.34) | 1.13 (1.04, 1.23) |
| Bhutan | 27.92 (16.39, 42.40) | 9.35 (5.50, 14.21) | 71.63 (42.74, 108.11) | 14.64 (8.76, 22.08) | 1.59 (1.46, 1.73) |
| Bolivia (Plurinational State of) | 420.22 (253.71, 621.71) | 13.03 (7.85, 19.32) | 1575.19 (958.86, 2300.00) | 21.51 (13.09, 31.39) | 1.69 (1.66, 1.72) |
| Bosnia and Herzegovina | 380.58 (232.03, 556.80) | 12.22 (7.44, 17.86) | 455.82 (287.44, 658.01) | 18.28 (11.52, 26.28) | 1.61 (1.49, 1.72) |
| Botswana | 62.56 (38.21, 91.44) | 10.19 (6.22, 14.82) | 216.65 (133.10, 315.02) | 13.61 (8.35, 19.84) | 1.03 (0.86, 1.20) |
| Brazil | 9854.35 (6033.33, 14677.68) | 10.58 (6.38, 15.92) | 15156.40 (9301.89, 22566.88) | 10.24 (6.33, 15.19) | 0.13 (0.05, 0.21) |
| Brunei Darussalam | 17.76 (10.73, 26.59) | 13.87 (8.65, 20.36) | 58.28 (36.23, 84.63) | 17.82 (11.16, 25.85) | 0.81 (0.69, 0.94) |
| Bulgaria | 419.27 (250.80, 626.16) | 7.02 (4.19, 10.50) | 423.48 (256.81, 622.41) | 8.90 (5.38, 13.14) | 0.79 (0.64, 0.94) |
| Burkina Faso | 144.84 (80.40, 226.28) | 3.62 (2.01, 5.63) | 454.85 (257.07, 700.55) | 4.38 (2.48, 6.72) | 0.69 (0.61, 0.78) |
| Burundi | 127.43 (76.23, 193.77) | 5.74 (3.53, 8.58) | 353.42 (212.18, 527.81) | 6.21 (3.84, 9.10) | 0.34 (0.28, 0.40) |
| Cabo Verde | 6.01 (3.37, 9.36) | 3.87 (2.17, 6.02) | 19.09 (10.90, 29.22) | 5.10 (2.92, 7.80) | 1.01 (0.89, 1.14) |
| Cambodia | 164.98 (92.06, 254.65) | 3.54 (2.01, 5.41) | 575.65 (332.51, 875.75) | 5.50 (3.19, 8.33) | 1.46 (1.42, 1.50) |
| Cameroon | 209.01 (115.65, 328.62) | 4.40 (2.44, 6.90) | 830.88 (470.94, 1277.21) | 5.29 (3.02, 8.11) | 0.64 (0.45, 0.83) |
| Canada | 3455.62 (2886.60, 4021.21) | 18.47 (15.43, 21.50) | 7266.42 (5987.50, 8618.16) | 25.05 (20.83, 29.42) | 0.98 (0.86, 1.09) |
| Central African Republic | 92.21 (57.07, 134.10) | 7.69 (4.83, 11.03) | 213.61 (136.17, 302.55) | 8.08 (5.21, 11.35) | 0.22 (0.17, 0.27) |
| Chad | 82.90 (45.90, 129.15) | 3.29 (1.83, 5.09) | 264.72 (149.92, 408.76) | 3.70 (2.11, 5.68) | 0.45 (0.31, 0.60) |
| Chile | 1217.89 (721.92, 1832.16) | 15.50 (9.21, 23.24) | 3758.00 (2250.93, 5566.53) | 27.28 (16.30, 40.48) | 1.88 (1.80, 1.95) |
| China | 109094.58 (64787.03, 164000.80) | 14.86 (8.82, 22.38) | 182850.18 (111605.46, 269761.76) | 16.97 (10.35, 24.98) | 0.52 (0.48, 0.56) |
| Colombia | 2164.59 (1328.65, 3142.85) | 12.29 (7.60, 17.74) | 6237.58 (3852.94, 8932.39) | 18.45 (11.39, 26.40) | 1.33 (1.17, 1.48) |
| Comoros | 11.63 (6.82, 17.84) | 6.00 (3.58, 9.08) | 30.47 (17.80, 45.76) | 7.10 (4.17, 10.63) | 0.64 (0.54, 0.75) |
| Congo | 95.15 (58.03, 140.98) | 9.27 (5.77, 13.50) | 356.15 (221.09, 523.41) | 11.78 (7.35, 17.23) | 0.92 (0.82, 1.03) |
| Cook Islands | 0.38 (0.21, 0.60) | 3.38 (1.85, 5.36) | 0.55 (0.31, 0.86) | 4.79 (2.71, 7.47) | 1.16 (1.09, 1.23) |
| Costa Rica | 261.37 (159.44, 386.26) | 16.91 (10.41, 24.77) | 950.56 (578.12, 1377.03) | 28.34 (17.24, 41.06) | 1.57 (1.49, 1.66) |
| Cote d'Ivoire | 209.56 (115.95, 329.12) | 3.85 (2.14, 6.03) | 676.83 (382.44, 1042.92) | 4.82 (2.73, 7.42) | 0.78 (0.62, 0.93) |
| Croatia | 474.34 (276.55, 716.87) | 13.03 (7.56, 19.71) | 683.82 (413.00, 992.84) | 21.01 (12.60, 30.75) | 1.58 (1.54, 1.61) |
| Cuba | 714.07 (412.91, 1098.29) | 10.02 (5.78, 15.39) | 1151.22 (679.48, 1742.71) | 14.19 (8.39, 21.34) | 1.10 (1.02, 1.17) |
| Cyprus | 107.85 (65.01, 160.06) | 21.82 (13.17, 32.35) | 295.89 (176.67, 434.89) | 27.54 (16.43, 40.63) | 1.06 (0.87, 1.25) |
| Czechia | 662.08 (394.00, 990.53) | 9.52 (5.68, 14.25) | 987.42 (598.00, 1431.78) | 13.46 (8.13, 19.58) | 1.08 (1.04, 1.12) |
| Democratic People's Republic of Korea | 1589.82 (964.68, 2342.21) | 12.03 (7.28, 17.73) | 3337.55 (2075.94, 4761.93) | 15.92 (9.84, 22.91) | 0.93 (0.89, 0.98) |
| Democratic Republic of the Congo | 1054.57 (622.42, 1585.49) | 6.54 (3.93, 9.70) | 3224.87 (1968.99, 4764.43) | 7.78 (4.82, 11.38) | 0.55 (0.43, 0.68) |
| Denmark | 701.31 (468.83, 985.84) | 18.78 (12.51, 26.48) | 1135.17 (735.48, 1622.81) | 24.62 (15.70, 35.54) | 1.02 (0.96, 1.09) |
| Djibouti | 10.14 (5.88, 15.64) | 5.60 (3.31, 8.51) | 51.87 (31.06, 76.93) | 7.05 (4.26, 10.43) | 0.79 (0.66, 0.92) |
| Dominica | 2.59 (1.52, 3.94) | 6.51 (3.80, 9.89) | 3.83 (2.26, 5.84) | 8.31 (4.91, 12.61) | 0.73 (0.65, 0.80) |
| Dominican Republic | 174.68 (101.44, 269.41) | 4.40 (2.53, 6.83) | 418.68 (246.61, 644.32) | 5.81 (3.41, 8.96) | 0.94 (0.89, 1.00) |
| Ecuador | 688.40 (415.47, 1018.43) | 13.27 (7.96, 19.65) | 2536.03 (1567.60, 3643.17) | 22.38 (13.82, 32.16) | 1.61 (1.50, 1.72) |
| Egypt | 1416.03 (846.73, 2108.55) | 4.46 (2.64, 6.67) | 4836.84 (2956.83, 7136.00) | 7.43 (4.52, 11.00) | 1.55 (1.47, 1.62) |
| El Salvador | 183.54 (112.74, 270.07) | 6.81 (4.20, 10.00) | 429.97 (270.02, 628.71) | 10.78 (6.77, 15.77) | 1.56 (1.44, 1.68) |
| Equatorial Guinea | 12.77 (7.76, 18.99) | 6.97 (4.29, 10.27) | 81.81 (49.35, 122.02) | 12.00 (7.38, 17.58) | 2.18 (1.99, 2.38) |
| Eritrea | 82.87 (51.02, 122.64) | 5.88 (3.70, 8.56) | 225.26 (140.65, 329.09) | 6.95 (4.41, 10.03) | 0.52 (0.49, 0.55) |
| Estonia | 230.38 (139.31, 330.35) | 21.35 (12.99, 30.58) | 252.57 (154.46, 358.19) | 27.30 (16.73, 38.89) | 1.15 (0.99, 1.31) |
| Eswatini | 46.33 (28.21, 67.65) | 12.82 (7.80, 18.67) | 98.44 (60.77, 143.21) | 14.60 (8.99, 21.23) | 0.40 (0.22, 0.58) |
| Ethiopia | 1337.59 (779.72, 2048.46) | 6.55 (3.87, 9.93) | 2979.00 (1737.14, 4530.59) | 6.00 (3.55, 9.03) | -0.13 (-0.22, -0.04) |
| Fiji | 12.40 (6.83, 19.62) | 2.75 (1.51, 4.37) | 19.89 (11.20, 31.07) | 3.34 (1.88, 5.22) | 0.65 (0.50, 0.80) |
| Finland | 1018.97 (722.21, 1390.95) | 27.55 (19.46, 37.71) | 1461.69 (987.42, 2004.58) | 34.37 (22.91, 47.77) | 0.93 (0.83, 1.03) |
| France | 5242.82 (3234.63, 7798.26) | 12.95 (7.97, 19.33) | 7507.21 (4628.06, 11041.10) | 15.32 (9.35, 22.63) | 0.66 (0.54, 0.78) |
| Gabon | 41.22 (25.11, 61.11) | 9.30 (5.77, 13.58) | 132.18 (81.26, 191.32) | 13.36 (8.28, 19.21) | 1.23 (1.16, 1.30) |
| Gambia | 16.03 (8.87, 24.96) | 3.74 (2.08, 5.82) | 54.91 (30.82, 85.01) | 4.73 (2.67, 7.33) | 0.85 (0.74, 0.95) |
| Georgia | 203.46 (118.17, 314.38) | 5.58 (3.24, 8.61) | 156.79 (93.38, 236.00) | 6.69 (4.01, 10.04) | 0.56 (0.47, 0.65) |
| Germany | 8416.31 (5121.93, 12617.55) | 14.10 (8.50, 21.20) | 10506.31 (6388.98, 15789.53) | 16.35 (9.80, 24.75) | 0.48 (0.37, 0.59) |
| Ghana | 242.31 (133.08, 379.42) | 3.48 (1.91, 5.45) | 912.74 (514.02, 1418.50) | 4.91 (2.77, 7.63) | 1.15 (1.02, 1.27) |
| Greece | 1001.72 (618.12, 1467.96) | 13.72 (8.45, 20.08) | 1140.17 (711.33, 1651.58) | 15.64 (9.77, 22.67) | 0.44 (0.41, 0.47) |
| Greenland | 3.89 (2.40, 5.70) | 10.76 (6.72, 15.64) | 7.52 (4.90, 10.58) | 16.68 (10.76, 23.62) | 1.53 (1.49, 1.56) |
| Grenada | 2.05 (1.20, 3.10) | 4.59 (2.68, 6.98) | 5.01 (2.95, 7.57) | 6.98 (4.10, 10.54) | 1.17 (1.08, 1.27) |
| Guam | 3.42 (1.90, 5.37) | 3.83 (2.12, 6.06) | 5.42 (3.06, 8.37) | 5.22 (2.94, 8.04) | 1.00 (0.89, 1.11) |
| Guatemala | 289.48 (178.37, 421.48) | 7.82 (4.84, 11.33) | 1307.10 (830.55, 1865.58) | 14.21 (9.04, 20.22) | 1.82 (1.68, 1.97) |
| Guinea | 98.94 (54.87, 153.16) | 3.60 (2.00, 5.56) | 261.84 (149.06, 401.36) | 4.24 (2.43, 6.48) | 0.55 (0.43, 0.68) |
| Guinea-Bissau | 15.61 (8.69, 24.28) | 3.59 (2.01, 5.56) | 42.07 (23.76, 64.43) | 4.29 (2.44, 6.52) | 0.61 (0.47, 0.74) |
| Guyana | 18.73 (11.01, 28.30) | 4.25 (2.48, 6.45) | 28.48 (17.23, 43.00) | 5.73 (3.45, 8.65) | 0.90 (0.83, 0.97) |
| Haiti | 178.56 (108.01, 266.84) | 5.45 (3.29, 8.12) | 530.31 (324.93, 778.08) | 6.78 (4.15, 9.96) | 0.71 (0.65, 0.77) |
| Honduras | 356.85 (225.86, 506.89) | 17.42 (11.06, 24.63) | 1616.59 (1079.97, 2228.35) | 28.09 (18.82, 38.63) | 1.44 (1.36, 1.53) |
| Hungary | 1080.77 (666.72, 1534.35) | 14.47 (8.94, 20.58) | 1324.87 (862.28, 1881.84) | 17.93 (11.56, 25.74) | 0.71 (0.56, 0.86) |
| Iceland | 27.11 (16.18, 40.67) | 16.97 (10.16, 25.41) | 48.84 (29.24, 73.26) | 19.40 (11.52, 29.21) | 0.49 (0.43, 0.55) |
| India | 47062.82 (28142.67, 71436.36) | 10.82 (6.49, 16.39) | 136982.18 (83652.91, 204521.60) | 15.33 (9.39, 22.85) | 1.22 (1.11, 1.34) |
| Indonesia | 2651.70 (1404.54, 4247.32) | 2.60 (1.38, 4.15) | 6466.60 (3590.33, 10136.19) | 3.29 (1.83, 5.16) | 0.71 (0.66, 0.76) |
| Iran (Islamic Republic of) | 1265.44 (734.53, 1941.25) | 4.34 (2.48, 6.71) | 3518.40 (2061.95, 5349.19) | 5.88 (3.49, 8.90) | 0.96 (0.92, 0.99) |
| Iraq | 471.84 (283.17, 714.48) | 4.83 (2.85, 7.35) | 1830.50 (1100.95, 2724.11) | 6.98 (4.17, 10.42) | 1.39 (1.20, 1.58) |
| Ireland | 774.55 (497.27, 1096.77) | 36.00 (23.17, 50.88) | 1601.22 (1004.35, 2284.81) | 41.46 (25.74, 59.78) | 0.73 (0.53, 0.92) |
| Israel | 325.37 (192.05, 492.05) | 11.35 (6.73, 17.15) | 841.08 (505.39, 1272.57) | 14.26 (8.56, 21.58) | 0.83 (0.77, 0.88) |
| Italy | 7840.30 (4652.57, 11791.96) | 18.22 (10.75, 27.49) | 8403.30 (5059.55, 12549.26) | 17.30 (10.29, 25.98) | -0.13 (-0.15, -0.11) |
| Jamaica | 77.33 (44.98, 117.91) | 6.03 (3.50, 9.21) | 168.68 (100.15, 251.43) | 8.67 (5.14, 12.92) | 1.09 (0.98, 1.21) |
| Japan | 24933.62 (15400.85, 36799.98) | 24.71 (15.16, 36.65) | 20440.23 (12544.89, 30002.01) | 21.58 (13.07, 31.91) | -0.23 (-0.36, -0.10) |
| Jordan | 95.11 (57.56, 143.12) | 4.67 (2.77, 7.08) | 598.36 (367.51, 894.64) | 7.25 (4.42, 10.88) | 1.70 (1.59, 1.81) |
| Kazakhstan | 523.16 (306.55, 799.80) | 5.12 (3.00, 7.82) | 879.56 (524.87, 1337.44) | 7.13 (4.26, 10.83) | 1.33 (1.06, 1.61) |
| Kenya | 539.56 (311.78, 834.67) | 6.13 (3.61, 9.39) | 1725.83 (1006.40, 2622.67) | 6.91 (4.08, 10.44) | 0.31 (0.22, 0.41) |
| Kiribati | 1.16 (0.65, 1.81) | 2.75 (1.53, 4.28) | 2.41 (1.41, 3.69) | 3.23 (1.87, 4.94) | 0.47 (0.41, 0.53) |
| Kuwait | 121.64 (74.93, 179.88) | 9.85 (6.01, 14.73) | 544.33 (325.96, 795.33) | 15.40 (9.47, 22.22) | 1.87 (1.61, 2.13) |
| Kyrgyzstan | 366.79 (215.06, 560.78) | 15.08 (8.79, 23.05) | 927.98 (572.20, 1387.31) | 21.89 (13.44, 32.77) | 1.33 (1.16, 1.50) |
| Lao People's Democratic Republic | 69.88 (39.12, 108.89) | 3.57 (2.02, 5.50) | 234.37 (134.12, 356.39) | 5.28 (3.04, 8.00) | 1.34 (1.26, 1.43) |
| Latvia | 291.32 (180.90, 416.19) | 16.07 (10.04, 22.93) | 259.61 (165.76, 363.36) | 20.13 (12.83, 28.26) | 0.80 (0.73, 0.88) |
| Lebanon | 131.20 (81.45, 192.79) | 7.24 (4.45, 10.71) | 379.29 (232.82, 561.06) | 10.10 (6.24, 14.91) | 1.24 (1.11, 1.38) |
| Lesotho | 76.61 (46.56, 112.00) | 10.53 (6.41, 15.37) | 136.22 (84.69, 198.54) | 12.46 (7.74, 18.10) | 0.37 (0.24, 0.50) |
| Liberia | 43.50 (24.02, 69.03) | 3.88 (2.15, 6.12) | 138.70 (77.82, 215.20) | 4.87 (2.76, 7.52) | 0.95 (0.76, 1.13) |
| Libya | 102.53 (62.10, 153.27) | 4.41 (2.63, 6.67) | 323.53 (200.57, 481.47) | 6.40 (3.97, 9.54) | 1.35 (1.29, 1.41) |
| Lithuania | 470.11 (299.53, 667.13) | 19.34 (12.36, 27.32) | 436.62 (270.46, 618.29) | 23.45 (14.67, 33.25) | 0.60 (0.57, 0.64) |
| Luxembourg | 40.64 (24.47, 60.90) | 14.33 (8.58, 21.53) | 84.76 (51.60, 126.15) | 16.72 (10.08, 24.99) | 0.59 (0.51, 0.67) |
| Madagascar | 245.40 (141.47, 379.92) | 4.90 (2.87, 7.48) | 786.60 (464.43, 1173.55) | 5.82 (3.50, 8.58) | 0.54 (0.49, 0.59) |
| Malawi | 237.18 (140.72, 357.45) | 5.96 (3.62, 8.87) | 618.18 (368.36, 928.14) | 7.33 (4.47, 10.82) | 0.72 (0.63, 0.81) |
| Malaysia | 257.22 (142.26, 404.21) | 2.72 (1.52, 4.26) | 789.02 (447.64, 1234.02) | 3.67 (2.09, 5.74) | 1.02 (0.98, 1.07) |
| Maldives | 5.72 (3.26, 8.79) | 5.93 (3.40, 9.00) | 29.29 (16.76, 44.49) | 7.65 (4.43, 11.57) | 1.08 (0.69, 1.46) |
| Mali | 129.20 (71.29, 201.94) | 3.33 (1.84, 5.19) | 420.31 (240.97, 648.15) | 4.04 (2.31, 6.19) | 0.71 (0.61, 0.81) |
| Malta | 34.85 (21.00, 51.34) | 13.76 (8.30, 20.29) | 51.15 (31.30, 75.78) | 15.99 (9.69, 23.83) | 0.49 (0.42, 0.55) |
| Marshall Islands | 0.74 (0.41, 1.16) | 3.57 (1.97, 5.55) | 1.69 (0.97, 2.53) | 4.70 (2.71, 7.04) | 0.86 (0.79, 0.92) |
| Mauritania | 38.56 (21.52, 59.99) | 4.11 (2.30, 6.35) | 124.61 (70.66, 192.35) | 5.84 (3.32, 8.98) | 1.15 (1.04, 1.27) |
| Mauritius | 29.40 (16.15, 46.67) | 4.56 (2.53, 7.23) | 68.56 (39.23, 105.47) | 6.88 (3.89, 10.63) | 1.16 (1.08, 1.23) |
| Mexico | 12209.69 (7501.44, 17614.89) | 29.15 (17.78, 42.21) | 28827.00 (18318.60, 40789.65) | 33.02 (20.98, 46.73) | 0.34 (0.24, 0.43) |
| Micronesia (Federated States of) | 2.01 (1.13, 3.12) | 3.92 (2.20, 6.09) | 3.67 (2.11, 5.65) | 5.55 (3.19, 8.55) | 1.09 (1.04, 1.13) |
| Monaco | 2.22 (1.32, 3.38) | 10.43 (6.19, 15.89) | 2.87 (1.74, 4.32) | 11.23 (6.77, 16.90) | 0.36 (0.22, 0.50) |
| Mongolia | 74.28 (44.86, 111.95) | 6.58 (3.96, 9.93) | 215.34 (130.08, 317.79) | 9.89 (5.96, 14.65) | 1.64 (1.46, 1.81) |
| Montenegro | 39.76 (23.12, 60.83) | 9.56 (5.57, 14.63) | 53.68 (31.86, 78.88) | 11.98 (7.10, 17.62) | 1.01 (0.83, 1.19) |
| Morocco | 576.40 (348.02, 859.04) | 3.95 (2.35, 5.92) | 1474.52 (912.90, 2178.29) | 5.99 (3.71, 8.84) | 1.39 (1.29, 1.50) |
| Mozambique | 321.94 (192.71, 481.52) | 5.55 (3.37, 8.20) | 864.46 (524.73, 1282.71) | 6.76 (4.18, 9.86) | 0.55 (0.50, 0.60) |
| Myanmar | 781.24 (439.21, 1196.88) | 3.61 (2.05, 5.49) | 2052.98 (1181.87, 3128.65) | 5.59 (3.22, 8.52) | 1.48 (1.38, 1.58) |
| Namibia | 63.75 (38.73, 92.99) | 9.46 (5.76, 13.76) | 172.37 (106.09, 250.64) | 12.12 (7.45, 17.60) | 0.87 (0.73, 1.00) |
| Nauru | 0.24 (0.14, 0.38) | 4.35 (2.46, 6.78) | 0.37 (0.21, 0.56) | 5.71 (3.30, 8.76) | 0.75 (0.56, 0.95) |
| Nepal | 788.78 (472.17, 1175.72) | 8.35 (5.01, 12.44) | 2527.01 (1537.71, 3811.44) | 13.47 (8.19, 20.28) | 1.64 (1.56, 1.72) |
| Netherlands | 2863.22 (1775.88, 4167.24) | 27.02 (16.76, 39.35) | 3632.51 (2244.86, 5344.00) | 26.13 (15.84, 38.89) | 0.15 (-0.05, 0.35) |
| New Zealand | 693.69 (419.68, 1038.40) | 31.48 (19.09, 47.05) | 1161.20 (712.42, 1700.35) | 29.73 (18.10, 43.74) | -0.07 (-0.15, 0.02) |
| Nicaragua | 192.95 (119.96, 282.83) | 11.34 (7.03, 16.58) | 678.13 (423.77, 981.21) | 16.43 (10.27, 23.80) | 1.31 (1.26, 1.35) |
| Niger | 107.73 (59.85, 168.84) | 3.22 (1.80, 5.03) | 376.71 (211.86, 578.08) | 3.76 (2.12, 5.72) | 0.62 (0.51, 0.73) |
| Nigeria | 1550.88 (872.23, 2419.74) | 3.56 (2.00, 5.55) | 4975.50 (2875.65, 7534.98) | 4.42 (2.55, 6.70) | 0.79 (0.63, 0.94) |
| Niue | 0.05 (0.03, 0.08) | 3.93 (2.21, 6.14) | 0.06 (0.03, 0.09) | 5.39 (3.02, 8.37) | 1.02 (0.99, 1.04) |
| North Macedonia | 85.99 (50.58, 129.92) | 6.52 (3.83, 9.84) | 152.06 (90.90, 226.06) | 9.14 (5.44, 13.61) | 1.30 (1.21, 1.39) |
| Northern Mariana Islands | 1.60 (0.88, 2.52) | 4.95 (2.72, 7.85) | 1.96 (1.12, 3.04) | 5.75 (3.27, 8.90) | 0.29 (0.17, 0.40) |
| Norway | 978.71 (609.20, 1439.16) | 34.22 (21.29, 50.34) | 1194.36 (735.66, 1769.04) | 28.27 (17.23, 42.17) | -0.50 (-0.62, -0.38) |
| Oman | 35.03 (20.58, 53.20) | 3.21 (1.86, 4.92) | 195.15 (117.49, 292.35) | 5.85 (3.55, 8.75) | 1.97 (1.92, 2.01) |
| Pakistan | 6968.68 (4377.84, 10147.17) | 12.30 (7.56, 18.14) | 16351.76 (10271.96, 23795.82) | 11.55 (7.15, 16.98) | 0.15 (-0.01, 0.31) |
| Palau | 0.44 (0.24, 0.68) | 4.56 (2.53, 7.08) | 0.81 (0.45, 1.25) | 5.87 (3.30, 9.11) | 0.74 (0.70, 0.78) |
| Palestine | 49.15 (29.52, 74.11) | 4.94 (2.92, 7.47) | 219.39 (132.76, 327.70) | 7.14 (4.28, 10.71) | 1.19 (1.10, 1.29) |
| Panama | 134.12 (81.89, 194.29) | 10.33 (6.34, 14.87) | 387.00 (238.13, 555.70) | 14.08 (8.65, 20.23) | 0.99 (0.96, 1.02) |
| Papua New Guinea | 59.19 (32.58, 92.72) | 2.68 (1.48, 4.20) | 194.59 (110.77, 298.78) | 3.18 (1.80, 4.88) | 0.47 (0.41, 0.54) |
| Paraguay | 272.81 (156.61, 416.58) | 12.79 (7.34, 19.55) | 1036.82 (608.58, 1535.51) | 22.33 (13.11, 33.08) | 1.54 (1.39, 1.68) |
| Peru | 2561.95 (1648.21, 3663.50) | 19.92 (12.56, 28.86) | 8146.52 (5093.81, 11843.40) | 33.78 (21.08, 49.16) | 1.94 (1.83, 2.06) |
| Philippines | 2191.93 (1269.87, 3363.85) | 6.70 (3.86, 10.31) | 4203.58 (2425.58, 6464.07) | 5.92 (3.41, 9.11) | -0.16 (-0.25, -0.07) |
| Poland | 5460.35 (3353.24, 7866.33) | 21.48 (13.28, 30.85) | 5646.40 (3550.51, 8107.99) | 19.91 (12.49, 28.66) | -0.12 (-0.22, -0.02) |
| Portugal | 956.81 (599.72, 1380.76) | 13.69 (8.54, 19.79) | 1557.50 (954.36, 2268.82) | 19.40 (11.76, 28.47) | 1.17 (1.04, 1.30) |
| Puerto Rico | 206.53 (119.50, 320.24) | 9.17 (5.31, 14.23) | 325.42 (182.72, 517.12) | 14.61 (8.24, 23.09) | 1.57 (1.49, 1.65) |
| Qatar | 14.45 (8.41, 22.11) | 4.64 (2.70, 7.16) | 194.35 (112.26, 297.93) | 8.07 (4.71, 12.45) | 1.45 (1.25, 1.65) |
| Republic of Korea | 3593.25 (2326.62, 5035.29) | 12.71 (8.23, 17.79) | 8490.71 (5577.30, 11814.25) | 18.47 (12.09, 25.83) | 1.38 (1.20, 1.55) |
| Republic of Moldova | 178.75 (107.26, 266.26) | 6.23 (3.74, 9.27) | 239.33 (144.14, 357.91) | 8.90 (5.35, 13.34) | 1.20 (1.03, 1.38) |
| Romania | 991.04 (585.63, 1513.14) | 6.32 (3.73, 9.65) | 1279.75 (770.27, 1875.92) | 9.51 (5.71, 14.00) | 1.46 (1.38, 1.55) |
| Russian Federation | 12283.67 (7569.04, 17776.10) | 12.00 (7.42, 17.34) | 13990.79 (8772.77, 19881.54) | 14.13 (8.87, 20.10) | 0.58 (0.55, 0.62) |
| Rwanda | 179.63 (107.81, 269.31) | 6.28 (3.89, 9.21) | 534.34 (324.16, 792.08) | 8.20 (5.08, 11.98) | 1.11 (0.97, 1.26) |
| Saint Kitts and Nevis | 1.71 (1.00, 2.60) | 7.86 (4.62, 11.96) | 5.23 (3.09, 7.84) | 11.47 (6.78, 17.20) | 1.22 (1.07, 1.37) |
| Saint Lucia | 4.97 (2.94, 7.54) | 6.92 (4.07, 10.54) | 12.41 (7.34, 18.74) | 9.52 (5.66, 14.32) | 0.99 (0.84, 1.14) |
| Saint Vincent and the Grenadines | 2.80 (1.64, 4.31) | 4.81 (2.80, 7.43) | 4.90 (2.92, 7.44) | 6.35 (3.80, 9.61) | 0.98 (0.93, 1.03) |
| Samoa | 3.62 (2.02, 5.68) | 4.14 (2.30, 6.50) | 6.32 (3.62, 9.75) | 5.25 (3.00, 8.09) | 0.67 (0.62, 0.73) |
| San Marino | 1.67 (1.00, 2.56) | 10.12 (6.05, 15.48) | 2.50 (1.52, 3.76) | 10.74 (6.47, 16.17) | 0.31 (0.20, 0.41) |
| Sao Tome and Principe | 2.18 (1.20, 3.44) | 4.12 (2.27, 6.48) | 6.45 (3.63, 9.95) | 5.36 (3.05, 8.25) | 0.92 (0.79, 1.06) |
| Saudi Arabia | 336.19 (199.34, 502.66) | 3.70 (2.16, 5.60) | 1890.71 (1140.00, 2841.22) | 6.43 (3.91, 9.65) | 1.92 (1.79, 2.06) |
| Senegal | 126.73 (70.01, 197.51) | 3.86 (2.15, 5.98) | 378.37 (213.22, 586.94) | 4.77 (2.71, 7.37) | 0.66 (0.56, 0.76) |
| Serbia | 601.06 (404.06, 821.68) | 8.51 (5.72, 11.60) | 856.42 (528.65, 1251.80) | 12.84 (7.89, 18.85) | 1.38 (1.22, 1.54) |
| Seychelles | 1.38 (0.76, 2.17) | 3.32 (1.84, 5.22) | 3.39 (1.90, 5.19) | 4.44 (2.49, 6.82) | 0.90 (0.85, 0.95) |
| Sierra Leone | 70.43 (38.40, 111.24) | 3.61 (1.98, 5.69) | 186.87 (106.21, 286.23) | 4.22 (2.41, 6.42) | 0.50 (0.36, 0.64) |
| Singapore | 173.82 (104.45, 256.68) | 8.18 (4.94, 12.09) | 507.12 (305.96, 748.02) | 10.86 (6.51, 16.11) | 1.03 (0.96, 1.09) |
| Slovakia | 248.22 (146.41, 371.39) | 7.21 (4.27, 10.79) | 406.93 (246.54, 599.56) | 10.14 (6.12, 14.99) | 1.14 (1.09, 1.20) |
| Slovenia | 249.62 (148.10, 368.69) | 17.29 (10.24, 25.62) | 429.55 (268.24, 611.70) | 25.16 (15.45, 36.38) | 1.44 (1.35, 1.54) |
| Solomon Islands | 5.86 (3.36, 8.99) | 3.56 (2.04, 5.45) | 18.55 (10.71, 28.27) | 4.73 (2.73, 7.20) | 0.82 (0.76, 0.87) |
| Somalia | 202.60 (124.66, 297.68) | 6.37 (4.02, 9.21) | 541.61 (330.89, 795.84) | 6.50 (4.10, 9.36) | 0.07 (0.04, 0.11) |
| South Africa | 5319.69 (3559.81, 7370.39) | 23.88 (15.70, 33.55) | 8183.57 (5365.18, 11516.91) | 20.86 (13.58, 29.53) | -0.28 (-0.38, -0.18) |
| South Sudan | 129.25 (74.32, 196.78) | 5.30 (3.11, 7.96) | 273.18 (162.10, 410.43) | 6.18 (3.71, 9.20) | 0.55 (0.50, 0.60) |
| Spain | 3916.14 (2849.04, 5080.90) | 14.62 (10.63, 18.93) | 6296.67 (5066.45, 7746.50) | 16.87 (13.52, 20.79) | 0.39 (0.34, 0.44) |
| Sri Lanka | 292.14 (160.36, 465.06) | 2.86 (1.58, 4.54) | 595.14 (336.65, 920.84) | 3.90 (2.20, 6.05) | 1.12 (0.98, 1.25) |
| Sudan | 355.62 (217.55, 526.00) | 3.40 (2.04, 5.06) | 1339.64 (835.43, 1950.78) | 5.19 (3.20, 7.59) | 1.48 (1.34, 1.62) |
| Suriname | 9.94 (5.79, 15.11) | 4.34 (2.50, 6.62) | 21.51 (12.73, 32.73) | 5.58 (3.31, 8.47) | 0.93 (0.84, 1.02) |
| Sweden | 1563.61 (1012.54, 2262.72) | 25.88 (16.75, 37.41) | 1701.88 (1099.04, 2449.55) | 22.55 (14.52, 32.52) | -0.40 (-0.44, -0.36) |
| Switzerland | 949.06 (570.47, 1437.76) | 18.80 (11.26, 28.52) | 1452.72 (868.89, 2185.13) | 20.27 (12.00, 30.63) | 0.33 (0.26, 0.39) |
| Syrian Arab Republic | 286.04 (172.18, 433.66) | 4.41 (2.61, 6.74) | 647.54 (389.36, 983.51) | 7.31 (4.41, 11.08) | 1.73 (1.57, 1.89) |
| Taiwan (Province of China) | 1645.34 (992.99, 2439.77) | 13.15 (7.97, 19.43) | 2660.90 (2320.65, 3041.78) | 12.64 (11.04, 14.39) | 0.43 (0.21, 0.66) |
| Tajikistan | 214.23 (129.78, 319.08) | 7.78 (4.64, 11.65) | 616.33 (379.66, 909.90) | 9.99 (6.13, 14.81) | 0.72 (0.64, 0.79) |
| Thailand | 1739.28 (988.44, 2680.48) | 5.13 (2.94, 7.86) | 4147.42 (2412.38, 6290.17) | 7.57 (4.35, 11.55) | 1.35 (1.27, 1.43) |
| Timor-Leste | 12.73 (7.06, 20.00) | 3.36 (1.90, 5.25) | 33.37 (19.09, 51.66) | 4.81 (2.79, 7.39) | 1.21 (1.17, 1.24) |
| Togo | 57.54 (31.47, 90.47) | 3.76 (2.06, 5.87) | 200.83 (112.92, 310.05) | 4.60 (2.60, 7.07) | 0.68 (0.53, 0.83) |
| Tokelau | 0.03 (0.02, 0.05) | 3.51 (1.98, 5.45) | 0.04 (0.02, 0.06) | 4.90 (2.78, 7.64) | 1.05 (1.01, 1.10) |
| Tonga | 1.97 (1.10, 3.09) | 3.89 (2.15, 6.09) | 2.98 (1.70, 4.62) | 4.98 (2.83, 7.72) | 0.70 (0.65, 0.75) |
| Trinidad and Tobago | 71.47 (42.12, 107.33) | 10.22 (6.00, 15.34) | 137.10 (81.04, 206.55) | 13.84 (8.19, 20.86) | 1.14 (1.07, 1.22) |
| Tunisia | 211.19 (128.97, 316.90) | 4.32 (2.60, 6.52) | 517.64 (311.90, 771.54) | 6.55 (3.98, 9.73) | 1.48 (1.36, 1.59) |
| Turkiye | 3211.20 (1965.99, 4682.51) | 9.71 (5.89, 14.19) | 9226.95 (5614.26, 13502.23) | 15.79 (9.66, 23.11) | 1.76 (1.69, 1.84) |
| Turkmenistan | 90.06 (52.84, 137.26) | 4.47 (2.60, 6.83) | 200.70 (121.56, 296.53) | 5.98 (3.62, 8.84) | 1.10 (0.94, 1.26) |
| Tuvalu | 0.18 (0.10, 0.29) | 3.40 (1.90, 5.25) | 0.35 (0.20, 0.54) | 4.55 (2.59, 7.02) | 0.85 (0.81, 0.89) |
| Uganda | 369.26 (217.16, 561.63) | 5.67 (3.42, 8.45) | 1233.08 (743.67, 1846.81) | 7.13 (4.40, 10.51) | 0.85 (0.73, 0.97) |
| Ukraine | 1843.39 (1045.96, 2841.89) | 5.18 (2.95, 7.95) | 2034.54 (1178.69, 3088.79) | 6.61 (3.85, 10.00) | 0.74 (0.71, 0.77) |
| United Arab Emirates | 59.35 (34.67, 89.66) | 4.65 (2.70, 7.09) | 565.63 (327.07, 862.36) | 7.78 (4.68, 11.72) | 1.59 (1.27, 1.91) |
| United Kingdom | 11838.90 (7701.83, 17059.00) | 29.61 (19.23, 42.71) | 16197.03 (10689.54, 22993.58) | 31.65 (20.71, 45.20) | 0.24 (0.20, 0.28) |
| United Republic of Tanzania | 622.17 (360.84, 948.53) | 5.97 (3.53, 9.00) | 1969.73 (1166.81, 2960.32) | 7.38 (4.43, 10.96) | 0.62 (0.56, 0.68) |
| United States of America | 29769.30 (19639.56, 41120.65) | 17.79 (11.75, 24.58) | 53161.14 (35553.83, 72682.18) | 21.07 (14.12, 28.73) | 0.74 (0.66, 0.82) |
| United States Virgin Islands | 4.29 (2.48, 6.59) | 6.35 (3.67, 9.74) | 4.57 (2.65, 7.10) | 8.31 (4.85, 12.74) | 0.88 (0.85, 0.92) |
| Uruguay | 201.38 (120.36, 304.39) | 10.18 (6.08, 15.37) | 343.55 (207.94, 511.20) | 14.79 (8.93, 22.03) | 1.18 (1.13, 1.23) |
| Uzbekistan | 1864.52 (1115.11, 2731.67) | 16.74 (9.92, 24.46) | 4762.65 (3058.12, 6742.87) | 20.68 (13.22, 29.38) | 0.85 (0.73, 0.96) |
| Vanuatu | 2.56 (1.43, 3.94) | 3.33 (1.85, 5.15) | 7.52 (4.29, 11.43) | 4.18 (2.38, 6.35) | 0.69 (0.65, 0.73) |
| Venezuela (Bolivarian Republic of) | 1605.27 (985.87, 2297.24) | 16.57 (10.26, 23.66) | 4473.50 (2839.04, 6365.19) | 24.13 (15.25, 34.49) | 1.18 (1.04, 1.32) |
| Viet Nam | 1817.76 (1030.14, 2834.59) | 5.34 (3.05, 8.31) | 6386.80 (3690.14, 9830.61) | 8.91 (5.14, 13.71) | 1.63 (1.59, 1.66) |
| Yemen | 194.91 (118.30, 289.03) | 3.15 (1.89, 4.70) | 773.38 (476.55, 1126.83) | 4.10 (2.50, 6.00) | 1.03 (0.93, 1.13) |
| Zambia | 187.48 (112.65, 278.54) | 5.92 (3.64, 8.65) | 678.22 (412.89, 1003.84) | 7.73 (4.81, 11.27) | 0.94 (0.77, 1.10) |
| Zimbabwe | 351.79 (217.73, 514.73) | 7.29 (4.49, 10.63) | 632.31 (395.14, 909.24) | 7.44 (4.63, 10.72) | -0.24 (-0.41, -0.07) |
