# Supplementary material for: Global burden and regional disparities of rheumatoid arthritis among the working-age population: A comprehensive analysis from 1990 to 2021 with projections to 2040
Source: PLoS One. 2025 Jun 4;20(6):e0325127. doi: 10.1371/journal.pone.0325127 (PMC12136291; doi:10.1371/journal.pone.0325127)
Supplement: S2 Table — (DOCX) [file pone.0325127.s017.docx]

**S2 Table.** National trends in the burden of rheumatoid arthritis prevalence among working-age population: 1990−2021

| **Location** | **1990** | | **2021** | | **EAPC (95% CI)** |
| --- | --- | --- | --- | --- | --- |
|  | **Number** | **ASR** | **Number** | **ASR** |  |
| Afghanistan | 3402.59 (2649.05, 4270.10) | 75.07 (58.41, 94.05) | 12161.08 (9353.68, 15283.00) | 93.82 (74.37, 115.19) | 0.82 (0.68, 0.95) |
| Albania | 2038.14 (1530.58, 2661.29) | 115.91 (88.21, 149.83) | 3960.63 (3070.26, 5000.12) | 188.05 (143.34, 240.06) | 1.79 (1.72, 1.86) |
| Algeria | 7251.94 (5475.20, 9399.36) | 66.13 (50.78, 84.57) | 29362.42 (22842.77, 36970.35) | 102.82 (80.06, 129.33) | 1.56 (1.45, 1.66) |
| American Samoa | 21.60 (15.91, 28.52) | 89.13 (67.02, 115.82) | 36.63 (28.03, 46.29) | 111.18 (84.36, 141.39) | 0.61 (0.55, 0.68) |
| Andorra | 71.01 (53.82, 91.06) | 180.25 (137.07, 230.33) | 159.00 (124.56, 198.69) | 207.14 (159.31, 262.66) | 0.56 (0.51, 0.60) |
| Angola | 3976.10 (3000.75, 5120.69) | 98.97 (76.83, 124.53) | 16084.46 (12191.68, 20438.56) | 128.05 (99.78, 159.04) | 0.94 (0.85, 1.03) |
| Antigua and Barbuda | 36.63 (27.28, 47.80) | 114.16 (86.31, 147.22) | 112.50 (85.89, 143.66) | 160.98 (121.91, 207.02) | 1.05 (0.97, 1.14) |
| Argentina | 35025.86 (27074.42, 44111.06) | 175.47 (135.52, 221.11) | 83856.74 (66375.21, 103187.08) | 269.59 (212.84, 332.52) | 1.27 (1.15, 1.38) |
| Armenia | 2046.76 (1539.80, 2649.55) | 96.34 (72.53, 124.48) | 3339.78 (2618.55, 4196.34) | 146.42 (113.29, 185.87) | 1.49 (1.44, 1.55) |
| Australia | 30341.48 (22929.14, 38799.05) | 269.55 (203.71, 344.53) | 65122.44 (49848.68, 82247.17) | 326.89 (247.16, 417.18) | 0.71 (0.55, 0.86) |
| Austria | 15680.45 (12057.13, 19667.88) | 276.65 (211.10, 348.95) | 22707.96 (17733.60, 28238.19) | 304.19 (233.28, 384.01) | 0.37 (0.24, 0.50) |
| Azerbaijan | 3547.44 (2659.94, 4634.24) | 86.18 (65.21, 111.82) | 8908.71 (6845.58, 11424.52) | 111.13 (84.81, 143.10) | 1.12 (0.83, 1.40) |
| Bahamas | 185.23 (138.47, 240.59) | 134.93 (103.12, 171.96) | 508.73 (397.97, 637.72) | 175.42 (136.40, 221.15) | 0.91 (0.79, 1.02) |
| Bahrain | 412.37 (300.57, 545.57) | 152.67 (116.08, 194.85) | 2937.49 (2220.33, 3757.46) | 249.87 (191.97, 316.04) | 1.52 (1.45, 1.59) |
| Bangladesh | 48835.91 (37153.74, 62202.67) | 107.15 (83.34, 134.61) | 173988.55 (135366.64, 218241.43) | 170.44 (133.27, 213.00) | 1.73 (1.52, 1.93) |
| Barbados | 260.21 (196.77, 334.33) | 176.66 (134.84, 224.95) | 597.36 (469.24, 743.00) | 243.75 (187.23, 308.57) | 0.94 (0.84, 1.04) |
| Belarus | 7912.54 (6030.11, 10118.82) | 104.41 (78.66, 134.56) | 12477.64 (9743.05, 15758.57) | 157.76 (120.49, 202.75) | 1.42 (1.28, 1.57) |
| Belgium | 18374.81 (14099.74, 23498.96) | 245.83 (186.60, 316.81) | 25883.40 (20044.14, 32605.90) | 285.95 (217.16, 365.39) | 0.54 (0.50, 0.58) |
| Belize | 96.77 (72.48, 124.48) | 128.53 (98.91, 161.90) | 482.21 (368.32, 613.18) | 187.52 (144.83, 236.18) | 1.26 (1.20, 1.33) |
| Benin | 1086.66 (767.96, 1467.42) | 60.87 (44.13, 80.65) | 4263.28 (3081.75, 5668.18) | 77.24 (57.30, 100.59) | 0.83 (0.75, 0.92) |
| Bermuda | 61.65 (46.21, 80.49) | 143.50 (107.81, 186.89) | 104.46 (80.47, 133.43) | 194.32 (146.44, 252.98) | 1.08 (1.00, 1.17) |
| Bhutan | 379.23 (278.25, 499.07) | 144.82 (109.16, 187.12) | 1051.49 (794.72, 1357.48) | 220.75 (168.54, 282.75) | 1.52 (1.37, 1.67) |
| Bolivia (Plurinational State of) | 6430.33 (4901.57, 8132.24) | 223.20 (173.48, 278.05) | 24810.78 (19411.51, 30895.85) | 352.16 (277.90, 435.70) | 1.57 (1.53, 1.60) |
| Bosnia and Herzegovina | 6331.59 (4965.52, 7851.15) | 200.08 (156.58, 248.62) | 8223.13 (6614.37, 9997.88) | 291.88 (231.57, 358.67) | 1.49 (1.39, 1.59) |
| Botswana | 883.38 (678.38, 1125.02) | 170.22 (133.79, 212.38) | 3334.30 (2601.96, 4187.94) | 229.44 (181.90, 284.49) | 1.05 (0.90, 1.21) |
| Brazil | 181902.66 (140205.40, 228362.52) | 224.59 (175.31, 279.16) | 347781.95 (275377.81, 427985.18) | 220.98 (174.26, 272.80) | 0.20 (0.12, 0.28) |
| Brunei Darussalam | 221.13 (164.74, 290.33) | 190.92 (147.76, 242.69) | 806.75 (617.58, 1019.99) | 249.59 (192.86, 313.47) | 0.88 (0.75, 1.01) |
| Bulgaria | 8237.36 (6378.58, 10402.90) | 125.05 (95.34, 159.76) | 8384.01 (6587.38, 10431.70) | 153.69 (118.38, 194.38) | 0.69 (0.53, 0.84) |
| Burkina Faso | 2105.16 (1503.31, 2810.35) | 57.13 (41.49, 75.28) | 6539.79 (4676.28, 8679.84) | 70.52 (51.63, 92.06) | 0.77 (0.69, 0.85) |
| Burundi | 1651.42 (1220.26, 2175.02) | 81.92 (62.51, 104.92) | 4506.59 (3310.39, 5917.85) | 87.55 (66.54, 111.68) | 0.29 (0.24, 0.34) |
| Cabo Verde | 89.22 (64.51, 118.46) | 64.19 (47.06, 84.06) | 305.20 (225.43, 400.94) | 84.05 (62.46, 109.93) | 0.98 (0.87, 1.09) |
| Cambodia | 2445.92 (1755.73, 3239.99) | 57.34 (42.20, 74.56) | 8604.77 (6364.32, 11219.53) | 84.11 (62.65, 108.97) | 1.27 (1.23, 1.32) |
| Cameroon | 3086.41 (2206.27, 4154.31) | 71.95 (52.46, 95.38) | 12168.21 (8745.53, 16109.38) | 87.12 (64.21, 113.16) | 0.65 (0.48, 0.83) |
| Canada | 48105.60 (45233.58, 51021.19) | 256.80 (241.47, 272.35) | 107152.46 (100885.63, 113676.59) | 349.22 (328.36, 370.90) | 0.99 (0.87, 1.10) |
| Central African Republic | 1219.06 (937.77, 1540.50) | 110.86 (87.39, 137.21) | 2796.58 (2166.87, 3486.17) | 116.38 (92.34, 142.38) | 0.20 (0.16, 0.24) |
| Chad | 1191.07 (845.10, 1603.03) | 52.13 (37.78, 68.95) | 3688.11 (2649.89, 4917.87) | 58.69 (43.36, 76.46) | 0.44 (0.30, 0.58) |
| Chile | 17874.03 (13636.26, 22613.63) | 239.49 (184.71, 300.00) | 59101.71 (46642.94, 73278.22) | 410.68 (320.45, 513.72) | 1.78 (1.69, 1.87) |
| China | 1596525.79 (1218913.99, 2041931.44) | 232.96 (179.71, 295.33) | 3213945.13 (2543960.36, 3984362.44) | 272.01 (212.39, 341.13) | 0.59 (0.55, 0.63) |
| Colombia | 30249.70 (23192.93, 38085.07) | 194.33 (152.48, 239.92) | 102148.14 (81615.07, 124652.43) | 297.17 (236.62, 363.53) | 1.38 (1.22, 1.54) |
| Comoros | 153.83 (112.61, 203.60) | 86.51 (64.97, 112.02) | 416.01 (310.24, 541.40) | 101.26 (76.59, 130.31) | 0.58 (0.50, 0.67) |
| Congo | 1266.40 (961.53, 1615.67) | 136.12 (105.76, 169.96) | 4706.86 (3630.44, 6017.66) | 170.04 (134.10, 213.73) | 0.83 (0.75, 0.92) |
| Cook Islands | 6.93 (5.06, 9.25) | 67.00 (49.45, 88.86) | 12.17 (9.29, 15.65) | 96.30 (72.48, 125.20) | 1.19 (1.14, 1.24) |
| Costa Rica | 3487.18 (2665.34, 4486.80) | 252.79 (198.00, 318.29) | 14424.29 (11525.23, 17731.73) | 419.22 (333.32, 517.29) | 1.55 (1.47, 1.63) |
| Cote d'Ivoire | 2926.44 (2067.58, 3954.10) | 60.75 (44.19, 80.44) | 9828.17 (7047.93, 13042.50) | 76.67 (56.23, 100.03) | 0.79 (0.65, 0.93) |
| Croatia | 7799.53 (5940.47, 9908.69) | 200.30 (150.30, 257.68) | 10901.44 (8489.24, 13493.87) | 303.14 (230.62, 382.16) | 1.41 (1.39, 1.44) |
| Cuba | 11612.26 (8872.18, 14791.22) | 173.43 (133.71, 219.39) | 23745.45 (18907.14, 29149.75) | 248.74 (193.61, 311.77) | 1.16 (1.08, 1.24) |
| Cyprus | 1421.89 (1079.53, 1827.96) | 288.47 (219.57, 370.08) | 3947.28 (2998.75, 5007.79) | 361.29 (272.67, 460.61) | 0.95 (0.79, 1.11) |
| Czechia | 12602.24 (9914.07, 15774.53) | 171.85 (134.17, 216.49) | 19472.91 (15415.49, 24103.11) | 234.94 (183.09, 294.14) | 0.98 (0.94, 1.01) |
| Democratic People's Republic of Korea | 23216.40 (17880.04, 29117.84) | 177.82 (137.33, 222.58) | 50446.31 (39941.06, 62163.92) | 231.22 (181.27, 287.76) | 0.88 (0.84, 0.93) |
| Democratic Republic of the Congo | 14278.31 (10690.22, 18539.38) | 96.99 (74.29, 123.40) | 42068.32 (31938.46, 54091.27) | 111.46 (86.76, 140.42) | 0.42 (0.31, 0.54) |
| Denmark | 8806.63 (7028.31, 10903.39) | 234.15 (186.09, 290.87) | 14768.35 (11683.60, 18437.67) | 315.74 (245.60, 399.54) | 1.11 (1.04, 1.19) |
| Djibouti | 127.18 (91.56, 171.08) | 78.75 (58.91, 102.67) | 684.71 (505.72, 890.64) | 99.14 (74.77, 126.73) | 0.79 (0.67, 0.91) |
| Dominica | 42.92 (32.43, 55.31) | 119.62 (91.65, 152.57) | 73.97 (57.58, 92.88) | 151.04 (116.54, 191.01) | 0.67 (0.60, 0.73) |
| Dominican Republic | 2698.09 (1973.81, 3603.04) | 78.98 (58.95, 104.03) | 7393.38 (5588.19, 9653.69) | 105.17 (79.77, 137.01) | 0.98 (0.92, 1.03) |
| Ecuador | 10052.73 (7659.02, 12748.13) | 218.12 (169.17, 272.28) | 39597.04 (30990.25, 49169.65) | 357.32 (280.74, 442.13) | 1.53 (1.44, 1.62) |
| Egypt | 25118.82 (19234.62, 32171.14) | 90.17 (70.04, 114.18) | 90620.25 (70242.60, 113859.77) | 148.12 (115.72, 185.09) | 1.50 (1.43, 1.58) |
| El Salvador | 2794.82 (2120.36, 3598.34) | 118.10 (91.26, 149.84) | 7204.31 (5636.92, 9068.04) | 187.14 (147.11, 234.65) | 1.56 (1.44, 1.69) |
| Equatorial Guinea | 173.29 (131.33, 221.77) | 101.37 (78.15, 127.91) | 1023.19 (763.03, 1326.71) | 175.24 (136.11, 219.51) | 2.20 (2.01, 2.40) |
| Eritrea | 1038.71 (776.61, 1353.28) | 81.21 (62.67, 103.10) | 2796.65 (2096.12, 3629.19) | 94.40 (73.00, 119.34) | 0.46 (0.44, 0.48) |
| Estonia | 4124.47 (3416.28, 4956.99) | 347.20 (283.37, 422.99) | 4646.18 (3846.07, 5521.20) | 436.53 (354.25, 528.03) | 1.13 (0.96, 1.29) |
| Eswatini | 652.73 (501.25, 827.23) | 219.61 (173.54, 271.97) | 1440.87 (1123.83, 1821.00) | 250.98 (200.78, 310.44) | 0.38 (0.22, 0.53) |
| Ethiopia | 17260.24 (12661.66, 22904.35) | 92.66 (69.72, 120.36) | 38352.21 (27901.38, 51027.04) | 87.16 (65.67, 112.73) | -0.06 (-0.14, 0.03) |
| Fiji | 217.54 (156.18, 291.23) | 54.23 (39.67, 71.72) | 408.60 (307.88, 531.71) | 68.10 (51.25, 88.68) | 0.73 (0.61, 0.85) |
| Finland | 13340.83 (10941.85, 16179.03) | 354.25 (289.06, 431.38) | 19744.24 (16125.38, 23966.75) | 448.59 (359.89, 553.65) | 0.94 (0.84, 1.05) |
| France | 72555.14 (55611.06, 92811.00) | 177.03 (134.88, 227.49) | 111610.48 (87052.57, 140596.03) | 219.60 (168.71, 279.42) | 0.82 (0.73, 0.91) |
| Gabon | 558.84 (428.10, 713.40) | 132.54 (102.78, 167.11) | 1785.82 (1368.58, 2253.70) | 191.04 (148.58, 238.21) | 1.25 (1.18, 1.32) |
| Gambia | 220.15 (153.27, 299.41) | 58.51 (41.89, 77.87) | 772.39 (553.64, 1032.85) | 75.46 (55.56, 98.89) | 0.92 (0.83, 1.01) |
| Georgia | 4018.87 (3061.28, 5225.43) | 105.18 (79.53, 137.40) | 3366.27 (2635.54, 4244.81) | 125.34 (96.53, 160.25) | 0.55 (0.47, 0.64) |
| Germany | 139156.94 (106859.24, 176345.06) | 224.22 (170.52, 286.33) | 187252.66 (145084.32, 234910.51) | 269.11 (204.17, 343.50) | 0.62 (0.53, 0.70) |
| Ghana | 3506.45 (2474.42, 4756.76) | 55.89 (40.29, 74.67) | 13809.56 (10001.81, 18453.59) | 81.13 (59.77, 106.98) | 1.26 (1.14, 1.37) |
| Greece | 17953.48 (14314.76, 22125.28) | 232.48 (183.83, 288.13) | 22160.12 (17836.92, 26923.23) | 272.95 (216.52, 335.42) | 0.53 (0.52, 0.55) |
| Greenland | 55.49 (42.14, 70.53) | 159.34 (122.90, 200.07) | 115.69 (93.77, 141.04) | 242.44 (192.65, 300.56) | 1.48 (1.44, 1.52) |
| Grenada | 33.45 (24.90, 43.63) | 83.25 (62.60, 107.76) | 93.45 (71.31, 119.34) | 125.09 (94.86, 160.43) | 1.14 (1.06, 1.23) |
| Guam | 60.02 (43.73, 80.26) | 74.25 (55.03, 98.07) | 113.04 (86.11, 144.36) | 101.49 (76.34, 130.79) | 1.04 (0.96, 1.13) |
| Guatemala | 4190.17 (3200.40, 5322.84) | 128.59 (100.19, 160.73) | 19390.04 (15323.89, 24289.69) | 229.98 (184.41, 284.73) | 1.79 (1.65, 1.93) |
| Guinea | 1477.34 (1060.23, 1968.90) | 57.40 (41.81, 75.76) | 3754.28 (2711.94, 4999.26) | 68.02 (50.35, 88.91) | 0.56 (0.44, 0.67) |
| Guinea-Bissau | 223.28 (159.04, 300.20) | 57.68 (42.18, 76.05) | 598.34 (428.49, 800.68) | 68.87 (50.67, 90.10) | 0.60 (0.47, 0.72) |
| Guyana | 290.33 (213.73, 380.90) | 77.78 (58.70, 100.45) | 518.27 (398.86, 662.29) | 106.58 (82.24, 135.96) | 0.95 (0.89, 1.01) |
| Haiti | 2766.67 (2116.72, 3538.55) | 94.46 (73.60, 118.99) | 8400.60 (6508.17, 10563.93) | 118.23 (93.09, 146.64) | 0.72 (0.66, 0.79) |
| Honduras | 4897.26 (3908.06, 6047.46) | 276.93 (225.72, 335.59) | 22634.40 (18578.55, 27316.11) | 436.64 (363.82, 519.77) | 1.39 (1.30, 1.48) |
| Hungary | 17316.58 (13904.99, 20964.36) | 217.09 (172.05, 265.69) | 21460.55 (17402.65, 26044.15) | 267.68 (213.75, 329.79) | 0.70 (0.58, 0.82) |
| Iceland | 412.13 (311.60, 531.53) | 260.30 (197.08, 335.14) | 804.44 (614.16, 1021.65) | 306.27 (230.86, 392.72) | 0.60 (0.55, 0.65) |
| India | 602793.04 (450883.72, 793285.38) | 142.58 (107.92, 185.97) | 1751208.90 (1332863.55, 2276931.59) | 197.05 (150.75, 255.12) | 1.14 (1.00, 1.28) |
| Indonesia | 40903.64 (28663.74, 55724.73) | 43.41 (31.04, 58.30) | 108380.91 (78918.39, 143373.97) | 54.65 (39.78, 72.33) | 0.72 (0.67, 0.77) |
| Iran (Islamic Republic of) | 20579.26 (15294.48, 26772.04) | 82.07 (61.90, 105.36) | 72431.07 (56112.12, 91073.70) | 115.77 (89.70, 145.70) | 1.11 (1.09, 1.13) |
| Iraq | 7763.50 (5818.58, 10119.40) | 98.42 (75.50, 125.68) | 32760.54 (24861.59, 41462.49) | 136.75 (105.09, 171.67) | 1.25 (1.07, 1.43) |
| Ireland | 9153.09 (7119.17, 11469.28) | 424.26 (329.99, 531.38) | 19706.64 (15335.28, 24727.69) | 502.79 (387.10, 637.25) | 0.75 (0.58, 0.91) |
| Israel | 5369.71 (4048.66, 6960.14) | 192.21 (145.47, 248.36) | 14427.56 (11039.98, 18409.64) | 240.98 (183.82, 308.30) | 0.82 (0.78, 0.86) |
| Italy | 110510.15 (84206.05, 142779.60) | 250.94 (189.36, 326.81) | 126777.40 (97689.74, 162237.76) | 245.77 (186.13, 319.57) | -0.04 (-0.05, -0.02) |
| Jamaica | 1205.91 (898.33, 1562.49) | 106.63 (80.79, 136.41) | 2983.91 (2297.16, 3768.67) | 153.36 (118.00, 193.72) | 1.11 (1.01, 1.22) |
| Japan | 315261.68 (239671.15, 405282.94) | 304.64 (229.96, 394.43) | 292161.03 (224552.07, 370116.95) | 290.52 (220.06, 373.58) | 0.02 (-0.10, 0.13) |
| Jordan | 1501.82 (1129.79, 1952.00) | 93.91 (72.52, 119.23) | 11031.41 (8522.36, 13963.77) | 144.06 (112.26, 181.18) | 1.67 (1.56, 1.78) |
| Kazakhstan | 9452.86 (7097.56, 12264.53) | 96.95 (73.14, 125.21) | 17225.56 (13188.85, 22058.33) | 133.80 (101.99, 171.93) | 1.28 (1.02, 1.53) |
| Kenya | 6766.37 (4857.70, 9128.73) | 86.51 (64.20, 113.61) | 22599.04 (16574.82, 29921.47) | 99.07 (74.61, 128.45) | 0.39 (0.28, 0.50) |
| Kiribati | 20.33 (14.93, 26.91) | 54.88 (41.17, 71.62) | 45.70 (34.52, 58.84) | 65.25 (49.72, 83.44) | 0.48 (0.43, 0.54) |
| Kuwait | 2083.79 (1594.97, 2640.21) | 194.13 (151.88, 242.86) | 13087.69 (10467.93, 15882.14) | 329.55 (263.17, 400.54) | 2.13 (1.87, 2.39) |
| Kyrgyzstan | 5850.88 (4568.67, 7321.74) | 265.65 (209.72, 328.79) | 15327.07 (12232.39, 18894.31) | 376.97 (302.26, 462.38) | 1.24 (1.12, 1.36) |
| Lao People's Democratic Republic | 1059.59 (768.29, 1402.62) | 58.42 (43.26, 76.12) | 3530.50 (2606.56, 4597.52) | 83.12 (62.24, 107.10) | 1.21 (1.14, 1.29) |
| Latvia | 5505.61 (4581.06, 6510.60) | 269.61 (220.49, 323.69) | 5249.96 (4393.38, 6154.77) | 334.87 (272.56, 401.87) | 0.81 (0.74, 0.88) |
| Lebanon | 2587.12 (2036.18, 3232.58) | 153.90 (121.68, 191.48) | 8336.56 (6599.16, 10284.11) | 219.90 (174.50, 271.00) | 1.33 (1.21, 1.45) |
| Lesotho | 1170.44 (908.00, 1463.32) | 177.15 (139.39, 218.93) | 2014.02 (1582.34, 2514.49) | 211.55 (169.82, 259.63) | 0.40 (0.29, 0.52) |
| Liberia | 633.55 (452.75, 855.29) | 63.47 (46.40, 84.19) | 2051.61 (1468.26, 2723.77) | 79.98 (58.67, 104.14) | 1.00 (0.81, 1.20) |
| Libya | 1653.70 (1259.76, 2140.94) | 87.12 (67.67, 111.24) | 6561.78 (5134.57, 8257.74) | 128.64 (100.91, 161.70) | 1.44 (1.37, 1.51) |
| Lithuania | 8881.72 (7374.05, 10566.68) | 334.65 (274.60, 401.99) | 9205.91 (7786.66, 10791.82) | 396.77 (325.69, 478.22) | 0.55 (0.49, 0.61) |
| Luxembourg | 656.59 (504.23, 834.25) | 224.53 (170.96, 286.87) | 1403.83 (1083.26, 1772.49) | 263.26 (200.65, 335.54) | 0.61 (0.55, 0.68) |
| Madagascar | 3277.78 (2387.96, 4355.38) | 71.77 (53.66, 93.16) | 10340.17 (7621.34, 13499.17) | 83.85 (63.64, 107.00) | 0.50 (0.45, 0.54) |
| Malawi | 3075.44 (2236.58, 4051.13) | 85.57 (64.25, 109.94) | 7809.30 (5732.70, 10230.53) | 104.29 (79.51, 132.62) | 0.69 (0.62, 0.76) |
| Malaysia | 3900.21 (2780.58, 5249.69) | 44.53 (32.41, 58.96) | 12862.45 (9427.40, 17058.91) | 60.70 (44.69, 80.19) | 1.04 (1.00, 1.09) |
| Maldives | 83.94 (62.60, 109.01) | 95.30 (72.51, 121.72) | 436.45 (322.03, 569.39) | 120.29 (90.66, 154.25) | 1.00 (0.60, 1.40) |
| Mali | 1909.44 (1356.09, 2553.96) | 53.17 (38.38, 70.21) | 5968.64 (4309.65, 7942.14) | 65.21 (48.26, 85.03) | 0.75 (0.66, 0.85) |
| Malta | 556.69 (422.31, 704.73) | 216.28 (163.82, 274.32) | 880.94 (680.07, 1109.93) | 257.98 (195.53, 329.30) | 0.56 (0.49, 0.63) |
| Marshall Islands | 11.22 (8.06, 14.96) | 64.50 (48.16, 83.55) | 29.35 (22.17, 37.23) | 85.77 (65.47, 107.99) | 0.90 (0.83, 0.96) |
| Mauritania | 562.27 (404.58, 753.32) | 66.63 (48.99, 87.74) | 1818.94 (1315.85, 2402.28) | 93.81 (69.20, 122.10) | 1.13 (1.02, 1.24) |
| Mauritius | 435.06 (310.00, 588.78) | 71.13 (51.46, 95.08) | 1132.58 (845.43, 1476.82) | 107.67 (79.04, 141.92) | 1.23 (1.19, 1.28) |
| Mexico | 164362.72 (127732.03, 205689.99) | 450.37 (355.73, 555.37) | 466104.99 (376785.31, 564208.85) | 530.12 (428.41, 642.19) | 0.44 (0.32, 0.57) |
| Micronesia (Federated States of) | 32.12 (23.50, 42.29) | 72.98 (54.75, 94.00) | 66.26 (50.50, 83.91) | 103.39 (78.98, 130.65) | 1.07 (1.02, 1.13) |
| Monaco | 44.44 (34.40, 56.76) | 189.86 (144.89, 244.97) | 61.85 (48.56, 77.38) | 211.02 (162.58, 268.49) | 0.44 (0.33, 0.55) |
| Mongolia | 1135.51 (861.82, 1459.45) | 122.23 (95.24, 153.45) | 3926.89 (3071.85, 4917.42) | 181.39 (142.15, 226.82) | 1.56 (1.41, 1.71) |
| Montenegro | 668.63 (507.65, 861.26) | 158.08 (119.53, 204.12) | 956.98 (746.66, 1209.90) | 195.37 (150.24, 249.86) | 0.93 (0.77, 1.09) |
| Morocco | 9917.47 (7508.89, 12726.41) | 78.96 (60.80, 100.07) | 29877.61 (23429.25, 37438.33) | 119.67 (93.73, 150.10) | 1.39 (1.29, 1.49) |
| Mozambique | 4302.70 (3190.61, 5630.16) | 79.53 (60.22, 102.32) | 11080.41 (8179.12, 14478.58) | 96.80 (73.92, 122.90) | 0.57 (0.53, 0.62) |
| Myanmar | 11847.51 (8602.80, 15560.25) | 58.90 (43.62, 76.22) | 32078.19 (24003.30, 41342.18) | 87.67 (65.66, 112.90) | 1.38 (1.29, 1.47) |
| Namibia | 907.63 (695.39, 1147.63) | 157.42 (123.17, 195.17) | 2537.18 (1942.82, 3197.27) | 198.25 (154.50, 246.39) | 0.80 (0.69, 0.92) |
| Nauru | 3.96 (2.89, 5.20) | 80.15 (59.80, 103.55) | 6.09 (4.54, 7.90) | 105.08 (79.92, 134.54) | 0.76 (0.56, 0.95) |
| Nepal | 11250.61 (8399.85, 14562.54) | 128.09 (97.13, 164.02) | 37074.86 (28151.01, 48038.70) | 206.85 (158.44, 266.19) | 1.69 (1.56, 1.82) |
| Netherlands | 35162.05 (26741.87, 44737.21) | 330.10 (250.58, 420.55) | 50066.45 (38647.38, 63352.94) | 352.02 (266.64, 451.85) | 0.39 (0.25, 0.54) |
| New Zealand | 8155.88 (6163.09, 10653.77) | 370.56 (280.11, 483.66) | 15310.90 (11801.13, 19474.89) | 380.82 (290.02, 488.86) | 0.18 (0.08, 0.29) |
| Nicaragua | 2646.24 (2027.68, 3362.90) | 183.71 (144.35, 228.39) | 10241.14 (8028.05, 12816.19) | 263.98 (208.92, 327.56) | 1.29 (1.22, 1.36) |
| Niger | 1532.30 (1080.64, 2071.63) | 51.23 (37.05, 67.88) | 5284.74 (3766.86, 7034.24) | 60.73 (44.47, 79.12) | 0.65 (0.55, 0.75) |
| Nigeria | 22584.29 (16164.09, 30463.33) | 57.45 (41.92, 76.40) | 74687.79 (54847.62, 98253.58) | 75.44 (56.66, 97.75) | 0.98 (0.83, 1.13) |
| Niue | 0.93 (0.69, 1.21) | 74.21 (55.33, 96.59) | 1.23 (0.94, 1.55) | 102.57 (77.07, 131.43) | 1.04 (1.02, 1.06) |
| North Macedonia | 1508.60 (1136.49, 1955.38) | 113.33 (85.24, 147.04) | 2815.09 (2158.98, 3570.86) | 154.60 (117.00, 198.15) | 1.19 (1.10, 1.28) |
| Northern Mariana Islands | 26.58 (19.07, 35.40) | 92.88 (68.63, 121.46) | 41.52 (31.36, 53.10) | 109.93 (81.63, 142.48) | 0.36 (0.24, 0.47) |
| Norway | 11044.35 (8411.62, 14285.75) | 382.07 (290.16, 495.35) | 15454.22 (11850.19, 19844.51) | 357.42 (270.86, 463.54) | -0.19 (-0.31, -0.06) |
| Oman | 570.67 (425.37, 747.02) | 61.49 (46.89, 79.15) | 3568.68 (2723.68, 4603.53) | 110.36 (85.16, 140.88) | 2.04 (2.00, 2.09) |
| Pakistan | 112605.86 (87689.48, 141587.80) | 230.51 (181.81, 287.10) | 279268.12 (217336.92, 350861.36) | 219.95 (173.38, 274.18) | 0.20 (0.06, 0.33) |
| Palau | 7.32 (5.38, 9.55) | 84.74 (63.60, 109.07) | 17.40 (13.43, 21.81) | 111.28 (84.26, 142.01) | 0.81 (0.77, 0.85) |
| Palestine | 792.98 (588.96, 1029.57) | 99.60 (75.72, 126.78) | 3676.79 (2786.36, 4704.22) | 136.44 (104.96, 172.38) | 0.99 (0.88, 1.11) |
| Panama | 1909.08 (1456.45, 2425.27) | 163.04 (126.74, 203.99) | 6222.97 (4865.19, 7739.80) | 225.19 (175.93, 280.33) | 1.04 (1.02, 1.06) |
| Papua New Guinea | 980.39 (712.77, 1306.33) | 50.40 (37.50, 66.20) | 3375.17 (2489.24, 4407.89) | 59.98 (45.01, 77.46) | 0.49 (0.42, 0.56) |
| Paraguay | 4383.46 (3256.03, 5681.72) | 236.78 (179.92, 301.52) | 17576.85 (13904.94, 21881.15) | 402.91 (322.21, 497.32) | 1.52 (1.39, 1.64) |
| Peru | 44164.78 (35559.03, 53653.12) | 410.38 (335.03, 493.12) | 160664.90 (130530.61, 194796.08) | 681.38 (555.23, 824.11) | 1.87 (1.75, 1.99) |
| Philippines | 33652.26 (25078.79, 43915.35) | 114.89 (87.11, 147.90) | 69347.47 (52008.17, 90036.83) | 101.07 (76.34, 130.59) | -0.13 (-0.23, -0.03) |
| Poland | 88167.24 (69809.67, 108878.77) | 333.16 (262.49, 413.08) | 98097.12 (78312.85, 119976.30) | 311.96 (245.15, 386.74) | -0.09 (-0.20, 0.01) |
| Portugal | 15544.76 (12265.18, 19204.78) | 215.53 (168.36, 268.32) | 27406.80 (21831.92, 33449.23) | 313.34 (244.49, 388.80) | 1.24 (1.11, 1.37) |
| Puerto Rico | 3548.08 (2687.34, 4552.72) | 160.66 (122.08, 205.72) | 6370.28 (4901.65, 8084.82) | 253.02 (190.60, 326.88) | 1.53 (1.44, 1.62) |
| Qatar | 239.11 (176.85, 314.98) | 84.35 (63.65, 109.27) | 3504.61 (2600.13, 4592.21) | 145.92 (109.98, 189.08) | 1.45 (1.22, 1.67) |
| Republic of Korea | 51440.97 (41114.00, 62613.30) | 192.46 (155.18, 232.45) | 129320.88 (105376.50, 155622.48) | 260.40 (209.49, 317.15) | 1.10 (0.97, 1.23) |
| Republic of Moldova | 3260.05 (2507.43, 4124.98) | 111.59 (85.49, 141.42) | 4838.95 (3810.19, 6087.27) | 158.60 (122.86, 202.17) | 1.22 (1.04, 1.39) |
| Romania | 18212.15 (13737.24, 23693.28) | 109.67 (81.90, 143.78) | 24069.75 (18586.03, 30394.90) | 160.03 (121.53, 204.59) | 1.36 (1.27, 1.45) |
| Russian Federation | 244567.97 (198290.70, 297457.74) | 222.00 (178.45, 271.77) | 307462.29 (253958.42, 367772.63) | 263.38 (213.91, 319.81) | 0.62 (0.56, 0.67) |
| Rwanda | 2341.63 (1748.80, 3062.04) | 89.98 (69.20, 114.57) | 6887.70 (5178.75, 8943.52) | 114.35 (88.24, 145.21) | 1.00 (0.87, 1.13) |
| Saint Kitts and Nevis | 26.29 (19.87, 33.91) | 136.75 (105.09, 174.01) | 95.08 (74.18, 118.44) | 192.59 (148.25, 242.86) | 1.10 (0.97, 1.22) |
| Saint Lucia | 76.59 (57.78, 98.60) | 123.48 (95.13, 156.35) | 243.96 (191.38, 305.79) | 170.85 (132.60, 216.29) | 1.01 (0.85, 1.16) |
| Saint Vincent and the Grenadines | 43.49 (32.15, 57.07) | 87.36 (65.78, 112.94) | 95.52 (73.44, 122.31) | 115.92 (88.40, 149.45) | 1.00 (0.95, 1.04) |
| Samoa | 59.41 (43.64, 78.28) | 79.31 (59.51, 102.83) | 111.99 (84.09, 144.95) | 98.41 (74.40, 126.62) | 0.60 (0.55, 0.65) |
| San Marino | 30.48 (23.21, 39.33) | 180.22 (136.74, 233.29) | 52.47 (41.16, 66.23) | 200.89 (154.85, 256.59) | 0.47 (0.39, 0.55) |
| Sao Tome and Principe | 32.02 (22.89, 43.02) | 66.63 (48.32, 88.53) | 96.26 (69.67, 126.68) | 86.19 (63.53, 111.98) | 0.90 (0.78, 1.03) |
| Saudi Arabia | 5393.60 (4035.38, 7029.28) | 71.18 (54.58, 91.35) | 37214.84 (28586.61, 47261.76) | 127.11 (98.46, 160.64) | 1.99 (1.87, 2.12) |
| Senegal | 1807.22 (1284.14, 2430.83) | 61.53 (44.70, 81.25) | 5499.32 (3917.99, 7317.36) | 76.08 (55.30, 99.75) | 0.67 (0.57, 0.76) |
| Serbia | 9452.33 (7990.35, 11067.52) | 126.92 (106.43, 149.66) | 14009.85 (10952.02, 17414.99) | 193.62 (148.76, 244.03) | 1.35 (1.15, 1.56) |
| Seychelles | 22.71 (16.47, 30.58) | 59.30 (43.63, 78.83) | 64.46 (48.27, 83.50) | 79.77 (59.11, 104.08) | 0.92 (0.88, 0.97) |
| Sierra Leone | 1009.66 (713.75, 1364.43) | 57.30 (41.48, 76.15) | 2654.34 (1899.88, 3566.45) | 67.30 (49.40, 88.74) | 0.51 (0.38, 0.64) |
| Singapore | 2660.97 (2000.26, 3447.84) | 132.74 (101.36, 170.12) | 8905.93 (6820.56, 11296.55) | 175.89 (132.98, 225.54) | 0.97 (0.92, 1.03) |
| Slovakia | 4363.68 (3330.57, 5562.93) | 124.64 (94.82, 159.11) | 7545.78 (5863.79, 9431.12) | 169.81 (129.71, 215.15) | 1.03 (0.98, 1.09) |
| Slovenia | 3564.07 (2725.78, 4497.57) | 238.40 (180.47, 303.29) | 6166.45 (4766.69, 7644.77) | 333.55 (252.02, 421.75) | 1.28 (1.21, 1.35) |
| Solomon Islands | 92.40 (68.50, 119.99) | 65.33 (49.68, 83.25) | 313.19 (235.35, 400.54) | 88.02 (67.39, 111.05) | 0.87 (0.81, 0.92) |
| Somalia | 2540.25 (1880.47, 3319.07) | 90.06 (69.35, 114.00) | 6625.48 (4913.51, 8672.07) | 91.76 (71.40, 115.54) | 0.08 (0.06, 0.10) |
| South Africa | 82127.64 (66247.24, 99551.08) | 451.04 (368.70, 540.46) | 143975.16 (117228.41, 174452.64) | 384.11 (313.91, 464.05) | -0.36 (-0.44, -0.27) |
| South Sudan | 1686.92 (1216.87, 2244.84) | 77.42 (57.74, 100.31) | 3725.68 (2762.86, 4881.78) | 91.54 (69.45, 117.79) | 0.60 (0.54, 0.66) |
| Spain | 58384.74 (50508.61, 66573.12) | 211.36 (182.17, 241.73) | 99331.05 (89179.14, 110871.78) | 248.46 (221.03, 279.41) | 0.45 (0.39, 0.51) |
| Sri Lanka | 4765.87 (3429.92, 6445.70) | 49.67 (36.29, 66.46) | 10708.47 (7994.02, 14004.20) | 67.55 (49.96, 88.95) | 1.08 (0.97, 1.19) |
| Sudan | 5867.87 (4443.47, 7551.40) | 66.62 (51.49, 84.53) | 22706.16 (17535.59, 28679.99) | 103.03 (81.01, 128.28) | 1.52 (1.37, 1.68) |
| Suriname | 166.78 (124.45, 216.86) | 80.08 (60.42, 103.31) | 419.43 (321.50, 537.98) | 103.50 (78.85, 133.31) | 0.93 (0.84, 1.02) |
| Sweden | 21506.72 (17090.73, 26643.10) | 349.31 (276.33, 434.46) | 24615.64 (19612.41, 30491.33) | 315.64 (248.75, 394.28) | -0.33 (-0.37, -0.28) |
| Switzerland | 13298.34 (9982.06, 17321.40) | 259.81 (194.21, 339.30) | 21665.57 (16548.64, 27846.09) | 291.28 (219.13, 377.99) | 0.45 (0.42, 0.49) |
| Syrian Arab Republic | 4642.16 (3478.07, 6028.64) | 87.90 (67.30, 112.33) | 12985.46 (9927.08, 16573.97) | 140.88 (107.02, 180.97) | 1.61 (1.48, 1.74) |
| Taiwan (Province of China) | 21234.07 (15910.44, 27188.87) | 173.35 (130.56, 220.57) | 37548.67 (35940.07, 39219.51) | 169.01 (161.26, 177.06) | 0.46 (0.27, 0.66) |
| Tajikistan | 3348.26 (2567.41, 4242.12) | 142.51 (111.09, 178.03) | 10508.23 (8237.61, 13114.13) | 182.15 (143.98, 225.77) | 0.68 (0.64, 0.73) |
| Thailand | 25535.04 (18747.01, 33362.67) | 79.73 (59.45, 102.74) | 71043.69 (53988.69, 90076.01) | 118.96 (88.55, 153.73) | 1.38 (1.30, 1.45) |
| Timor-Leste | 185.22 (131.62, 248.52) | 53.73 (39.47, 70.44) | 473.43 (344.96, 630.86) | 72.92 (54.12, 95.83) | 1.04 (1.00, 1.08) |
| Togo | 801.49 (560.74, 1086.12) | 59.52 (42.91, 78.85) | 2973.59 (2145.01, 3945.43) | 73.34 (53.91, 95.90) | 0.70 (0.56, 0.83) |
| Tokelau | 0.54 (0.40, 0.70) | 65.35 (48.77, 84.72) | 0.78 (0.59, 1.01) | 92.12 (69.63, 118.81) | 1.10 (1.06, 1.14) |
| Tonga | 33.56 (24.68, 44.33) | 74.40 (55.49, 97.26) | 54.79 (40.68, 71.14) | 96.49 (72.07, 124.81) | 0.71 (0.66, 0.77) |
| Trinidad and Tobago | 1095.31 (833.13, 1395.36) | 174.87 (135.60, 219.67) | 2569.37 (2032.47, 3191.81) | 237.71 (185.45, 298.83) | 1.15 (1.08, 1.23) |
| Tunisia | 3693.80 (2806.94, 4746.62) | 86.65 (66.72, 110.19) | 11172.15 (8705.80, 14030.99) | 131.02 (101.41, 165.57) | 1.47 (1.35, 1.58) |
| Turkiye | 53276.12 (42544.59, 65592.20) | 177.44 (143.13, 216.61) | 169295.22 (137862.18, 204616.91) | 279.13 (226.43, 338.58) | 1.60 (1.55, 1.66) |
| Turkmenistan | 1457.07 (1083.11, 1901.75) | 84.39 (63.86, 108.68) | 3780.87 (2925.24, 4801.55) | 114.26 (88.55, 144.90) | 1.16 (1.01, 1.31) |
| Tuvalu | 3.29 (2.44, 4.28) | 63.23 (47.25, 81.86) | 6.36 (4.80, 8.15) | 85.59 (64.77, 109.27) | 0.89 (0.85, 0.93) |
| Uganda | 4716.36 (3440.14, 6237.52) | 80.45 (60.67, 103.46) | 15560.41 (11376.51, 20424.86) | 101.09 (76.66, 128.81) | 0.83 (0.72, 0.94) |
| Ukraine | 37052.65 (28035.66, 48052.21) | 94.73 (70.72, 123.98) | 43131.79 (33240.91, 54598.55) | 120.20 (90.91, 154.33) | 0.75 (0.72, 0.78) |
| United Arab Emirates | 924.82 (676.22, 1208.70) | 83.62 (63.36, 106.49) | 11740.65 (9036.36, 14819.36) | 132.55 (101.69, 168.84) | 1.55 (1.32, 1.78) |
| United Kingdom | 173600.16 (138628.19, 215107.93) | 426.43 (338.73, 530.62) | 245954.99 (198879.20, 300497.12) | 464.91 (371.55, 573.60) | 0.29 (0.25, 0.33) |
| United Republic of Tanzania | 8113.56 (5947.62, 10623.95) | 85.17 (64.13, 109.13) | 25540.27 (18927.18, 33541.40) | 104.31 (79.45, 134.07) | 0.62 (0.55, 0.69) |
| United States of America | 431555.83 (360040.42, 511707.03) | 259.31 (216.34, 307.43) | 795664.84 (670615.59, 931103.59) | 305.41 (256.31, 358.48) | 0.85 (0.74, 0.96) |
| United States Virgin Islands | 78.15 (58.45, 101.40) | 115.22 (86.28, 149.48) | 101.11 (78.84, 127.36) | 153.13 (116.62, 196.16) | 0.94 (0.89, 0.98) |
| Uruguay | 3445.77 (2660.72, 4377.18) | 169.10 (129.61, 215.97) | 5983.56 (4655.53, 7486.67) | 244.06 (187.93, 307.97) | 1.19 (1.15, 1.24) |
| Uzbekistan | 28874.25 (22766.10, 35841.48) | 311.68 (251.91, 378.30) | 87260.97 (71444.68, 104757.50) | 387.46 (318.31, 464.25) | 0.87 (0.78, 0.96) |
| Vanuatu | 41.06 (29.61, 54.07) | 61.33 (45.43, 79.36) | 129.54 (96.13, 166.43) | 78.00 (58.80, 99.18) | 0.76 (0.72, 0.79) |
| Venezuela (Bolivarian Republic of) | 21750.64 (16828.47, 27078.26) | 255.81 (203.03, 312.13) | 72479.73 (58849.84, 88561.67) | 372.05 (299.77, 457.76) | 1.18 (1.03, 1.33) |
| Viet Nam | 27065.85 (19800.59, 35513.94) | 86.78 (64.43, 112.46) | 98271.77 (73412.65, 126944.95) | 134.07 (99.72, 173.74) | 1.40 (1.35, 1.45) |
| Yemen | 3189.11 (2397.65, 4120.47) | 60.83 (46.68, 77.46) | 12852.84 (9757.33, 16410.45) | 79.74 (61.81, 100.24) | 1.03 (0.94, 1.12) |
| Zambia | 2469.49 (1836.09, 3205.99) | 88.37 (67.99, 111.64) | 8625.52 (6427.71, 11228.44) | 112.43 (87.24, 141.80) | 0.82 (0.67, 0.97) |
| Zimbabwe | 5092.09 (3896.16, 6498.53) | 129.19 (101.33, 161.35) | 9657.63 (7492.68, 12095.66) | 132.53 (105.06, 163.20) | -0.23 (-0.39, -0.06) |
