# Supplementary material for: Global burden and regional disparities of rheumatoid arthritis among the working-age population: A comprehensive analysis from 1990 to 2021 with projections to 2040
Source: PLoS One. 2025 Jun 4;20(6):e0325127. doi: 10.1371/journal.pone.0325127 (PMC12136291; doi:10.1371/journal.pone.0325127)
Supplement: S3 Table — (DOCX) [file pone.0325127.s018.docx]

**S3 Table.** National trends in the burden of rheumatoid arthritis deaths among working-age population: 1990−2021

| **Location** | **1990** | | **2021** | | **EAPC (95% CI)** |
| --- | --- | --- | --- | --- | --- |
|  | **Number** | **ASR** | **Number** | **ASR** |  |
| Afghanistan | 7.69 (2.49, 17.55) | 0.15 (0.05, 0.35) | 20.75 (6.52, 47.71) | 0.22 (0.08, 0.48) | 1.58 (1.30, 1.86) |
| Albania | 1.94 (1.18, 2.93) | 0.12 (0.08, 0.19) | 1.33 (0.79, 2.19) | 0.05 (0.03, 0.08) | -2.84 (-3.19, -2.50) |
| Algeria | 3.35 (1.46, 6.42) | 0.03 (0.02, 0.07) | 11.72 (5.82, 21.12) | 0.04 (0.02, 0.08) | 1.17 (0.90, 1.45) |
| American Samoa | 0.00 (0.00, 0.00) | 0.00 (0.00, 0.00) | 0.00 (0.00, 0.00) | 0.00 (0.00, 0.00) | -2.71 (-4.65, -0.73) |
| Andorra | 0.01 (0.00, 0.01) | 0.02 (0.01, 0.04) | 0.01 (0.00, 0.02) | 0.01 (0.00, 0.02) | -2.45 (-2.68, -2.21) |
| Angola | 0.98 (0.21, 10.13) | 0.03 (0.01, 0.28) | 1.70 (0.31, 20.20) | 0.02 (0.00, 0.18) | -2.15 (-2.27, -2.03) |
| Antigua and Barbuda | 0.03 (0.02, 0.04) | 0.10 (0.08, 0.13) | 0.08 (0.06, 0.10) | 0.11 (0.09, 0.14) | 0.04 (-0.26, 0.34) |
| Argentina | 45.53 (37.81, 54.30) | 0.22 (0.19, 0.27) | 56.66 (46.85, 68.42) | 0.18 (0.15, 0.22) | 0.07 (-0.44, 0.58) |
| Armenia | 0.04 (0.04, 0.05) | 0.00 (0.00, 0.00) | 0.80 (0.67, 0.95) | 0.03 (0.03, 0.04) | 7.26 (5.29, 9.27) |
| Australia | 27.10 (22.91, 31.88) | 0.24 (0.20, 0.28) | 22.37 (18.69, 26.83) | 0.10 (0.08, 0.12) | -2.64 (-3.03, -2.25) |
| Austria | 10.71 (8.96, 12.57) | 0.18 (0.15, 0.21) | 5.23 (4.36, 6.16) | 0.06 (0.05, 0.07) | -2.90 (-3.23, -2.57) |
| Azerbaijan | 0.05 (0.03, 0.09) | 0.00 (0.00, 0.00) | 0.33 (0.09, 0.49) | 0.00 (0.00, 0.01) | 2.50 (1.25, 3.75) |
| Bahamas | 0.21 (0.16, 0.27) | 0.19 (0.14, 0.24) | 0.41 (0.29, 0.56) | 0.14 (0.10, 0.18) | -1.28 (-1.61, -0.94) |
| Bahrain | 0.03 (0.02, 0.06) | 0.02 (0.01, 0.03) | 0.75 (0.43, 1.18) | 0.07 (0.04, 0.12) | 6.59 (5.51, 7.67) |
| Bangladesh | 63.39 (32.34, 104.76) | 0.19 (0.10, 0.31) | 99.57 (50.20, 177.25) | 0.11 (0.05, 0.19) | -1.81 (-1.90, -1.73) |
| Barbados | 0.24 (0.19, 0.31) | 0.17 (0.13, 0.22) | 0.53 (0.36, 0.72) | 0.18 (0.12, 0.25) | 0.38 (0.17, 0.59) |
| Belarus | 1.09 (0.92, 1.28) | 0.01 (0.01, 0.02) | 1.41 (1.06, 1.83) | 0.02 (0.01, 0.02) | -0.64 (-1.06, -0.22) |
| Belgium | 11.70 (9.91, 13.66) | 0.14 (0.12, 0.16) | 8.18 (6.77, 9.77) | 0.07 (0.06, 0.09) | -2.08 (-2.30, -1.86) |
| Belize | 0.08 (0.06, 0.13) | 0.12 (0.09, 0.19) | 0.40 (0.30, 0.52) | 0.17 (0.13, 0.22) | 0.57 (0.11, 1.04) |
| Benin | 0.00 (0.00, 0.00) | 0.00 (0.00, 0.00) | 0.00 (0.00, 0.00) | 0.00 (0.00, 0.00) | -1.58 (-1.71, -1.45) |
| Bermuda | 0.04 (0.03, 0.05) | 0.09 (0.07, 0.12) | 0.04 (0.03, 0.05) | 0.05 (0.04, 0.07) | -2.42 (-2.86, -1.99) |
| Bhutan | 0.35 (0.15, 0.61) | 0.18 (0.08, 0.32) | 0.47 (0.21, 0.86) | 0.12 (0.05, 0.22) | -1.59 (-1.66, -1.52) |
| Bolivia (Plurinational State of) | 8.02 (4.70, 13.64) | 0.33 (0.20, 0.55) | 15.48 (8.16, 26.27) | 0.24 (0.13, 0.40) | -1.14 (-1.21, -1.07) |
| Bosnia and Herzegovina | 4.44 (2.69, 6.71) | 0.13 (0.08, 0.20) | 2.70 (1.62, 4.30) | 0.08 (0.05, 0.12) | -1.95 (-2.14, -1.77) |
| Botswana | 1.48 (0.66, 2.61) | 0.32 (0.15, 0.56) | 2.09 (0.62, 3.83) | 0.17 (0.05, 0.30) | -2.11 (-2.43, -1.80) |
| Brazil | 100.71 (93.48, 108.53) | 0.14 (0.13, 0.16) | 222.30 (204.17, 244.90) | 0.14 (0.13, 0.15) | 0.07 (-0.23, 0.36) |
| Brunei Darussalam | 0.32 (0.17, 0.55) | 0.41 (0.23, 0.68) | 0.76 (0.42, 1.26) | 0.26 (0.14, 0.43) | -1.33 (-1.62, -1.04) |
| Bulgaria | 4.46 (3.80, 5.25) | 0.06 (0.05, 0.07) | 2.07 (1.62, 2.67) | 0.03 (0.02, 0.04) | -2.54 (-3.10, -1.98) |
| Burkina Faso | 0.00 (0.00, 0.01) | 0.00 (0.00, 0.00) | 0.00 (0.00, 0.01) | 0.00 (0.00, 0.00) | -1.24 (-1.41, -1.06) |
| Burundi | 0.33 (0.08, 3.15) | 0.02 (0.00, 0.18) | 0.38 (0.07, 4.21) | 0.01 (0.00, 0.09) | -3.16 (-3.37, -2.94) |
| Cabo Verde | 0.00 (0.00, 0.00) | 0.00 (0.00, 0.00) | 0.00 (0.00, 0.00) | 0.00 (0.00, 0.00) | -3.85 (-4.08, -3.62) |
| Cambodia | 3.97 (1.99, 6.88) | 0.11 (0.05, 0.18) | 7.54 (3.68, 12.82) | 0.08 (0.04, 0.13) | -1.18 (-1.25, -1.11) |
| Cameroon | 0.00 (0.00, 0.01) | 0.00 (0.00, 0.00) | 0.00 (0.00, 0.01) | 0.00 (0.00, 0.00) | -2.22 (-2.37, -2.07) |
| Canada | 34.00 (28.44, 40.22) | 0.18 (0.15, 0.22) | 40.12 (33.55, 48.01) | 0.11 (0.09, 0.13) | -1.78 (-1.99, -1.57) |
| Central African Republic | 0.37 (0.08, 3.74) | 0.04 (0.01, 0.37) | 0.45 (0.08, 5.12) | 0.02 (0.00, 0.24) | -2.04 (-2.19, -1.90) |
| Chad | 0.00 (0.00, 0.00) | 0.00 (0.00, 0.00) | 0.00 (0.00, 0.01) | 0.00 (0.00, 0.00) | -1.00 (-1.17, -0.83) |
| Chile | 29.88 (25.09, 35.47) | 0.43 (0.36, 0.51) | 31.71 (26.35, 37.96) | 0.20 (0.17, 0.24) | -1.96 (-2.33, -1.59) |
| China | 1864.32 (1462.87, 2431.63) | 0.29 (0.23, 0.38) | 2084.00 (1495.00, 2705.11) | 0.16 (0.11, 0.21) | -1.54 (-1.92, -1.16) |
| Colombia | 45.81 (38.79, 54.16) | 0.34 (0.29, 0.40) | 76.84 (57.82, 100.18) | 0.22 (0.16, 0.28) | -0.88 (-1.55, -0.20) |
| Comoros | 0.03 (0.01, 0.26) | 0.02 (0.00, 0.16) | 0.03 (0.01, 0.40) | 0.01 (0.00, 0.10) | -2.31 (-2.52, -2.10) |
| Congo | 0.31 (0.08, 3.38) | 0.04 (0.01, 0.39) | 0.41 (0.07, 5.19) | 0.02 (0.00, 0.21) | -2.84 (-3.03, -2.65) |
| Cook Islands | 0.00 (0.00, 0.00) | 0.00 (0.00, 0.00) | 0.00 (0.00, 0.00) | 0.00 (0.00, 0.00) | 2.39 (1.66, 3.13) |
| Costa Rica | 2.99 (2.41, 3.67) | 0.25 (0.20, 0.30) | 9.05 (7.05, 11.35) | 0.25 (0.20, 0.32) | -0.14 (-0.34, 0.06) |
| Cote d'Ivoire | 0.00 (0.00, 0.00) | 0.00 (0.00, 0.00) | 0.00 (0.00, 0.01) | 0.00 (0.00, 0.00) | -1.20 (-1.38, -1.03) |
| Croatia | 8.18 (7.08, 9.42) | 0.19 (0.16, 0.22) | 3.15 (2.54, 3.79) | 0.07 (0.06, 0.09) | -3.26 (-3.78, -2.74) |
| Cuba | 11.53 (8.78, 14.46) | 0.18 (0.14, 0.23) | 18.08 (13.79, 23.27) | 0.16 (0.12, 0.21) | -0.71 (-1.06, -0.37) |
| Cyprus | 1.63 (1.01, 2.54) | 0.34 (0.21, 0.53) | 1.49 (0.95, 2.27) | 0.13 (0.08, 0.20) | -3.15 (-3.28, -3.03) |
| Czechia | 8.74 (7.16, 10.45) | 0.11 (0.09, 0.13) | 3.77 (2.95, 4.86) | 0.04 (0.03, 0.05) | -3.05 (-3.90, -2.19) |
| Democratic People's Republic of Korea | 38.17 (21.44, 64.88) | 0.30 (0.17, 0.51) | 69.39 (39.61, 110.59) | 0.31 (0.17, 0.49) | 0.18 (-0.00, 0.36) |
| Democratic Republic of the Congo | 3.86 (0.80, 42.90) | 0.03 (0.01, 0.32) | 5.27 (0.98, 59.03) | 0.02 (0.00, 0.18) | -1.94 (-2.02, -1.86) |
| Denmark | 8.19 (6.81, 9.75) | 0.21 (0.17, 0.25) | 5.80 (4.79, 6.87) | 0.11 (0.09, 0.13) | -2.52 (-2.80, -2.24) |
| Djibouti | 0.02 (0.00, 0.15) | 0.01 (0.00, 0.11) | 0.04 (0.01, 0.44) | 0.01 (0.00, 0.07) | -1.97 (-2.18, -1.75) |
| Dominica | 0.05 (0.03, 0.08) | 0.16 (0.09, 0.24) | 0.07 (0.04, 0.11) | 0.13 (0.07, 0.20) | -0.79 (-0.95, -0.64) |
| Dominican Republic | 4.26 (2.35, 6.75) | 0.14 (0.08, 0.23) | 6.53 (3.56, 10.99) | 0.10 (0.05, 0.16) | -0.43 (-0.79, -0.07) |
| Ecuador | 13.00 (11.10, 15.24) | 0.32 (0.27, 0.37) | 20.04 (14.32, 27.48) | 0.19 (0.13, 0.26) | -2.06 (-2.73, -1.38) |
| Egypt | 9.62 (5.18, 16.16) | 0.04 (0.02, 0.07) | 18.64 (10.93, 29.80) | 0.03 (0.02, 0.05) | -0.04 (-0.32, 0.24) |
| El Salvador | 2.06 (1.22, 3.28) | 0.10 (0.06, 0.16) | 3.00 (1.78, 4.84) | 0.08 (0.05, 0.13) | -0.66 (-0.88, -0.43) |
| Equatorial Guinea | 0.06 (0.01, 0.62) | 0.04 (0.01, 0.39) | 0.06 (0.01, 0.80) | 0.01 (0.00, 0.17) | -3.62 (-3.82, -3.42) |
| Eritrea | 0.21 (0.05, 1.83) | 0.02 (0.00, 0.16) | 0.27 (0.06, 2.89) | 0.01 (0.00, 0.11) | -2.09 (-2.16, -2.02) |
| Estonia | 7.43 (6.14, 9.21) | 0.59 (0.48, 0.73) | 5.09 (4.16, 6.18) | 0.43 (0.35, 0.53) | -2.07 (-2.78, -1.35) |
| Eswatini | 1.08 (0.35, 1.84) | 0.44 (0.15, 0.72) | 1.66 (0.36, 3.31) | 0.35 (0.08, 0.69) | -0.71 (-1.34, -0.07) |
| Ethiopia | 3.57 (1.10, 31.85) | 0.02 (0.01, 0.19) | 2.52 (0.61, 28.63) | 0.01 (0.00, 0.08) | -4.17 (-4.39, -3.95) |
| Fiji | 0.00 (0.00, 0.00) | 0.00 (0.00, 0.00) | 0.00 (0.00, 0.00) | 0.00 (0.00, 0.00) | -3.96 (-4.46, -3.45) |
| Finland | 18.60 (15.96, 21.66) | 0.47 (0.41, 0.55) | 10.81 (9.21, 12.60) | 0.22 (0.19, 0.26) | -2.68 (-2.88, -2.48) |
| France | 67.88 (57.70, 78.88) | 0.15 (0.13, 0.18) | 42.09 (34.25, 50.52) | 0.07 (0.06, 0.08) | -2.29 (-2.56, -2.01) |
| Gabon | 0.13 (0.03, 1.38) | 0.03 (0.01, 0.34) | 0.12 (0.03, 1.42) | 0.01 (0.00, 0.16) | -3.01 (-3.26, -2.77) |
| Gambia | 0.00 (0.00, 0.00) | 0.00 (0.00, 0.00) | 0.00 (0.00, 0.00) | 0.00 (0.00, 0.00) | -0.65 (-0.90, -0.40) |
| Georgia | 0.06 (0.05, 0.07) | 0.00 (0.00, 0.00) | 0.53 (0.43, 0.64) | 0.02 (0.01, 0.02) | 6.18 (3.78, 8.63) |
| Germany | 72.08 (60.68, 85.13) | 0.10 (0.09, 0.12) | 61.45 (50.74, 73.95) | 0.07 (0.06, 0.08) | -1.42 (-1.86, -0.98) |
| Ghana | 0.00 (0.00, 0.00) | 0.00 (0.00, 0.00) | 0.00 (0.00, 0.00) | 0.00 (0.00, 0.00) | 0.94 (0.50, 1.37) |
| Greece | 3.93 (3.32, 4.58) | 0.04 (0.04, 0.05) | 4.30 (3.55, 5.25) | 0.04 (0.03, 0.05) | -0.04 (-0.78, 0.69) |
| Greenland | 0.05 (0.03, 0.09) | 0.18 (0.11, 0.32) | 0.09 (0.04, 0.17) | 0.15 (0.08, 0.29) | -0.07 (-0.50, 0.36) |
| Grenada | 0.02 (0.02, 0.03) | 0.06 (0.04, 0.09) | 0.03 (0.02, 0.05) | 0.04 (0.03, 0.06) | -0.61 (-0.95, -0.27) |
| Guam | 0.00 (0.00, 0.00) | 0.00 (0.00, 0.00) | 0.00 (0.00, 0.00) | 0.00 (0.00, 0.00) | -4.19 (-6.72, -1.60) |
| Guatemala | 9.44 (7.75, 13.47) | 0.31 (0.26, 0.43) | 20.50 (15.92, 25.78) | 0.27 (0.21, 0.33) | -0.42 (-0.76, -0.09) |
| Guinea | 0.00 (0.00, 0.00) | 0.00 (0.00, 0.00) | 0.00 (0.00, 0.01) | 0.00 (0.00, 0.00) | -1.20 (-1.29, -1.10) |
| Guinea-Bissau | 0.00 (0.00, 0.00) | 0.00 (0.00, 0.00) | 0.00 (0.00, 0.00) | 0.00 (0.00, 0.00) | -1.50 (-1.64, -1.36) |
| Guyana | 0.02 (0.01, 0.03) | 0.01 (0.00, 0.01) | 0.34 (0.23, 0.49) | 0.07 (0.05, 0.10) | 5.70 (2.86, 8.62) |
| Haiti | 8.27 (3.42, 18.16) | 0.32 (0.13, 0.69) | 15.03 (5.95, 33.94) | 0.25 (0.10, 0.55) | -0.68 (-0.76, -0.60) |
| Honduras | 9.22 (4.97, 15.67) | 0.58 (0.32, 0.97) | 28.75 (15.48, 51.54) | 0.62 (0.34, 1.10) | 0.26 (0.14, 0.38) |
| Hungary | 26.43 (23.05, 30.11) | 0.30 (0.27, 0.35) | 10.21 (8.43, 12.21) | 0.11 (0.09, 0.13) | -3.66 (-4.14, -3.18) |
| Iceland | 0.23 (0.19, 0.27) | 0.14 (0.12, 0.17) | 0.18 (0.14, 0.22) | 0.06 (0.05, 0.07) | -2.78 (-3.08, -2.48) |
| India | 628.99 (390.92, 923.96) | 0.18 (0.11, 0.26) | 1028.40 (708.28, 1551.10) | 0.13 (0.09, 0.19) | -1.08 (-1.17, -1.00) |
| Indonesia | 26.61 (12.84, 42.93) | 0.03 (0.02, 0.05) | 63.96 (28.44, 99.38) | 0.03 (0.01, 0.05) | 0.21 (0.01, 0.41) |
| Iran (Islamic Republic of) | 6.68 (4.24, 9.85) | 0.03 (0.02, 0.04) | 16.04 (11.23, 22.14) | 0.03 (0.02, 0.04) | 0.47 (0.22, 0.72) |
| Iraq | 1.99 (0.89, 3.79) | 0.03 (0.01, 0.06) | 3.32 (1.54, 6.23) | 0.02 (0.01, 0.03) | -2.47 (-2.59, -2.35) |
| Ireland | 9.79 (8.39, 11.41) | 0.45 (0.38, 0.52) | 6.33 (5.31, 7.53) | 0.15 (0.13, 0.18) | -3.21 (-3.37, -3.05) |
| Israel | 1.55 (1.27, 1.89) | 0.06 (0.05, 0.07) | 2.49 (2.01, 3.02) | 0.04 (0.03, 0.05) | -1.70 (-2.10, -1.29) |
| Italy | 68.99 (64.64, 73.31) | 0.14 (0.13, 0.15) | 49.66 (46.02, 53.65) | 0.08 (0.07, 0.09) | -2.05 (-2.28, -1.83) |
| Jamaica | 0.80 (0.61, 1.09) | 0.08 (0.06, 0.11) | 2.11 (1.44, 3.00) | 0.11 (0.07, 0.15) | 0.63 (0.23, 1.04) |
| Japan | 331.62 (317.14, 344.40) | 0.30 (0.29, 0.31) | 106.41 (100.60, 111.94) | 0.09 (0.09, 0.10) | -4.75 (-5.27, -4.23) |
| Jordan | 0.32 (0.16, 0.57) | 0.03 (0.01, 0.05) | 1.32 (0.71, 2.22) | 0.02 (0.01, 0.03) | -0.14 (-0.53, 0.26) |
| Kazakhstan | 0.40 (0.32, 0.50) | 0.00 (0.00, 0.01) | 12.13 (9.60, 14.89) | 0.09 (0.07, 0.11) | 8.54 (4.31, 12.94) |
| Kenya | 0.71 (0.21, 7.08) | 0.01 (0.00, 0.10) | 1.41 (0.38, 14.59) | 0.01 (0.00, 0.07) | -1.10 (-1.26, -0.94) |
| Kiribati | 0.00 (0.00, 0.00) | 0.00 (0.00, 0.00) | 0.00 (0.00, 0.00) | 0.00 (0.00, 0.00) | -0.81 (-0.89, -0.73) |
| Kuwait | 0.12 (0.08, 0.16) | 0.02 (0.01, 0.02) | 1.78 (1.15, 2.94) | 0.06 (0.04, 0.09) | 2.47 (1.08, 3.87) |
| Kyrgyzstan | 2.65 (2.02, 3.18) | 0.12 (0.09, 0.15) | 7.13 (5.44, 9.19) | 0.18 (0.14, 0.23) | 2.43 (1.38, 3.49) |
| Lao People's Democratic Republic | 1.83 (0.92, 3.24) | 0.11 (0.06, 0.20) | 2.50 (1.19, 4.38) | 0.07 (0.03, 0.11) | -1.97 (-2.11, -1.83) |
| Latvia | 10.30 (8.40, 12.50) | 0.47 (0.38, 0.57) | 7.55 (6.08, 9.29) | 0.42 (0.33, 0.51) | -1.46 (-2.16, -0.76) |
| Lebanon | 1.03 (0.51, 1.88) | 0.06 (0.03, 0.11) | 0.83 (0.45, 1.36) | 0.02 (0.01, 0.04) | -2.84 (-2.99, -2.69) |
| Lesotho | 1.64 (0.68, 2.74) | 0.27 (0.12, 0.45) | 2.82 (0.76, 5.07) | 0.34 (0.09, 0.61) | 1.25 (0.74, 1.77) |
| Liberia | 0.00 (0.00, 0.00) | 0.00 (0.00, 0.00) | 0.00 (0.00, 0.00) | 0.00 (0.00, 0.00) | -1.49 (-1.64, -1.35) |
| Libya | 0.51 (0.23, 0.98) | 0.03 (0.02, 0.06) | 3.73 (1.77, 6.83) | 0.08 (0.04, 0.15) | 3.88 (3.60, 4.16) |
| Lithuania | 15.34 (12.63, 18.67) | 0.54 (0.45, 0.66) | 16.24 (13.20, 19.69) | 0.60 (0.48, 0.72) | -0.63 (-1.22, -0.02) |
| Luxembourg | 0.40 (0.33, 0.47) | 0.12 (0.10, 0.15) | 0.28 (0.23, 0.34) | 0.05 (0.04, 0.06) | -2.78 (-3.03, -2.53) |
| Madagascar | 0.56 (0.13, 5.45) | 0.01 (0.00, 0.13) | 0.77 (0.15, 8.66) | 0.01 (0.00, 0.08) | -2.30 (-2.41, -2.18) |
| Malawi | 0.50 (0.12, 4.98) | 0.02 (0.00, 0.16) | 0.61 (0.13, 6.81) | 0.01 (0.00, 0.11) | -1.93 (-2.19, -1.68) |
| Malaysia | 1.82 (0.90, 3.31) | 0.03 (0.01, 0.05) | 2.93 (1.55, 5.36) | 0.01 (0.01, 0.03) | -2.03 (-2.19, -1.87) |
| Maldives | 0.13 (0.06, 0.25) | 0.15 (0.08, 0.30) | 0.12 (0.07, 0.21) | 0.04 (0.02, 0.07) | -4.41 (-4.55, -4.26) |
| Mali | 0.00 (0.00, 0.01) | 0.00 (0.00, 0.00) | 0.00 (0.00, 0.01) | 0.00 (0.00, 0.00) | -1.26 (-1.32, -1.20) |
| Malta | 0.30 (0.25, 0.36) | 0.12 (0.10, 0.14) | 0.26 (0.21, 0.31) | 0.06 (0.05, 0.07) | -1.95 (-2.27, -1.62) |
| Marshall Islands | 0.00 (0.00, 0.00) | 0.00 (0.00, 0.00) | 0.00 (0.00, 0.00) | 0.00 (0.00, 0.00) | 1.07 (0.76, 1.39) |
| Mauritania | 0.00 (0.00, 0.00) | 0.00 (0.00, 0.00) | 0.00 (0.00, 0.00) | 0.00 (0.00, 0.00) | -2.27 (-2.34, -2.20) |
| Mauritius | 0.02 (0.02, 0.03) | 0.00 (0.00, 0.01) | 1.24 (0.98, 1.57) | 0.10 (0.08, 0.13) | 5.78 (2.59, 9.06) |
| Mexico | 217.54 (206.76, 228.43) | 0.66 (0.63, 0.69) | 521.83 (435.78, 622.92) | 0.60 (0.50, 0.71) | -0.27 (-0.46, -0.08) |
| Micronesia (Federated States of) | 0.00 (0.00, 0.00) | 0.00 (0.00, 0.00) | 0.00 (0.00, 0.00) | 0.00 (0.00, 0.00) | 0.49 (0.38, 0.61) |
| Monaco | 0.00 (0.00, 0.00) | 0.01 (0.00, 0.01) | 0.00 (0.00, 0.00) | 0.01 (0.00, 0.01) | -1.01 (-1.09, -0.94) |
| Mongolia | 0.69 (0.36, 2.10) | 0.09 (0.05, 0.27) | 2.95 (1.72, 4.66) | 0.14 (0.08, 0.23) | 2.25 (1.57, 2.94) |
| Montenegro | 0.38 (0.25, 0.54) | 0.09 (0.06, 0.12) | 0.36 (0.23, 0.53) | 0.06 (0.04, 0.09) | -0.95 (-1.21, -0.70) |
| Morocco | 6.28 (2.04, 15.59) | 0.06 (0.02, 0.14) | 22.83 (7.39, 52.27) | 0.09 (0.03, 0.21) | 2.06 (1.69, 2.44) |
| Mozambique | 0.81 (0.17, 8.38) | 0.02 (0.00, 0.17) | 1.16 (0.22, 12.34) | 0.01 (0.00, 0.12) | -0.91 (-1.02, -0.80) |
| Myanmar | 18.22 (8.95, 31.83) | 0.10 (0.05, 0.17) | 20.88 (10.74, 35.23) | 0.06 (0.03, 0.10) | -2.02 (-2.22, -1.81) |
| Namibia | 1.58 (0.68, 2.75) | 0.31 (0.14, 0.53) | 2.38 (0.64, 4.85) | 0.21 (0.06, 0.43) | -1.65 (-2.02, -1.28) |
| Nauru | 0.00 (0.00, 0.00) | 0.00 (0.00, 0.00) | 0.00 (0.00, 0.00) | 0.00 (0.00, 0.00) | 1.11 (0.60, 1.61) |
| Nepal | 11.41 (5.19, 19.81) | 0.16 (0.07, 0.28) | 20.60 (10.26, 37.05) | 0.13 (0.06, 0.23) | -0.58 (-0.90, -0.26) |
| Netherlands | 33.50 (28.52, 38.70) | 0.31 (0.26, 0.36) | 22.49 (18.82, 26.85) | 0.13 (0.11, 0.16) | -2.53 (-2.95, -2.10) |
| New Zealand | 7.36 (6.33, 8.60) | 0.33 (0.29, 0.39) | 6.08 (5.03, 7.21) | 0.13 (0.11, 0.16) | -2.92 (-3.34, -2.49) |
| Nicaragua | 3.24 (2.04, 4.97) | 0.27 (0.17, 0.40) | 7.19 (4.54, 10.90) | 0.20 (0.13, 0.31) | -0.67 (-0.82, -0.52) |
| Niger | 0.00 (0.00, 0.00) | 0.00 (0.00, 0.00) | 0.00 (0.00, 0.01) | 0.00 (0.00, 0.00) | -1.42 (-1.56, -1.28) |
| Nigeria | 0.01 (0.00, 0.04) | 0.00 (0.00, 0.00) | 0.01 (0.00, 0.09) | 0.00 (0.00, 0.00) | -0.76 (-0.88, -0.64) |
| Niue | 0.00 (0.00, 0.00) | 0.00 (0.00, 0.00) | 0.00 (0.00, 0.00) | 0.00 (0.00, 0.00) | 0.57 (0.47, 0.67) |
| North Macedonia | 0.41 (0.26, 0.64) | 0.03 (0.02, 0.05) | 0.26 (0.15, 0.43) | 0.01 (0.01, 0.02) | -3.28 (-3.54, -3.03) |
| Northern Mariana Islands | 0.00 (0.00, 0.00) | 0.01 (0.01, 0.02) | 0.00 (0.00, 0.01) | 0.01 (0.01, 0.02) | -0.16 (-1.31, 1.01) |
| Norway | 11.59 (10.84, 12.42) | 0.39 (0.36, 0.42) | 4.02 (3.65, 4.41) | 0.08 (0.08, 0.09) | -5.32 (-5.74, -4.90) |
| Oman | 0.13 (0.06, 0.26) | 0.02 (0.01, 0.04) | 0.39 (0.20, 0.69) | 0.02 (0.01, 0.03) | 0.43 (-0.09, 0.96) |
| Pakistan | 69.05 (34.10, 105.79) | 0.18 (0.09, 0.28) | 163.85 (84.19, 269.49) | 0.18 (0.09, 0.29) | -0.40 (-0.67, -0.13) |
| Palau | 0.00 (0.00, 0.00) | 0.00 (0.00, 0.00) | 0.00 (0.00, 0.00) | 0.00 (0.00, 0.00) | -2.65 (-2.78, -2.52) |
| Palestine | 0.35 (0.17, 0.60) | 0.05 (0.03, 0.09) | 0.62 (0.35, 1.01) | 0.03 (0.02, 0.05) | -2.15 (-2.25, -2.05) |
| Panama | 1.60 (1.27, 1.98) | 0.16 (0.12, 0.19) | 3.98 (2.88, 5.21) | 0.14 (0.10, 0.19) | -0.51 (-0.73, -0.29) |
| Papua New Guinea | 0.02 (0.00, 0.04) | 0.00 (0.00, 0.00) | 0.05 (0.01, 0.14) | 0.00 (0.00, 0.00) | 0.41 (0.35, 0.47) |
| Paraguay | 3.15 (1.96, 4.95) | 0.19 (0.12, 0.29) | 14.38 (8.61, 21.94) | 0.35 (0.21, 0.53) | 2.52 (2.14, 2.90) |
| Peru | 14.53 (9.28, 21.54) | 0.16 (0.10, 0.24) | 25.42 (14.41, 41.42) | 0.11 (0.07, 0.19) | -1.77 (-2.20, -1.35) |
| Philippines | 22.82 (14.92, 30.39) | 0.09 (0.06, 0.12) | 44.32 (29.64, 60.31) | 0.07 (0.05, 0.09) | -1.10 (-1.31, -0.88) |
| Poland | 182.80 (174.88, 190.91) | 0.66 (0.63, 0.68) | 52.61 (47.45, 57.89) | 0.15 (0.13, 0.16) | -4.85 (-5.33, -4.37) |
| Portugal | 14.77 (12.50, 17.28) | 0.18 (0.16, 0.22) | 8.63 (7.19, 10.19) | 0.08 (0.07, 0.10) | -2.49 (-2.83, -2.15) |
| Puerto Rico | 2.33 (1.93, 2.77) | 0.11 (0.09, 0.13) | 3.28 (2.46, 4.28) | 0.11 (0.08, 0.14) | -0.49 (-1.00, 0.02) |
| Qatar | 0.03 (0.02, 0.06) | 0.02 (0.01, 0.04) | 0.19 (0.10, 0.33) | 0.01 (0.01, 0.02) | -1.06 (-1.38, -0.74) |
| Republic of Korea | 55.16 (37.61, 78.58) | 0.24 (0.17, 0.34) | 29.70 (18.54, 46.91) | 0.05 (0.03, 0.08) | -5.61 (-5.90, -5.31) |
| Republic of Moldova | 3.34 (2.75, 4.01) | 0.11 (0.09, 0.13) | 5.35 (4.28, 6.58) | 0.15 (0.12, 0.19) | -0.22 (-0.79, 0.35) |
| Romania | 0.22 (0.17, 0.30) | 0.00 (0.00, 0.00) | 0.16 (0.12, 0.20) | 0.00 (0.00, 0.00) | -1.32 (-1.53, -1.11) |
| Russian Federation | 424.95 (411.08, 439.45) | 0.35 (0.34, 0.36) | 362.38 (325.26, 398.00) | 0.26 (0.24, 0.29) | -1.55 (-1.96, -1.15) |
| Rwanda | 0.54 (0.14, 5.21) | 0.02 (0.01, 0.22) | 0.46 (0.09, 5.23) | 0.01 (0.00, 0.10) | -4.25 (-4.65, -3.85) |
| Saint Kitts and Nevis | 0.05 (0.03, 0.06) | 0.26 (0.17, 0.34) | 0.09 (0.06, 0.13) | 0.17 (0.11, 0.24) | -1.60 (-1.87, -1.33) |
| Saint Lucia | 0.10 (0.08, 0.12) | 0.18 (0.14, 0.23) | 0.23 (0.17, 0.31) | 0.15 (0.11, 0.20) | -1.10 (-1.43, -0.76) |
| Saint Vincent and the Grenadines | 0.04 (0.03, 0.04) | 0.08 (0.07, 0.10) | 0.05 (0.04, 0.07) | 0.06 (0.04, 0.08) | -1.16 (-1.79, -0.53) |
| Samoa | 0.00 (0.00, 0.00) | 0.00 (0.00, 0.00) | 0.00 (0.00, 0.00) | 0.00 (0.00, 0.00) | 0.55 (0.50, 0.60) |
| San Marino | 0.01 (0.00, 0.01) | 0.04 (0.02, 0.06) | 0.01 (0.00, 0.01) | 0.02 (0.01, 0.04) | -1.42 (-1.71, -1.12) |
| Sao Tome and Principe | 0.00 (0.00, 0.00) | 0.00 (0.00, 0.00) | 0.00 (0.00, 0.00) | 0.00 (0.00, 0.00) | -1.73 (-1.95, -1.50) |
| Saudi Arabia | 1.71 (0.76, 3.44) | 0.03 (0.01, 0.06) | 8.71 (3.82, 18.08) | 0.04 (0.02, 0.08) | 0.97 (0.71, 1.22) |
| Senegal | 0.00 (0.00, 0.00) | 0.00 (0.00, 0.00) | 0.00 (0.00, 0.01) | 0.00 (0.00, 0.00) | -1.14 (-1.25, -1.03) |
| Serbia | 10.78 (6.81, 16.55) | 0.13 (0.08, 0.20) | 6.09 (3.85, 9.16) | 0.07 (0.04, 0.11) | -1.95 (-2.08, -1.82) |
| Seychelles | 0.00 (0.00, 0.01) | 0.01 (0.01, 0.02) | 0.01 (0.00, 0.01) | 0.01 (0.00, 0.01) | -1.30 (-1.44, -1.16) |
| Sierra Leone | 0.00 (0.00, 0.00) | 0.00 (0.00, 0.00) | 0.00 (0.00, 0.00) | 0.00 (0.00, 0.00) | -0.74 (-0.87, -0.61) |
| Singapore | 0.77 (0.61, 0.95) | 0.05 (0.04, 0.06) | 0.62 (0.49, 0.76) | 0.01 (0.01, 0.01) | -5.52 (-6.04, -4.99) |
| Slovakia | 4.56 (3.04, 6.49) | 0.12 (0.08, 0.18) | 2.94 (1.82, 4.47) | 0.06 (0.03, 0.09) | -2.66 (-2.85, -2.46) |
| Slovenia | 5.84 (4.97, 6.78) | 0.37 (0.31, 0.43) | 2.04 (1.61, 2.52) | 0.09 (0.07, 0.12) | -4.70 (-5.19, -4.21) |
| Solomon Islands | 0.00 (0.00, 0.00) | 0.00 (0.00, 0.00) | 0.01 (0.00, 0.01) | 0.00 (0.00, 0.00) | 1.12 (0.99, 1.25) |
| Somalia | 0.64 (0.16, 5.15) | 0.03 (0.01, 0.21) | 0.92 (0.19, 8.96) | 0.02 (0.00, 0.15) | -1.96 (-2.09, -1.83) |
| South Africa | 112.73 (81.37, 144.39) | 0.67 (0.48, 0.86) | 135.34 (103.33, 179.83) | 0.39 (0.30, 0.52) | -2.32 (-2.74, -1.90) |
| South Sudan | 0.27 (0.07, 2.33) | 0.01 (0.00, 0.12) | 0.36 (0.08, 3.78) | 0.01 (0.00, 0.11) | -1.42 (-1.64, -1.19) |
| Spain | 45.40 (38.15, 53.84) | 0.15 (0.12, 0.17) | 26.90 (21.47, 33.07) | 0.06 (0.05, 0.07) | -3.01 (-3.34, -2.67) |
| Sri Lanka | 1.08 (0.64, 1.78) | 0.01 (0.01, 0.02) | 1.43 (0.73, 2.44) | 0.01 (0.00, 0.01) | -1.04 (-1.42, -0.66) |
| Sudan | 4.20 (1.49, 9.31) | 0.06 (0.02, 0.13) | 12.60 (5.24, 25.79) | 0.07 (0.03, 0.14) | 1.06 (0.83, 1.30) |
| Suriname | 0.09 (0.05, 0.16) | 0.05 (0.03, 0.08) | 0.16 (0.08, 0.27) | 0.04 (0.02, 0.06) | -1.16 (-1.37, -0.95) |
| Sweden | 14.89 (12.62, 17.33) | 0.23 (0.19, 0.26) | 6.23 (5.00, 7.63) | 0.07 (0.06, 0.08) | -3.96 (-4.21, -3.71) |
| Switzerland | 7.52 (6.12, 9.12) | 0.14 (0.11, 0.17) | 5.19 (4.25, 6.30) | 0.06 (0.05, 0.07) | -2.89 (-3.06, -2.73) |
| Syrian Arab Republic | 2.19 (1.33, 3.39) | 0.05 (0.03, 0.07) | 4.98 (2.78, 7.62) | 0.05 (0.03, 0.07) | 0.55 (0.31, 0.80) |
| Taiwan (Province of China) | 25.70 (22.04, 29.92) | 0.21 (0.18, 0.25) | 26.70 (21.34, 32.97) | 0.11 (0.09, 0.13) | -1.26 (-1.82, -0.70) |
| Tajikistan | 0.30 (0.16, 0.64) | 0.01 (0.01, 0.03) | 2.21 (0.75, 4.83) | 0.04 (0.01, 0.09) | 3.73 (3.14, 4.32) |
| Thailand | 37.03 (19.34, 68.67) | 0.13 (0.07, 0.24) | 52.29 (29.34, 87.83) | 0.08 (0.04, 0.13) | -2.08 (-2.23, -1.92) |
| Timor-Leste | 0.18 (0.08, 0.32) | 0.07 (0.03, 0.12) | 0.35 (0.16, 0.62) | 0.06 (0.03, 0.10) | -0.26 (-0.46, -0.05) |
| Togo | 0.00 (0.00, 0.00) | 0.00 (0.00, 0.00) | 0.00 (0.00, 0.00) | 0.00 (0.00, 0.00) | -1.03 (-1.14, -0.91) |
| Tokelau | 0.00 (0.00, 0.00) | 0.00 (0.00, 0.00) | 0.00 (0.00, 0.00) | 0.00 (0.00, 0.00) | -0.19 (-0.32, -0.06) |
| Tonga | 0.00 (0.00, 0.00) | 0.00 (0.00, 0.00) | 0.00 (0.00, 0.00) | 0.00 (0.00, 0.00) | -0.04 (-0.15, 0.06) |
| Trinidad and Tobago | 2.04 (1.63, 2.52) | 0.37 (0.30, 0.46) | 3.43 (2.38, 4.78) | 0.29 (0.20, 0.41) | -1.38 (-1.76, -0.99) |
| Tunisia | 0.96 (0.46, 1.81) | 0.02 (0.01, 0.05) | 3.04 (1.37, 5.66) | 0.03 (0.02, 0.06) | 1.25 (0.97, 1.53) |
| Turkiye | 80.07 (46.10, 129.23) | 0.28 (0.16, 0.45) | 76.10 (46.08, 118.79) | 0.12 (0.07, 0.19) | -2.78 (-3.05, -2.51) |
| Turkmenistan | 0.01 (0.01, 0.01) | 0.00 (0.00, 0.00) | 1.12 (0.76, 1.59) | 0.03 (0.02, 0.05) | 10.37 (8.60, 12.17) |
| Tuvalu | 0.00 (0.00, 0.00) | 0.00 (0.00, 0.00) | 0.00 (0.00, 0.00) | 0.00 (0.00, 0.00) | -0.16 (-0.20, -0.12) |
| Uganda | 0.59 (0.15, 4.97) | 0.01 (0.00, 0.10) | 0.99 (0.20, 10.95) | 0.01 (0.00, 0.08) | -2.17 (-2.58, -1.76) |
| Ukraine | 75.97 (54.54, 95.62) | 0.17 (0.13, 0.22) | 76.43 (51.73, 107.83) | 0.19 (0.13, 0.26) | -0.35 (-0.74, 0.05) |
| United Arab Emirates | 0.64 (0.32, 1.13) | 0.11 (0.06, 0.18) | 2.52 (1.30, 4.37) | 0.04 (0.02, 0.06) | -2.86 (-3.48, -2.23) |
| United Kingdom | 210.79 (204.80, 216.67) | 0.47 (0.46, 0.48) | 100.74 (96.66, 105.19) | 0.16 (0.16, 0.17) | -2.48 (-3.10, -1.85) |
| United Republic of Tanzania | 1.28 (0.31, 12.33) | 0.02 (0.00, 0.14) | 1.66 (0.31, 18.91) | 0.01 (0.00, 0.09) | -2.40 (-2.51, -2.28) |
| United States of America | 313.14 (300.26, 325.82) | 0.19 (0.18, 0.20) | 356.26 (331.89, 400.91) | 0.12 (0.11, 0.13) | -1.59 (-1.91, -1.26) |
| United States Virgin Islands | 0.04 (0.02, 0.07) | 0.06 (0.03, 0.11) | 0.03 (0.02, 0.06) | 0.04 (0.02, 0.07) | -1.42 (-1.61, -1.23) |
| Uruguay | 6.73 (5.65, 8.07) | 0.31 (0.26, 0.37) | 9.07 (7.48, 10.68) | 0.34 (0.28, 0.40) | 0.45 (0.21, 0.69) |
| Uzbekistan | 0.52 (0.39, 0.87) | 0.01 (0.00, 0.01) | 26.49 (20.80, 33.07) | 0.12 (0.09, 0.15) | 7.15 (5.13, 9.21) |
| Vanuatu | 0.00 (0.00, 0.00) | 0.00 (0.00, 0.00) | 0.00 (0.00, 0.00) | 0.00 (0.00, 0.00) | 0.67 (0.54, 0.81) |
| Venezuela (Bolivarian Republic of) | 30.38 (25.14, 36.56) | 0.41 (0.34, 0.50) | 75.96 (51.87, 108.83) | 0.38 (0.26, 0.54) | -0.82 (-1.11, -0.54) |
| Viet Nam | 17.66 (8.64, 30.09) | 0.06 (0.03, 0.10) | 35.01 (16.04, 61.66) | 0.05 (0.02, 0.08) | -0.74 (-0.80, -0.68) |
| Yemen | 1.74 (0.78, 3.53) | 0.04 (0.02, 0.09) | 8.17 (3.73, 15.53) | 0.07 (0.03, 0.13) | 1.78 (1.50, 2.06) |
| Zambia | 0.43 (0.10, 4.53) | 0.02 (0.00, 0.18) | 0.50 (0.11, 4.98) | 0.01 (0.00, 0.08) | -3.12 (-3.46, -2.78) |
| Zimbabwe | 6.98 (3.30, 11.45) | 0.21 (0.10, 0.34) | 16.56 (6.73, 29.00) | 0.27 (0.11, 0.47) | 1.34 (0.74, 1.95) |
