# Supplementary material for: Global burden and regional disparities of rheumatoid arthritis among the working-age population: A comprehensive analysis from 1990 to 2021 with projections to 2040
Source: PLoS One. 2025 Jun 4;20(6):e0325127. doi: 10.1371/journal.pone.0325127 (PMC12136291; doi:10.1371/journal.pone.0325127)
Supplement: S4 Table — (DOCX) [file pone.0325127.s019.docx]

**S4 Table.** National trends in the burden of rheumatoid arthritis DALYs among working-age population: 1990−2021

| **Location** | **1990** | | **2021** | | **EAPC (95% CI)** |
| --- | --- | --- | --- | --- | --- |
|  | **Number** | **ASR** | **Number** | **ASR** |  |
| Afghanistan | 767.87 (413.47, 1274.16) | 16.26 (8.72, 26.87) | 2610.17 (1450.33, 4203.77) | 21.11 (11.92, 33.99) | 1.07 (0.99, 1.16) |
| Albania | 355.49 (220.18, 543.64) | 20.43 (12.85, 30.93) | 579.10 (357.75, 874.74) | 27.34 (16.46, 41.91) | 1.16 (1.08, 1.23) |
| Algeria | 1137.99 (624.06, 1828.51) | 10.36 (5.76, 16.55) | 4451.15 (2600.69, 7000.31) | 15.64 (9.18, 24.53) | 1.49 (1.41, 1.58) |
| American Samoa | 3.04 (1.63, 5.06) | 12.45 (6.75, 20.53) | 5.00 (2.73, 8.09) | 15.23 (8.28, 24.83) | 0.57 (0.50, 0.64) |
| Andorra | 10.00 (5.67, 15.82) | 25.41 (14.44, 40.05) | 21.72 (12.78, 33.85) | 28.43 (16.33, 44.94) | 0.48 (0.43, 0.53) |
| Angola | 581.71 (318.41, 1054.98) | 14.43 (8.07, 26.21) | 2230.58 (1210.56, 3674.19) | 17.65 (9.83, 28.68) | 0.76 (0.68, 0.84) |
| Antigua and Barbuda | 6.19 (3.77, 9.65) | 19.52 (12.16, 29.95) | 18.13 (11.42, 27.28) | 25.77 (16.05, 39.12) | 0.84 (0.74, 0.94) |
| Argentina | 6419.35 (4310.17, 9192.66) | 32.09 (21.50, 46.02) | 13292.35 (8751.84, 19506.97) | 42.68 (28.01, 62.76) | 0.99 (0.83, 1.14) |
| Armenia | 284.83 (152.55, 470.20) | 13.40 (7.17, 22.09) | 481.48 (287.16, 749.40) | 21.06 (12.41, 33.03) | 1.71 (1.58, 1.84) |
| Australia | 4960.36 (3279.76, 7222.34) | 44.02 (29.09, 64.07) | 9473.22 (5936.05, 14110.72) | 47.37 (29.13, 71.47) | 0.34 (0.24, 0.45) |
| Austria | 2468.25 (1596.37, 3607.90) | 43.30 (27.70, 63.81) | 3236.43 (2018.06, 4918.20) | 43.22 (26.26, 66.81) | 0.10 (0.00, 0.21) |
| Azerbaijan | 493.60 (265.14, 811.88) | 11.94 (6.42, 19.65) | 1227.77 (667.71, 1996.68) | 15.36 (8.31, 25.08) | 1.09 (0.82, 1.35) |
| Bahamas | 33.78 (21.49, 50.38) | 25.46 (16.68, 37.13) | 84.07 (53.49, 124.60) | 28.88 (18.22, 43.09) | 0.41 (0.34, 0.48) |
| Bahrain | 57.50 (31.35, 93.41) | 21.25 (12.19, 33.28) | 422.61 (257.96, 645.70) | 36.09 (22.36, 54.61) | 1.81 (1.75, 1.86) |
| Bangladesh | 8803.76 (5261.56, 13624.43) | 20.76 (12.78, 31.31) | 26746.34 (15963.81, 41279.28) | 26.44 (15.94, 40.50) | 0.96 (0.78, 1.13) |
| Barbados | 44.44 (28.25, 66.39) | 30.43 (19.62, 44.88) | 98.46 (63.77, 142.51) | 39.62 (25.00, 58.44) | 0.80 (0.70, 0.91) |
| Belarus | 1115.32 (621.97, 1788.56) | 14.75 (8.16, 23.78) | 1720.04 (1001.44, 2702.16) | 21.85 (12.44, 34.82) | 1.32 (1.19, 1.46) |
| Belgium | 2871.78 (1821.36, 4271.87) | 38.08 (23.81, 57.29) | 3746.08 (2336.81, 5671.73) | 41.18 (25.13, 63.56) | 0.29 (0.26, 0.32) |
| Belize | 16.59 (10.28, 25.44) | 22.36 (14.26, 33.47) | 80.82 (51.72, 120.59) | 31.82 (20.70, 46.93) | 1.07 (0.95, 1.18) |
| Benin | 151.35 (77.93, 255.20) | 8.40 (4.37, 14.05) | 594.05 (310.09, 993.38) | 10.65 (5.61, 17.64) | 0.82 (0.73, 0.91) |
| Bermuda | 9.78 (5.82, 15.11) | 22.89 (13.71, 35.18) | 15.39 (9.44, 23.79) | 28.47 (16.90, 45.12) | 0.73 (0.68, 0.78) |
| Bhutan | 63.51 (37.57, 99.06) | 25.55 (15.56, 38.94) | 158.39 (94.55, 244.51) | 33.69 (20.48, 51.37) | 0.96 (0.84, 1.09) |
| Bolivia (Plurinational State of) | 1187.40 (774.88, 1731.90) | 42.26 (28.24, 60.66) | 3916.42 (2510.90, 5767.25) | 55.98 (36.23, 81.74) | 0.95 (0.92, 0.97) |
| Bosnia and Herzegovina | 1011.91 (647.76, 1500.37) | 31.84 (20.27, 47.37) | 1183.90 (755.72, 1761.57) | 41.81 (25.98, 63.21) | 1.09 (1.02, 1.16) |
| Botswana | 181.17 (115.84, 267.38) | 35.52 (23.11, 51.69) | 528.88 (331.13, 791.08) | 36.85 (23.50, 54.45) | 0.15 (-0.01, 0.30) |
| Brazil | 28686.38 (19618.88, 40951.74) | 35.78 (24.82, 50.56) | 55070.80 (37814.43, 77330.27) | 34.92 (23.88, 49.16) | 0.19 (0.10, 0.28) |
| Brunei Darussalam | 42.72 (27.23, 63.99) | 40.23 (26.65, 58.09) | 136.18 (87.06, 201.87) | 42.70 (27.57, 62.78) | 0.29 (0.25, 0.32) |
| Bulgaria | 1268.49 (774.57, 1964.26) | 19.07 (11.42, 29.96) | 1196.02 (711.64, 1879.80) | 21.93 (12.75, 35.05) | 0.42 (0.23, 0.60) |
| Burkina Faso | 293.66 (154.79, 488.58) | 7.92 (4.21, 13.10) | 914.50 (469.49, 1532.48) | 9.76 (5.06, 16.25) | 0.77 (0.69, 0.84) |
| Burundi | 240.65 (131.62, 410.03) | 11.87 (6.64, 20.14) | 636.64 (342.03, 1058.96) | 12.26 (6.72, 20.09) | 0.16 (0.11, 0.20) |
| Cabo Verde | 12.44 (6.27, 21.11) | 8.91 (4.49, 15.02) | 42.09 (22.57, 69.49) | 11.55 (6.22, 19.03) | 0.96 (0.85, 1.08) |
| Cambodia | 504.42 (312.25, 763.67) | 12.02 (7.54, 17.97) | 1473.07 (902.69, 2253.86) | 14.44 (8.90, 21.97) | 0.58 (0.54, 0.62) |
| Cameroon | 426.63 (224.72, 712.57) | 9.87 (5.25, 16.34) | 1685.31 (890.82, 2804.22) | 11.94 (6.34, 19.70) | 0.64 (0.47, 0.81) |
| Canada | 7663.87 (5243.14, 10805.11) | 40.97 (28.07, 57.68) | 15719.65 (10627.59, 22121.45) | 50.90 (33.90, 72.38) | 0.68 (0.57, 0.79) |
| Central African Republic | 178.28 (99.68, 332.34) | 16.17 (9.21, 30.23) | 394.86 (221.57, 662.19) | 16.36 (9.35, 27.26) | 0.07 (0.03, 0.10) |
| Chad | 166.82 (88.00, 280.80) | 7.24 (3.85, 12.07) | 519.43 (270.90, 862.22) | 8.17 (4.30, 13.39) | 0.43 (0.30, 0.57) |
| Chile | 3535.25 (2490.37, 4947.04) | 48.10 (34.36, 66.55) | 8992.86 (5819.63, 13165.58) | 62.26 (39.75, 92.13) | 0.99 (0.89, 1.10) |
| China | 292715.93 (208270.17, 407117.50) | 42.86 (30.73, 59.24) | 512147.29 (353836.57, 722685.52) | 43.11 (29.41, 61.50) | 0.17 (0.08, 0.27) |
| Colombia | 5924.67 (4094.51, 8461.22) | 38.86 (27.48, 54.44) | 16574.88 (10934.86, 23888.22) | 48.08 (31.59, 69.48) | 0.84 (0.72, 0.96) |
| Comoros | 22.25 (12.05, 37.43) | 12.44 (6.83, 20.67) | 58.25 (31.86, 95.70) | 14.12 (7.79, 23.03) | 0.48 (0.40, 0.55) |
| Congo | 183.18 (101.60, 330.28) | 19.62 (11.09, 35.43) | 646.32 (367.12, 1039.08) | 23.27 (13.52, 37.00) | 0.66 (0.58, 0.74) |
| Cook Islands | 0.98 (0.53, 1.61) | 9.40 (5.09, 15.47) | 1.67 (0.92, 2.74) | 13.31 (7.22, 22.12) | 1.14 (1.08, 1.19) |
| Costa Rica | 587.71 (380.16, 874.64) | 43.11 (28.72, 62.69) | 2254.30 (1498.84, 3254.82) | 65.39 (43.24, 94.77) | 1.24 (1.16, 1.32) |
| Cote d'Ivoire | 409.49 (214.00, 690.89) | 8.38 (4.46, 13.99) | 1361.61 (708.42, 2232.44) | 10.53 (5.57, 17.13) | 0.77 (0.64, 0.91) |
| Croatia | 1331.85 (889.94, 1954.01) | 33.78 (22.13, 50.35) | 1559.86 (978.17, 2329.82) | 43.24 (26.38, 65.82) | 0.82 (0.74, 0.90) |
| Cuba | 2022.99 (1299.94, 3014.21) | 30.42 (19.77, 44.85) | 3821.21 (2536.54, 5593.54) | 39.45 (25.43, 59.04) | 0.80 (0.75, 0.84) |
| Cyprus | 248.66 (163.09, 360.75) | 50.64 (33.33, 73.21) | 580.43 (363.60, 862.52) | 53.05 (32.99, 79.26) | 0.33 (0.22, 0.44) |
| Czechia | 2005.40 (1268.52, 2998.05) | 27.14 (16.98, 40.94) | 2738.76 (1679.69, 4189.25) | 33.07 (19.82, 51.32) | 0.66 (0.59, 0.72) |
| Democratic People's Republic of Korea | 4632.08 (3054.27, 6665.81) | 35.53 (23.46, 51.11) | 9439.05 (6348.53, 13425.57) | 43.15 (28.74, 61.85) | 0.67 (0.63, 0.71) |
| Democratic Republic of the Congo | 2075.42 (1119.96, 3894.56) | 14.04 (7.71, 26.52) | 5913.64 (3264.72, 9744.32) | 15.59 (8.77, 25.50) | 0.32 (0.21, 0.43) |
| Denmark | 1460.54 (979.53, 2091.27) | 38.73 (25.82, 55.75) | 2172.12 (1379.49, 3177.65) | 46.16 (28.65, 68.81) | 0.63 (0.58, 0.68) |
| Djibouti | 18.36 (9.67, 30.73) | 11.29 (6.09, 18.70) | 95.76 (51.16, 159.82) | 13.80 (7.52, 22.79) | 0.69 (0.58, 0.80) |
| Dominica | 7.89 (4.99, 11.94) | 22.22 (14.23, 33.32) | 12.51 (7.94, 18.40) | 25.24 (15.80, 37.49) | 0.34 (0.31, 0.38) |
| Dominican Republic | 557.21 (354.01, 832.31) | 16.69 (10.78, 24.61) | 1267.66 (773.26, 1943.43) | 18.10 (11.09, 27.66) | 0.57 (0.43, 0.71) |
| Ecuador | 1917.00 (1317.01, 2741.92) | 41.92 (29.29, 59.03) | 6081.07 (3877.23, 9013.92) | 54.98 (35.26, 81.14) | 0.75 (0.62, 0.88) |
| Egypt | 3816.56 (2133.22, 6111.20) | 13.85 (7.92, 21.89) | 12984.73 (7462.00, 20394.32) | 21.26 (12.33, 33.19) | 1.36 (1.31, 1.41) |
| El Salvador | 463.94 (281.26, 716.73) | 19.88 (12.27, 30.37) | 1085.70 (656.78, 1685.81) | 28.24 (17.17, 43.71) | 1.22 (1.15, 1.30) |
| Equatorial Guinea | 25.61 (13.90, 51.49) | 14.95 (8.21, 30.24) | 140.34 (78.71, 230.17) | 23.83 (13.97, 38.00) | 1.92 (1.74, 2.10) |
| Eritrea | 151.61 (82.39, 257.00) | 11.80 (6.54, 19.86) | 391.56 (210.65, 641.14) | 13.10 (7.25, 21.12) | 0.31 (0.28, 0.33) |
| Estonia | 819.35 (593.79, 1110.06) | 68.58 (49.20, 93.92) | 799.23 (567.11, 1103.01) | 74.55 (52.05, 104.55) | 0.25 (0.14, 0.36) |
| Eswatini | 134.41 (84.65, 194.60) | 46.62 (29.96, 66.37) | 258.55 (159.55, 385.86) | 46.33 (28.88, 68.11) | -0.08 (-0.25, 0.09) |
| Ethiopia | 2498.13 (1469.88, 4056.45) | 13.34 (8.00, 21.44) | 5404.50 (3191.28, 8551.47) | 12.14 (7.35, 18.85) | -0.18 (-0.26, -0.10) |
| Fiji | 31.02 (16.49, 50.71) | 7.69 (4.13, 12.47) | 57.52 (31.71, 93.32) | 9.59 (5.29, 15.57) | 0.71 (0.60, 0.82) |
| Finland | 2444.99 (1749.77, 3369.62) | 64.68 (46.05, 89.60) | 3014.25 (2030.08, 4345.40) | 68.31 (45.14, 100.22) | 0.30 (0.24, 0.37) |
| France | 12159.48 (7989.58, 17932.79) | 29.45 (19.15, 43.67) | 16390.38 (10138.96, 24587.71) | 32.09 (19.41, 49.08) | 0.44 (0.38, 0.51) |
| Gabon | 80.42 (45.51, 138.85) | 19.00 (10.83, 32.65) | 242.74 (137.89, 385.77) | 25.87 (14.91, 40.73) | 1.07 (1.01, 1.13) |
| Gambia | 30.96 (15.99, 52.56) | 8.13 (4.25, 13.69) | 106.88 (54.73, 179.12) | 10.32 (5.38, 17.09) | 0.88 (0.79, 0.96) |
| Georgia | 553.55 (296.46, 915.26) | 14.54 (7.72, 24.17) | 474.56 (270.57, 757.79) | 17.69 (9.90, 28.61) | 0.72 (0.62, 0.81) |
| Germany | 21259.99 (13330.16, 32053.36) | 34.03 (21.00, 51.82) | 27134.54 (16948.75, 41087.46) | 38.62 (23.37, 59.74) | 0.45 (0.39, 0.51) |
| Ghana | 494.13 (257.13, 820.55) | 7.80 (4.12, 12.85) | 1912.45 (991.80, 3184.89) | 11.13 (5.81, 18.37) | 1.21 (1.10, 1.33) |
| Greece | 2555.27 (1563.78, 3812.41) | 33.00 (19.96, 49.68) | 3127.56 (1949.37, 4731.87) | 38.45 (23.55, 59.05) | 0.51 (0.48, 0.53) |
| Greenland | 9.23 (5.62, 14.23) | 27.50 (17.15, 41.67) | 18.39 (11.74, 26.86) | 37.56 (23.32, 56.24) | 1.21 (1.09, 1.32) |
| Grenada | 5.54 (3.29, 8.71) | 13.84 (8.26, 21.66) | 13.93 (8.24, 21.69) | 18.60 (10.85, 29.13) | 0.91 (0.85, 0.97) |
| Guam | 8.49 (4.49, 13.95) | 10.46 (5.57, 17.11) | 15.64 (8.46, 25.19) | 14.11 (7.58, 22.89) | 1.01 (0.92, 1.10) |
| Guatemala | 980.96 (702.78, 1376.20) | 30.16 (21.89, 41.73) | 3386.38 (2271.78, 4902.65) | 40.60 (27.70, 58.04) | 0.94 (0.84, 1.03) |
| Guinea | 206.90 (109.48, 349.08) | 8.00 (4.26, 13.43) | 521.40 (274.22, 872.36) | 9.35 (4.95, 15.56) | 0.54 (0.43, 0.65) |
| Guinea-Bissau | 31.20 (16.26, 51.56) | 7.99 (4.22, 13.16) | 83.51 (44.66, 140.06) | 9.50 (5.12, 15.80) | 0.58 (0.46, 0.70) |
| Guyana | 40.74 (21.19, 68.15) | 10.84 (5.74, 17.89) | 82.80 (50.11, 128.08) | 17.02 (10.30, 26.32) | 1.23 (0.98, 1.49) |
| Haiti | 708.69 (416.26, 1180.52) | 24.97 (14.82, 41.41) | 1734.26 (1012.21, 2776.40) | 25.20 (14.90, 40.28) | 0.10 (0.04, 0.16) |
| Honduras | 1036.75 (698.64, 1465.36) | 59.54 (40.79, 83.19) | 4111.87 (2739.43, 5860.28) | 80.80 (54.58, 114.00) | 0.93 (0.84, 1.02) |
| Hungary | 3237.40 (2314.52, 4489.86) | 40.11 (28.24, 56.28) | 3211.55 (2095.55, 4758.60) | 39.87 (25.41, 60.15) | -0.08 (-0.17, 0.00) |
| Iceland | 63.85 (39.52, 96.65) | 40.30 (25.01, 60.85) | 114.76 (69.12, 174.69) | 43.56 (25.78, 67.22) | 0.36 (0.32, 0.40) |
| India | 101988.25 (68794.63, 145965.24) | 24.79 (16.99, 34.92) | 267578.31 (178280.97, 387381.83) | 30.31 (20.34, 43.62) | 0.72 (0.60, 0.84) |
| Indonesia | 6913.07 (4382.35, 10436.24) | 7.39 (4.75, 11.03) | 17699.20 (11338.04, 26493.20) | 8.94 (5.73, 13.38) | 0.62 (0.60, 0.64) |
| Iran (Islamic Republic of) | 3100.37 (1964.78, 4639.53) | 12.33 (7.91, 18.28) | 10383.93 (6671.40, 15370.80) | 16.67 (10.74, 24.63) | 1.03 (1.01, 1.05) |
| Iraq | 1146.71 (640.13, 1864.07) | 14.60 (8.40, 23.29) | 4542.29 (2542.17, 7198.21) | 18.92 (10.74, 29.75) | 0.98 (0.80, 1.15) |
| Ireland | 1570.51 (1082.35, 2216.77) | 72.67 (50.09, 102.59) | 2849.39 (1827.17, 4138.60) | 72.69 (45.94, 106.89) | 0.24 (0.11, 0.37) |
| Israel | 788.36 (463.89, 1236.53) | 28.22 (16.71, 44.02) | 2040.16 (1211.64, 3177.89) | 34.08 (20.13, 53.20) | 0.66 (0.62, 0.69) |
| Italy | 17176.98 (11588.71, 24550.23) | 38.73 (25.80, 55.92) | 18702.71 (12526.15, 26953.46) | 36.08 (23.70, 52.83) | -0.23 (-0.25, -0.21) |
| Jamaica | 194.48 (113.68, 304.23) | 17.42 (10.43, 26.84) | 484.48 (303.12, 732.44) | 24.89 (15.57, 37.63) | 1.01 (0.86, 1.16) |
| Japan | 53862.38 (37982.27, 75017.00) | 51.67 (36.14, 72.60) | 43228.08 (28506.51, 62485.96) | 42.84 (27.82, 62.85) | -0.55 (-0.60, -0.49) |
| Jordan | 219.01 (119.15, 356.97) | 13.72 (7.66, 22.17) | 1551.19 (882.51, 2426.92) | 20.26 (11.63, 31.49) | 1.54 (1.43, 1.65) |
| Kazakhstan | 1319.70 (704.22, 2192.74) | 13.51 (7.21, 22.44) | 2794.64 (1714.12, 4272.28) | 21.68 (13.24, 33.23) | 1.58 (1.14, 2.02) |
| Kenya | 963.70 (578.10, 1524.17) | 12.21 (7.54, 18.89) | 3163.53 (1922.75, 4916.60) | 13.75 (8.54, 21.03) | 0.36 (0.26, 0.46) |
| Kiribati | 2.89 (1.58, 4.72) | 7.76 (4.28, 12.56) | 6.46 (3.60, 10.47) | 9.20 (5.16, 14.84) | 0.47 (0.42, 0.52) |
| Kuwait | 288.79 (166.96, 455.59) | 27.00 (15.97, 41.91) | 1835.24 (1128.64, 2790.71) | 46.49 (28.50, 70.60) | 2.08 (1.91, 2.25) |
| Kyrgyzstan | 895.34 (562.46, 1342.53) | 40.61 (25.84, 60.32) | 2346.53 (1524.98, 3466.55) | 57.76 (37.73, 84.94) | 1.44 (1.22, 1.66) |
| Lao People's Democratic Republic | 221.94 (137.97, 337.22) | 12.41 (7.82, 18.64) | 591.53 (358.47, 920.04) | 14.00 (8.57, 21.61) | 0.35 (0.31, 0.40) |
| Latvia | 1103.18 (796.73, 1482.85) | 53.42 (38.15, 72.77) | 959.71 (685.87, 1307.21) | 60.00 (41.81, 83.47) | 0.14 (-0.08, 0.35) |
| Lebanon | 390.95 (230.21, 603.06) | 23.24 (13.77, 35.74) | 1143.84 (674.20, 1781.93) | 30.19 (17.81, 46.96) | 1.06 (0.92, 1.20) |
| Lesotho | 221.96 (140.31, 325.45) | 34.12 (21.83, 49.54) | 377.23 (238.27, 554.84) | 40.62 (25.83, 59.16) | 0.62 (0.49, 0.75) |
| Liberia | 87.47 (46.97, 145.49) | 8.67 (4.71, 14.35) | 280.24 (146.80, 468.56) | 10.80 (5.77, 17.89) | 0.96 (0.77, 1.15) |
| Libya | 249.26 (137.51, 402.72) | 13.18 (7.43, 21.05) | 1037.16 (631.45, 1608.76) | 20.51 (12.53, 31.69) | 1.68 (1.60, 1.76) |
| Lithuania | 1756.58 (1272.30, 2400.07) | 65.70 (47.20, 90.43) | 1786.77 (1313.51, 2413.42) | 75.00 (53.88, 104.07) | 0.18 (0.04, 0.32) |
| Luxembourg | 102.48 (65.02, 153.52) | 34.85 (21.87, 52.61) | 198.54 (120.45, 303.49) | 37.21 (22.19, 57.46) | 0.35 (0.30, 0.41) |
| Madagascar | 475.94 (254.88, 816.27) | 10.37 (5.67, 17.56) | 1457.67 (776.93, 2378.61) | 11.72 (6.36, 18.86) | 0.40 (0.35, 0.45) |
| Malawi | 441.16 (237.94, 749.43) | 12.19 (6.68, 20.49) | 1101.78 (603.24, 1834.47) | 14.58 (8.18, 23.83) | 0.61 (0.55, 0.67) |
| Malaysia | 622.48 (356.34, 985.63) | 7.20 (4.21, 11.26) | 1902.24 (1052.82, 3080.67) | 8.98 (4.98, 14.48) | 0.73 (0.69, 0.78) |
| Maldives | 16.65 (10.12, 25.51) | 18.85 (11.58, 28.60) | 65.08 (37.99, 102.45) | 18.02 (10.74, 27.83) | 0.05 (-0.23, 0.33) |
| Mali | 267.71 (137.62, 444.70) | 7.42 (3.85, 12.22) | 833.22 (431.10, 1387.73) | 9.01 (4.70, 14.88) | 0.72 (0.63, 0.81) |
| Malta | 86.10 (53.13, 129.05) | 33.46 (20.62, 50.25) | 127.58 (78.49, 195.95) | 37.11 (22.33, 57.95) | 0.36 (0.32, 0.40) |
| Marshall Islands | 1.59 (0.84, 2.68) | 9.04 (4.85, 15.09) | 4.09 (2.23, 6.75) | 11.90 (6.53, 19.57) | 0.84 (0.78, 0.90) |
| Mauritania | 78.75 (40.65, 131.50) | 9.27 (4.80, 15.31) | 251.09 (131.12, 419.48) | 12.85 (6.80, 21.28) | 1.10 (0.99, 1.20) |
| Mauritius | 61.26 (32.36, 102.26) | 9.97 (5.31, 16.55) | 197.67 (127.79, 293.56) | 18.53 (11.75, 27.96) | 1.50 (1.07, 1.93) |
| Mexico | 30851.38 (22865.63, 41698.30) | 85.17 (63.93, 113.63) | 81784.12 (59921.24, 110266.32) | 93.09 (68.22, 125.52) | 0.25 (0.16, 0.35) |
| Micronesia (Federated States of) | 4.54 (2.45, 7.51) | 10.24 (5.55, 16.85) | 9.17 (4.99, 15.07) | 14.29 (7.78, 23.46) | 1.02 (0.97, 1.08) |
| Monaco | 6.11 (3.48, 9.53) | 26.19 (14.73, 41.20) | 8.40 (4.97, 13.25) | 28.85 (16.69, 46.51) | 0.42 (0.31, 0.53) |
| Mongolia | 184.66 (109.37, 299.24) | 20.10 (12.14, 32.43) | 643.29 (400.38, 961.54) | 29.91 (18.69, 44.54) | 1.67 (1.49, 1.85) |
| Montenegro | 104.71 (63.24, 159.77) | 24.69 (14.81, 37.82) | 141.03 (86.61, 214.71) | 28.68 (17.26, 44.35) | 0.69 (0.58, 0.81) |
| Morocco | 1622.78 (903.09, 2645.14) | 12.97 (7.29, 21.03) | 4899.38 (2795.71, 7714.22) | 19.62 (11.18, 30.91) | 1.48 (1.42, 1.54) |
| Mozambique | 620.85 (335.26, 1063.64) | 11.41 (6.26, 19.34) | 1555.88 (859.47, 2561.11) | 13.43 (7.58, 21.80) | 0.47 (0.43, 0.51) |
| Myanmar | 2406.92 (1492.43, 3651.88) | 11.98 (7.49, 18.00) | 5212.34 (3123.36, 8035.12) | 14.24 (8.54, 21.95) | 0.52 (0.47, 0.56) |
| Namibia | 186.69 (117.65, 275.39) | 33.09 (21.23, 48.04) | 435.96 (267.94, 656.95) | 34.58 (21.61, 51.56) | 0.01 (-0.09, 0.11) |
| Nauru | 0.56 (0.30, 0.93) | 11.16 (6.03, 18.56) | 0.85 (0.45, 1.40) | 14.51 (7.80, 23.83) | 0.73 (0.54, 0.92) |
| Nepal | 1899.16 (1125.59, 2913.99) | 22.41 (13.58, 33.88) | 5668.32 (3325.52, 8858.19) | 31.88 (18.98, 49.27) | 1.27 (1.11, 1.44) |
| Netherlands | 5890.08 (3887.55, 8496.03) | 55.22 (36.36, 79.75) | 7459.87 (4759.24, 10976.11) | 52.01 (32.41, 78.16) | 0.03 (-0.07, 0.14) |
| New Zealand | 1333.15 (901.19, 1910.19) | 60.59 (41.01, 86.77) | 2242.49 (1448.78, 3286.69) | 55.44 (35.21, 82.39) | -0.17 (-0.28, -0.06) |
| Nicaragua | 494.04 (325.91, 720.74) | 35.03 (23.65, 50.18) | 1646.73 (1069.52, 2434.16) | 42.79 (28.19, 62.61) | 0.77 (0.70, 0.84) |
| Niger | 215.67 (113.90, 358.67) | 7.16 (3.86, 11.79) | 741.96 (387.36, 1235.39) | 8.44 (4.46, 13.98) | 0.64 (0.54, 0.73) |
| Nigeria | 3158.53 (1896.45, 4961.04) | 7.98 (4.87, 12.40) | 10372.79 (6283.63, 15967.39) | 10.38 (6.38, 15.80) | 0.95 (0.81, 1.09) |
| Niue | 0.13 (0.07, 0.22) | 10.35 (5.58, 17.18) | 0.17 (0.09, 0.27) | 14.12 (7.70, 23.38) | 1.00 (0.99, 1.02) |
| North Macedonia | 221.46 (127.70, 355.18) | 16.62 (9.56, 26.67) | 387.55 (219.85, 616.91) | 21.35 (11.94, 34.28) | 0.99 (0.89, 1.09) |
| Northern Mariana Islands | 3.81 (2.03, 6.37) | 13.39 (7.33, 21.96) | 5.85 (3.30, 9.34) | 15.52 (8.63, 24.88) | 0.33 (0.20, 0.47) |
| Norway | 1872.69 (1319.56, 2624.55) | 64.60 (45.38, 90.84) | 2211.97 (1443.48, 3252.51) | 51.08 (32.93, 75.97) | -0.79 (-0.88, -0.71) |
| Oman | 84.93 (46.78, 137.12) | 9.29 (5.25, 14.83) | 505.14 (281.57, 817.95) | 15.74 (9.00, 24.96) | 1.91 (1.85, 1.98) |
| Pakistan | 17600.42 (11217.49, 26027.40) | 37.11 (24.08, 54.05) | 43489.17 (27623.61, 64289.63) | 35.54 (22.86, 51.83) | 0.09 (0.02, 0.16) |
| Palau | 1.03 (0.55, 1.70) | 11.87 (6.40, 19.46) | 2.36 (1.26, 3.75) | 15.26 (8.11, 24.47) | 0.74 (0.70, 0.78) |
| Palestine | 123.32 (69.62, 199.05) | 15.58 (8.98, 24.83) | 522.74 (288.01, 839.68) | 19.42 (10.94, 30.88) | 0.71 (0.58, 0.83) |
| Panama | 323.24 (204.96, 484.41) | 27.95 (18.15, 41.16) | 983.34 (635.12, 1449.61) | 35.57 (22.95, 52.50) | 0.75 (0.71, 0.80) |
| Papua New Guinea | 140.03 (75.43, 229.87) | 7.17 (3.91, 11.68) | 479.28 (260.48, 786.71) | 8.48 (4.63, 13.83) | 0.47 (0.42, 0.53) |
| Paraguay | 729.17 (454.14, 1094.58) | 39.54 (25.07, 58.56) | 2907.11 (1905.77, 4243.43) | 67.00 (44.35, 97.04) | 1.67 (1.53, 1.81) |
| Peru | 6621.67 (4213.84, 9693.31) | 61.97 (40.10, 89.69) | 22784.85 (14440.67, 33873.51) | 96.74 (61.52, 143.40) | 1.61 (1.51, 1.72) |
| Philippines | 5640.57 (3812.65, 8146.61) | 19.25 (13.16, 27.51) | 11310.91 (7646.54, 16384.30) | 16.54 (11.25, 23.83) | -0.30 (-0.38, -0.23) |
| Poland | 18157.21 (13924.53, 23748.65) | 67.99 (51.84, 89.35) | 14913.31 (10200.99, 21151.91) | 47.16 (31.77, 67.76) | -1.09 (-1.23, -0.95) |
| Portugal | 2611.12 (1712.92, 3735.21) | 35.75 (23.12, 51.74) | 3942.60 (2500.36, 5801.42) | 44.87 (27.54, 67.52) | 0.83 (0.75, 0.92) |
| Puerto Rico | 576.29 (354.74, 880.97) | 26.11 (16.12, 39.82) | 974.85 (611.78, 1462.47) | 38.44 (23.52, 58.74) | 1.18 (1.05, 1.31) |
| Qatar | 34.34 (18.84, 55.58) | 12.31 (7.04, 19.40) | 488.08 (279.31, 780.50) | 20.26 (11.77, 32.17) | 1.32 (1.11, 1.52) |
| Republic of Korea | 9042.93 (5986.81, 13178.83) | 34.64 (23.39, 49.76) | 18513.74 (11699.53, 27637.74) | 37.25 (23.08, 56.57) | 0.36 (0.30, 0.43) |
| Republic of Moldova | 563.75 (360.83, 844.07) | 19.20 (12.25, 28.85) | 836.03 (546.20, 1220.82) | 26.93 (17.24, 39.91) | 0.85 (0.64, 1.06) |
| Romania | 2473.94 (1370.40, 4084.78) | 14.97 (8.22, 24.91) | 3266.45 (1885.01, 5188.82) | 21.88 (12.45, 35.12) | 1.36 (1.28, 1.45) |
| Russian Federation | 47932.27 (36399.26, 62947.54) | 42.68 (32.06, 56.55) | 53660.79 (39526.06, 72119.91) | 45.10 (32.63, 61.44) | 0.05 (-0.05, 0.14) |
| Rwanda | 343.75 (184.38, 608.36) | 13.12 (7.17, 22.91) | 959.65 (528.64, 1563.03) | 15.81 (8.86, 25.38) | 0.77 (0.64, 0.89) |
| Saint Kitts and Nevis | 5.35 (3.59, 7.63) | 28.14 (19.10, 39.64) | 16.10 (10.36, 23.37) | 32.21 (20.36, 47.40) | 0.36 (0.27, 0.45) |
| Saint Lucia | 14.28 (9.31, 20.81) | 23.56 (15.72, 33.76) | 41.49 (27.21, 60.75) | 28.81 (18.61, 42.67) | 0.54 (0.42, 0.65) |
| Saint Vincent and the Grenadines | 7.41 (4.41, 11.36) | 15.08 (9.14, 22.80) | 14.84 (8.97, 23.14) | 17.90 (10.68, 28.19) | 0.66 (0.53, 0.79) |
| Samoa | 8.35 (4.41, 13.78) | 11.08 (5.89, 18.15) | 15.52 (8.32, 25.40) | 13.61 (7.32, 22.21) | 0.58 (0.53, 0.63) |
| San Marino | 4.41 (2.59, 6.91) | 26.00 (15.14, 40.91) | 7.29 (4.31, 11.33) | 27.99 (16.14, 44.10) | 0.38 (0.30, 0.46) |
| Sao Tome and Principe | 4.46 (2.33, 7.52) | 9.24 (4.85, 15.52) | 13.42 (6.75, 22.49) | 11.93 (6.08, 19.90) | 0.89 (0.76, 1.01) |
| Saudi Arabia | 820.00 (459.45, 1325.22) | 10.97 (6.29, 17.49) | 5430.71 (3167.26, 8578.75) | 18.75 (11.09, 29.30) | 1.86 (1.76, 1.95) |
| Senegal | 252.35 (131.74, 421.25) | 8.52 (4.47, 14.08) | 761.76 (399.88, 1273.18) | 10.45 (5.57, 17.35) | 0.64 (0.55, 0.73) |
| Serbia | 1636.11 (1081.37, 2345.02) | 21.62 (14.06, 31.34) | 2073.08 (1292.61, 3115.36) | 28.49 (17.35, 43.52) | 0.84 (0.65, 1.03) |
| Seychelles | 3.38 (1.89, 5.52) | 8.84 (4.97, 14.32) | 9.15 (5.05, 14.87) | 11.36 (6.25, 18.49) | 0.80 (0.75, 0.85) |
| Sierra Leone | 141.38 (74.16, 237.40) | 7.96 (4.22, 13.23) | 370.69 (192.01, 621.07) | 9.28 (4.88, 15.40) | 0.50 (0.37, 0.63) |
| Singapore | 392.31 (225.64, 624.34) | 19.77 (11.68, 31.03) | 1239.86 (708.01, 1961.96) | 24.55 (13.76, 39.33) | 0.74 (0.71, 0.78) |
| Slovakia | 753.22 (473.85, 1133.12) | 21.41 (13.42, 32.23) | 1114.60 (686.86, 1710.74) | 24.97 (15.04, 38.87) | 0.54 (0.44, 0.64) |
| Slovenia | 680.85 (477.31, 958.48) | 45.20 (31.34, 64.20) | 891.70 (569.69, 1327.78) | 48.13 (29.91, 73.20) | 0.21 (0.06, 0.37) |
| Solomon Islands | 13.08 (7.02, 21.49) | 9.19 (4.98, 15.05) | 43.63 (23.84, 70.78) | 12.20 (6.74, 19.62) | 0.83 (0.78, 0.89) |
| Somalia | 373.74 (203.97, 648.16) | 13.22 (7.44, 22.73) | 947.74 (507.85, 1584.96) | 13.01 (7.22, 21.53) | -0.04 (-0.06, -0.02) |
| South Africa | 16099.11 (11836.55, 21368.95) | 88.64 (65.63, 116.91) | 24325.07 (17342.66, 33225.55) | 65.62 (47.06, 89.21) | -1.04 (-1.14, -0.93) |
| South Sudan | 243.37 (130.01, 411.53) | 11.09 (6.04, 18.49) | 522.62 (282.25, 852.11) | 12.76 (6.99, 20.57) | 0.53 (0.47, 0.59) |
| Spain | 9440.40 (6404.84, 13229.65) | 33.77 (22.61, 47.86) | 14194.98 (9268.51, 20570.16) | 35.47 (22.73, 52.04) | 0.13 (0.10, 0.16) |
| Sri Lanka | 724.00 (410.70, 1169.00) | 7.55 (4.34, 12.10) | 1538.81 (831.52, 2507.89) | 9.72 (5.24, 15.91) | 0.91 (0.82, 1.00) |
| Sudan | 990.47 (535.36, 1594.12) | 11.39 (6.27, 18.17) | 3648.80 (2123.06, 5746.16) | 16.76 (9.99, 26.07) | 1.43 (1.32, 1.54) |
| Suriname | 26.71 (15.45, 42.67) | 12.89 (7.52, 20.46) | 62.82 (36.47, 98.69) | 15.46 (8.91, 24.45) | 0.66 (0.56, 0.75) |
| Sweden | 3396.17 (2301.11, 4832.27) | 54.84 (36.86, 78.60) | 3522.11 (2278.41, 5160.69) | 45.01 (28.68, 66.69) | -0.66 (-0.71, -0.62) |
| Switzerland | 2049.70 (1286.41, 3102.61) | 39.97 (24.95, 60.79) | 3067.93 (1887.15, 4589.25) | 41.14 (24.78, 62.50) | 0.18 (0.14, 0.22) |
| Syrian Arab Republic | 735.91 (424.05, 1152.26) | 13.84 (8.07, 21.47) | 1945.77 (1156.11, 3032.13) | 20.92 (12.19, 33.02) | 1.44 (1.31, 1.57) |
| Taiwan (Province of China) | 3914.41 (2615.58, 5628.49) | 31.93 (21.48, 45.70) | 6007.95 (4133.87, 8349.69) | 26.98 (18.33, 37.83) | 0.13 (-0.13, 0.40) |
| Tajikistan | 470.56 (257.57, 757.89) | 19.93 (11.16, 31.80) | 1526.90 (898.16, 2402.85) | 26.44 (15.71, 41.37) | 0.83 (0.79, 0.87) |
| Thailand | 4945.44 (3049.43, 7629.16) | 15.72 (9.80, 24.10) | 11459.13 (7130.26, 17388.22) | 19.05 (11.60, 29.41) | 0.62 (0.57, 0.68) |
| Timor-Leste | 33.33 (19.86, 51.83) | 9.90 (6.01, 15.17) | 79.63 (46.87, 124.33) | 12.24 (7.30, 18.97) | 0.76 (0.69, 0.83) |
| Togo | 112.01 (57.46, 189.80) | 8.21 (4.30, 13.82) | 412.13 (215.95, 676.82) | 10.09 (5.32, 16.48) | 0.69 (0.56, 0.82) |
| Tokelau | 0.08 (0.04, 0.12) | 9.21 (4.83, 15.23) | 0.11 (0.06, 0.18) | 12.70 (6.72, 20.73) | 1.06 (1.02, 1.11) |
| Tonga | 4.71 (2.48, 7.95) | 10.40 (5.51, 17.41) | 7.60 (4.02, 12.70) | 13.36 (7.10, 22.29) | 0.68 (0.63, 0.73) |
| Trinidad and Tobago | 227.18 (159.25, 320.24) | 37.41 (26.82, 51.86) | 470.49 (325.20, 665.26) | 43.08 (29.42, 61.60) | 0.38 (0.29, 0.46) |
| Tunisia | 548.85 (303.53, 890.65) | 12.85 (7.16, 20.75) | 1619.06 (924.09, 2528.80) | 18.99 (10.76, 29.83) | 1.42 (1.33, 1.51) |
| Turkiye | 10598.44 (6996.90, 15056.93) | 35.31 (23.55, 49.77) | 25535.36 (16143.35, 37499.73) | 42.11 (26.51, 62.02) | 0.68 (0.56, 0.80) |
| Turkmenistan | 202.96 (107.56, 333.74) | 11.68 (6.23, 19.12) | 563.86 (328.70, 890.19) | 17.04 (9.95, 26.87) | 1.41 (1.22, 1.60) |
| Tuvalu | 0.47 (0.25, 0.77) | 8.96 (4.80, 14.81) | 0.88 (0.47, 1.43) | 11.88 (6.37, 19.28) | 0.86 (0.83, 0.89) |
| Uganda | 672.29 (356.10, 1124.77) | 11.40 (6.18, 18.77) | 2178.58 (1180.52, 3575.71) | 13.98 (7.70, 22.55) | 0.72 (0.62, 0.81) |
| Ukraine | 7820.43 (5367.94, 11036.00) | 19.57 (13.25, 27.96) | 8607.72 (5839.27, 12329.73) | 23.52 (15.71, 34.10) | 0.36 (0.22, 0.50) |
| United Arab Emirates | 157.17 (92.51, 242.47) | 15.40 (9.56, 22.96) | 1704.14 (1006.89, 2595.83) | 19.50 (11.42, 30.11) | 0.80 (0.66, 0.95) |
| United Kingdom | 30334.51 (22239.01, 40931.79) | 73.41 (53.34, 99.82) | 36256.45 (25126.84, 50733.52) | 68.02 (46.50, 96.29) | -0.06 (-0.17, 0.05) |
| United Republic of Tanzania | 1161.47 (627.73, 1942.72) | 12.12 (6.66, 20.08) | 3569.56 (1944.03, 5804.24) | 14.46 (8.02, 23.21) | 0.53 (0.47, 0.60) |
| United States of America | 68533.34 (48935.08, 93750.13) | 41.21 (29.44, 56.35) | 116941.67 (82374.38, 160696.40) | 44.61 (31.13, 61.77) | 0.53 (0.41, 0.65) |
| United States Virgin Islands | 12.29 (7.11, 19.31) | 18.17 (10.55, 28.49) | 14.65 (8.57, 23.08) | 22.17 (12.59, 35.43) | 0.69 (0.66, 0.72) |
| Uruguay | 710.85 (495.15, 987.13) | 34.39 (23.70, 48.17) | 1128.68 (793.21, 1578.15) | 45.33 (31.39, 64.08) | 0.95 (0.86, 1.04) |
| Uzbekistan | 3946.74 (2328.40, 6108.37) | 42.44 (25.49, 64.65) | 12820.16 (8193.09, 19063.80) | 56.98 (36.54, 84.59) | 1.23 (1.10, 1.36) |
| Vanuatu | 5.83 (3.11, 9.62) | 8.64 (4.64, 14.15) | 18.05 (9.59, 29.59) | 10.82 (5.80, 17.64) | 0.72 (0.69, 0.75) |
| Venezuela (Bolivarian Republic of) | 4131.74 (2837.92, 5857.35) | 49.82 (35.25, 68.97) | 12602.73 (8669.49, 17757.53) | 64.63 (44.18, 91.68) | 0.71 (0.62, 0.81) |
| Viet Nam | 4451.92 (2606.67, 7044.05) | 14.28 (8.41, 22.51) | 14748.17 (8661.66, 23027.54) | 20.13 (11.78, 31.53) | 1.12 (1.07, 1.17) |
| Yemen | 507.37 (289.42, 804.78) | 9.88 (5.72, 15.54) | 2080.89 (1199.57, 3267.71) | 13.28 (7.86, 20.60) | 1.15 (1.07, 1.23) |
| Zambia | 357.38 (191.29, 607.93) | 12.69 (6.94, 21.21) | 1198.12 (644.95, 1941.09) | 15.45 (8.55, 24.60) | 0.65 (0.51, 0.78) |
| Zimbabwe | 986.05 (624.42, 1454.42) | 25.73 (16.73, 37.27) | 1998.13 (1253.29, 2920.28) | 28.45 (18.02, 40.93) | 0.34 (0.13, 0.54) |
