# Supplementary material for: Global burden and regional disparities of rheumatoid arthritis among the working-age population: A comprehensive analysis from 1990 to 2021 with projections to 2040
Source: PLoS One. 2025 Jun 4;20(6):e0325127. doi: 10.1371/journal.pone.0325127 (PMC12136291; doi:10.1371/journal.pone.0325127)
Supplement: S5 Table — (DOCX) [file pone.0325127.s020.docx]

**S5 Table.** Relative risks of rheumatoid arthritis incidence, prevalence mortality and DALYs for both sexes due to age, period, and birth cohort effects

| **Factor** | **Incidence** | **Prevalence** | **Mortality** | **DALYs** |
| --- | --- | --- | --- | --- |
|  | **Relative risk (95% CI)** | **Relative risk (95% CI)** | **Relative risk (95% CI)** | **Relative risk (95% CI)** |
| **Age (years)** | | | | |
| 15−19 | 0.44 (0.44−0.44) | 0.21 (0.21−0.21) | 0.22 (0.20−0.24) | 0.22 (0.22−0.22） |
| 20−24 | 0.61 (0.61−0.62) | 0.40 (0.40−0.40) | 0.24 (0.22−0.26) | 0.39 (0.39−0.39） |
| 25−29 | 0.75 (0.75−075) | 0.62 (0.62−0.62) | 0.30 (0.28−0.33) | 0.59 (0.58−0.59） |
| 30−34 | 0.88 (0.88−0.89) | 0.85 (0.85−0.85) | 0.43 (0.40−0.46) | 0.80 (0.80−0.80） |
| 35−39 | 1.02 (1.02−1.03) | 1.10 (1.10−1.10) | 0.65 (0.62−0.69) | 1.04 (1.04−1.04） |
| 40−44 | 1.14 (1.13−1.14) | 1.36 (1.36−1.36) | 1.02 (0.98−1.07) | 1.30 (1.30−1.30） |
| 45−49 | 1.23 (1.22−1.23) | 1.61 (1.61−1.61) | 1.70 (1.64−1.77) | 1.58 (1.57−1.58） |
| 50−54 | 1.37 (1.37−1.38) | 1.86 (1.86−1.86) | 2.93 (2.84−3.02) | 1.89 (1.89−1.89） |
| 55−59 | 1.57 (1.57−1.58) | 2.12 (2.12−2.12) | 4.99 (4.85−5.14) | 2.25 (2.24−2.25） |
| 60−64 | 1.82 (1.81−1.82) | 2.39 (2.39−2.39) | 9.09 (8.80−9.38) | 2.72 (2.71−2.72） |
| **Birth cohort** | | | | |
| 1932 to 1936 | 1.24 (1.23−1.25) | 1.61 (1.61−1.61) | 2.03 (1.92−2.15) | 1.69 (1.69−1.70） |
| 1937 to 1941 | 1.22 (1.22−1.23) | 1.49 (1.49−1.49) | 1.87 (1.79−1.95) | 1.54 (1.53−1.54） |
| 1942 to 1946 | 1.22 (1.21−1.22) | 1.40 (1.40−1.40) | 1.69 (1.63−1.76) | 1.42 (1.41−1.42） |
| 1947 to 1951 | 1.20 (1.20−1.21) | 1.329 (1.33−1.33) | 1.55 (1.50−1.61) | 1.32 (1.32−1.32） |
| 1952 to 1956 | 1.14 (1.13−1.14) | 1.23 (1.23−1.23) | 1.44 (1.39−1.49) | 1.22 (1.21−1.22） |
| 1957 to 1961 | 1.08 (1.08−1.08) | 1.13 (1.13−1.14) | 1.31 (1.26−1.36) | 1.12 (1.11−1.12） |
| 1962 to 1966 | 1.04 (1.03−1.04) | 1.06 (1.06−1.06) | 1.19 (1.14−1.24) | 1.05 (1.04−1.05） |
| 1967 to 1971 | 1.00 (1.00−1.00) | 1.00 (1.00−1.00) | 1.09 (1.03−1.15) | 0.99 (0.99−0.99） |
| 1972 to 1976 | 0.95 (0.95−0.96) | 0.93 (0.93−0.93) | 0.97 (0.90−1.03) | 0.92 (0.92−0.92） |
| 1977 to 1981 | 0.91 (0.90−0.91) | 0.86 (0.86−0.86) | 0.85 (0.79−0.92) | 0.86 (0.86−0.86） |
| 1982 to 1986 | 0.88 (0.87−0.88) | 0.81 (0.81−0.81) | 0.74 (0.67−0.80) | 0.81 (0.81−0.81） |
| 1987 to 1991 | 0.87 (0.86−0.87) | 0.77 (0.77−0.77) | 0.66 (0.59−0.73) | 0.78 (0.77−0.78） |
| 1992 to 1996 | 0.84 (0.84−0.84) | 0.72 (0.72−0.72) | 0.56 (0.49−0.64) | 0.72 (0.72−0.73） |
| 1997 to 2001 | 0.81 (0.81−0.82) | 0.67 (0.67−0.67) | 0.47 (0.39−0.55) | 0.67 (0.66−0.67） |
| 2002 to 2006 | 0.79 (0.78−0.80) | 0.62 (0.62−0.63) | 0.40 (0.30−0.54) | 0.61 (0.60−0.62） |
| **Period** | | | | |
| 1992 to 1996 | 0.87 (0.86−0.87) | 0.79 (0.79−0.79) | 0.95 (0.92−0.99) | 0.81 (0.81−0.81） |
| 1997 to 2001 | 0.91 (0.91−0.91) | 0.86 (0.86−0.86) | 0.95 (0.93−0.98) | 0.88 (0.87−0.88） |
| 2002 to 2006 | 0.97 (0.97−0.97) | 0.96 (0.96−0.96) | 1.06 (1.03−1.08) | 0.98 (0.97−0.98） |
| 2007 to 2011 | 1.04 (1.04−1.04) | 1.06 (1.06−1.06) | 1.03 (1.01−1.06) | 1.06 (1.06−1.06） |
| 2012 to 2016 | 1.10 (1.09−1.10) | 1.16 (1.16−1.16) | 1.00 (0.97−1.03) | 1.13 (1.18−1.13） |
| 2017 to 2021 | 1.15 (1.15−1.15) | 1.26 (1.26−1.26) | 1.01 (0.98−1.05) | 1.21 (1.20−1.21） |
