# Supplementary material for: Global burden and regional disparities of rheumatoid arthritis among the working-age population: A comprehensive analysis from 1990 to 2021 with projections to 2040
Source: PLoS One. 2025 Jun 4;20(6):e0325127. doi: 10.1371/journal.pone.0325127 (PMC12136291; doi:10.1371/journal.pone.0325127)
Supplement: S6 Table — (DOCX) [file pone.0325127.s021.docx]

**S6 Table.** Changes in incidence, prevalence, deaths and DALYs number of rheumatoid arthritis according to population-level determinants from 1990 to 2021

| **Location** | **Sex** | **Measure** | **Overall difference** | **Aging** | **Population** | **Epidemiological change** |
| --- | --- | --- | --- | --- | --- | --- |
| Global | Both | Incidence | 351577.84 (90.52%) | 54019.38 (13.91%) | 242276.42 (62.38%) | 55282.03 (14.23%) |
| Global | Male | Incidence | 103099.88 (93.61%) | 17520.78 (15.91%) | 68674.62 (62.36%) | 16904.47 (15.35%) |
| Global | Female | Incidence | 248477.96 (89.3%) | 36966.55 (13.29%) | 174609.73 (62.75%) | 36901.68 (13.26%) |
| Global | Both | Prevalence | 6178510.92 (108.39%) | 1316162.48 (23.09%) | 3732059.16 (65.47%) | 1130289.28 (19.83%) |
| Global | Male | Prevalence | 1693353.99 (109.67%) | 348034.65 (22.54%) | 1004824.61 (65.08%) | 340494.72 (22.05%) |
| Global | Female | Prevalence | 4485156.93 (107.91%) | 988723.03 (23.79%) | 2743573.85 (66.01%) | 752860.05 (18.11%) |
| Global | Both | Deaths | 1003.23 (17.09%) | 1530.96 (26.08%) | 2944.42 (50.16%) | -3472.14 (-59.15%) |
| Global | Male | Deaths | 541.05 (31.58%) | 453.72 (26.48%) | 892.18 (52.07%) | -804.85 (-46.97%) |
| Global | Female | Deaths | 462.18 (11.12%) | 1096.23 (26.37%) | 2065.21 (49.68%) | -2699.26 (-64.93%) |
| Global | Both | DALYs | 854035.09 (86.16%) | 223165.24 (22.51%) | 611547.68 (61.69%) | 19322.17 (1.95%) |
| Global | Male | DALYs | 248022.84 (89.66%) | 61116.48 (22.09%) | 170682.43 (61.7%) | 16223.93 (5.87%) |
| Global | Female | DALYs | 606012.25 (84.8%) | 165372.38 (23.14%) | 443501.64 (62.06%) | -2861.77 (-0.4%) |
| High SDI | Both | Incidence | 52739.95 (45.35%) | 19478.63 (16.75%) | 27659.8 (23.79%) | 5601.52 (4.82%) |
| High SDI | Male | Incidence | 16787.55 (55.55%) | 6324.84 (20.93%) | 7958.13 (26.33%) | 2504.58 (8.29%) |
| High SDI | Female | Incidence | 35952.4 (41.77%) | 12796.75 (14.87%) | 18778.97 (21.82%) | 4376.68 (5.09%) |
| High SDI | Both | Prevalence | 941100.11 (57.06%) | 358365.67 (21.73%) | 408758.01 (24.78%) | 173976.43 (10.55%) |
| High SDI | Male | Prevalence | 256686.92 (63.94%) | 95163.55 (23.7%) | 108725.29 (27.08%) | 52798.08 (13.15%) |
| High SDI | Female | Prevalence | 684413.19 (54.85%) | 256565.94 (20.56%) | 285277.17 (22.86%) | 142570.07 (11.43%) |
| High SDI | Both | Deaths | -530.99 (-34.13%) | 399.2 (25.66%) | 271 (17.42%) | -1201.18 (-77.2%) |
| High SDI | Male | Deaths | -86.55 (-19.43%) | 126.38 (28.38%) | 88.9 (19.96%) | -301.83 (-67.78%) |
| High SDI | Female | Deaths | -444.44 (-40.02%) | 267.47 (24.08%) | 173.93 (15.66%) | -885.85 (-79.77%) |
| High SDI | Both | DALYs | 105533.34 (38.27%) | 59355.86 (21.52%) | 64106.27 (23.25%) | -17928.79 (-6.5%) |
| High SDI | Male | DALYs | 30976.34 (44.02%) | 16440.68 (23.37%) | 17840.08 (25.35%) | -3304.42 (-4.7%) |
| High SDI | Female | DALYs | 74556.99 (36.3%) | 41878.5 (20.39%) | 44036.04 (21.44%) | -11357.54 (-5.53%) |
| High-middle SDI | Both | Incidence | 59983.57 (69.95%) | 12909.41 (15.06%) | 25916.99 (30.23%) | 21157.17 (24.67%) |
| High-middle SDI | Male | Incidence | 18081.9 (76.85%) | 4182.84 (17.78%) | 7601.31 (32.31%) | 6297.74 (26.77%) |
| High-middle SDI | Female | Incidence | 41901.67 (67.35%) | 8593.5 (13.81%) | 17802.38 (28.61%) | 15505.79 (24.92%) |
| High-middle SDI | Both | Prevalence | 1246146.14 (91.66%) | 416578.38 (30.64%) | 439730.25 (32.34%) | 389837.51 (28.67%) |
| High-middle SDI | Male | Prevalence | 354239.56 (99.01%) | 108268.3 (30.26%) | 123444.06 (34.5%) | 122527.2 (34.24%) |
| High-middle SDI | Female | Prevalence | 891906.58 (89.03%) | 307981.35 (30.74%) | 307066.7 (30.65%) | 276858.54 (27.64%) |
| High-middle SDI | Both | Deaths | 4.8 (0.33%) | 471.7 (31.96%) | 351.35 (23.81%) | -818.25 (-55.44%) |
| High-middle SDI | Male | Deaths | 120.77 (34.13%) | 127.54 (36.04%) | 99.81 (28.2%) | -106.58 (-30.12%) |
| High-middle SDI | Female | Deaths | -115.97 (-10.34%) | 341.64 (30.45%) | 243.8 (21.73%) | -701.4 (-62.52%) |
| High-middle SDI | Both | DALYs | 164607.08 (68.65%) | 70481.86 (29.4%) | 72317.26 (30.16%) | 21807.96 (9.1%) |
| High-middle SDI | Male | DALYs | 52047.03 (82.83%) | 18616.19 (29.63%) | 20676.05 (32.9%) | 12754.79 (20.3%) |
| High-middle SDI | Female | DALYs | 112560.05 (63.62%) | 51756.92 (29.25%) | 50139.68 (28.34%) | 10663.45 (6.03%) |
| Middle SDI | Both | Incidence | 128497.81 (106.74%) | 21160.05 (17.58%) | 76530.27 (63.57%) | 30807.49 (25.59%) |
| Middle SDI | Male | Incidence | 37803.98 (103.08%) | 7842.22 (21.38%) | 22504.41 (61.36%) | 7457.34 (20.33%) |
| Middle SDI | Female | Incidence | 90693.83 (108.35%) | 13459.17 (16.08%) | 54905.69 (65.59%) | 22328.98 (26.68%) |
| Middle SDI | Both | Prevalence | 2400826.71 (134.87%) | 687388.11 (38.62%) | 1216593.76 (68.34%) | 496844.84 (27.91%) |
| Middle SDI | Male | Prevalence | 667952.04 (127.32%) | 191388.16 (36.48%) | 342600.82 (65.3%) | 133963.06 (25.53%) |
| Middle SDI | Female | Prevalence | 1732874.67 (138.03%) | 512105.59 (40.79%) | 888904.96 (70.8%) | 331864.13 (26.43%) |
| Middle SDI | Both | Deaths | 874.83 (44.97%) | 893.18 (45.91%) | 1051.88 (54.07%) | -1070.23 (-55.01%) |
| Middle SDI | Male | Deaths | 342.46 (53.56%) | 289.2 (45.23%) | 344.75 (53.92%) | -291.49 (-45.59%) |
| Middle SDI | Female | Deaths | 532.38 (40.77%) | 622.03 (47.63%) | 718.83 (55.04%) | -808.48 (-61.91%) |
| Middle SDI | Both | DALYs | 347114.88 (108.21%) | 120089.57 (37.44%) | 205254.71 (63.99%) | 21770.61 (6.79%) |
| Middle SDI | Male | DALYs | 102097.07 (104.47%) | 35056.92 (35.87%) | 60268.23 (61.67%) | 6771.91 (6.93%) |
| Middle SDI | Female | DALYs | 245017.81 (109.85%) | 87721.42 (39.33%) | 147423.23 (66.09%) | 9873.16 (4.43%) |
| Low-middle SDI | Both | Incidence | 83449.81 (162.77%) | 4977.1 (9.71%) | 54921.73 (107.12%) | 23550.98 (45.94%) |
| Low-middle SDI | Male | Incidence | 21953.17 (149.19%) | 1408.67 (9.57%) | 15010.99 (102.01%) | 5533.51 (37.61%) |
| Low-middle SDI | Female | Incidence | 61496.64 (168.23%) | 3841.99 (10.51%) | 40377.28 (110.46%) | 17277.37 (47.26%) |
| Low-middle SDI | Both | Prevalence | 1229616.86 (173%) | 115714.57 (16.28%) | 777475.76 (109.38%) | 336426.54 (47.33%) |
| Low-middle SDI | Male | Prevalence | 305804.54 (156.83%) | 26677.99 (13.68%) | 202182.2 (103.69%) | 76944.34 (39.46%) |
| Low-middle SDI | Female | Prevalence | 923812.33 (179.11%) | 97050.92 (18.82%) | 582226.48 (112.88%) | 244534.93 (47.41%) |
| Low-middle SDI | Both | Deaths | 558.27 (74.78%) | 157.18 (21.05%) | 650.06 (87.08%) | -248.98 (-33.35%) |
| Low-middle SDI | Male | Deaths | 148.17 (62.43%) | 36.3 (15.29%) | 195.39 (82.33%) | -83.52 (-35.19%) |
| Low-middle SDI | Female | Deaths | 410.1 (80.54%) | 135.29 (26.57%) | 460.52 (90.44%) | -185.7 (-36.47%) |
| Low-middle SDI | Both | DALYs | 184069.9 (150.23%) | 20186.04 (16.48%) | 127669.03 (104.2%) | 36214.83 (29.56%) |
| Low-middle SDI | Male | DALYs | 47210.9 (133.52%) | 4765.34 (13.48%) | 34795.49 (98.4%) | 7650.07 (21.64%) |
| Low-middle SDI | Female | DALYs | 136858.99 (157.01%) | 16874.4 (19.36%) | 93987.15 (107.83%) | 25997.45 (29.83%) |
| Low SDI | Both | Incidence | 26764.46 (185.89%) | -438.57 (-3.05%) | 22337.91 (155.14%) | 4865.11 (33.79%) |
| Low SDI | Male | Incidence | 8433.98 (171.36%) | -222.78 (-4.53%) | 7375.92 (149.86%) | 1280.84 (26.02%) |
| Low SDI | Female | Incidence | 18330.47 (193.44%) | -158.75 (-1.68%) | 15002.69 (158.32%) | 3486.54 (36.79%) |
| Low SDI | Both | Prevalence | 357963.47 (182.92%) | -8111.8 (-4.15%) | 301905.54 (154.27%) | 64169.73 (32.79%) |
| Low SDI | Male | Prevalence | 107945.12 (168.82%) | -4583.67 (-7.17%) | 95375.27 (149.16%) | 17153.53 (26.83%) |
| Low SDI | Female | Prevalence | 250018.35 (189.76%) | -2251.1 (-1.71%) | 207142.07 (157.21%) | 45127.37 (34.25%) |
| Low SDI | Both | Deaths | 98.42 (69.87%) | -17.86 (-12.68%) | 169.7 (120.48%) | -53.43 (-37.93%) |
| Low SDI | Male | Deaths | 16.71 (46.37%) | -6.32 (-17.53%) | 40.48 (112.34%) | -17.45 (-48.44%) |
| Low SDI | Female | Deaths | 81.71 (77.95%) | -8.12 (-7.75%) | 129.87 (123.9%) | -40.04 (-38.2%) |
| Low SDI | Both | DALYs | 52400.97 (166.19%) | -1550.69 (-4.92%) | 47066.52 (149.27%) | 6885.14 (21.84%) |
| Low SDI | Male | DALYs | 15611.65 (154.35%) | -779.51 (-7.71%) | 14647.18 (144.81%) | 1743.98 (17.24%) |
| Low SDI | Female | DALYs | 36789.32 (171.78%) | -512.57 (-2.39%) | 32523.13 (151.86%) | 4778.76 (22.31%) |
