# Supplementary material for: Global burden and regional disparities of rheumatoid arthritis among the working-age population: A comprehensive analysis from 1990 to 2021 with projections to 2040
Source: PLoS One. 2025 Jun 4;20(6):e0325127. doi: 10.1371/journal.pone.0325127 (PMC12136291; doi:10.1371/journal.pone.0325127)
Supplement: Attachment — Submitted filename: Response to Reviewers.docx [file pone.0325127.s023.docx]

Dear Editor,

Thank you for your letter and insightful advice on our manuscript entitled “Global burden and cross-country inequalities of rheumatoid arthritis among the working-age population: a comprehensive analysis from 1990 to 2021 with projections to 2040”. Accordingly, we have revised the manuscript. All amendments are highlighted in red in the revised manuscript. In addition, point-by-point responses to the comments are listed below this letter.

We hope that the revision is acceptable for publication in your journal.

Yours sincerely,

The Authors

April 7, 2025

**# Journal Requirements:**

1. When submitting your revision, we need you to address these additional requirements.

Please ensure that your manuscript meets PLOS ONE's style requirements, including those for file naming. The *PLOS ONE* style templates can be found at https://journals.plos.org/plosone/s/file?id=wjVg/PLOSOne_formatting_sample_main_body.pdf and https://journals.plos.org/plosone/s/file?id=ba62/PLOSOne_formatting_sample_title_authors_affiliations.pdf

Response: Thank you for this important reminder regarding PLOS ONE's style requirements. We acknowledge this feedback and want to assure you that we have carefully reviewed the PLOS ONE style guidelines and have made the necessary revisions to our manuscript to ensure it fully complies with these requirements. Thank you for bringing this to our attention, and we believe these adjustments will facilitate the smooth processing of our manuscript.

2. Thank you for stating the following in the Acknowledgments Section of your manuscript: [We sincerely appreciate the IHME institution for providing the GBD data. We also thanks to JD_GBDR (V2.34.2, Jingding Medical Technology Co., Ltd.) for his help in our exploration of GBD database.]

Response: This acknowledges and appreciates the contributions of both the IHME institution and JD_GBDR in providing the data and tools that were essential for this research. We hope this is satisfactory.

We note that you have provided funding information that is not currently declared in your Funding Statement. However, funding information should not appear in the Acknowledgments section or other areas of your manuscript. We will only publish funding information present in the Funding Statement section of the online submission form.

Please remove any funding-related text from the manuscript and let us know how you would like to update your Funding Statement. Currently, your Funding Statement reads as follows: “The authors received no specific funding for this work.” Please include your amended statements within your cover letter; we will change the online submission form on your behalf.

Response: Thank you for pointing out the discrepancy regarding our funding information. We understand that funding details should only be included in the Funding Statement section of the online submission form and not within the manuscript itself, including the Acknowledgments section. We apologize for this oversight. We have now removed all funding-related text from the manuscript, including from the Acknowledgments section. Regarding the funding statement, the current statement, "The authors received no specific funding for this work," is accurate. Therefore, we do not have any amended statements to include. We would like the funding statement to remain as “The authors received no specific funding for this work.”

We will ensure that this information is clearly stated in our cover letter as you requested. Thank you for bringing this to our attention and for your assistance in updating the online submission form accordingly.

3. PLOS requires an ORCID iD for the corresponding author in Editorial Manager on papers submitted after December 6th, 2016. Please ensure that you have an ORCID iD and that it is validated in Editorial Manager. To do this, go to ‘Update my Information’ (in the upper left-hand corner of the main menu), and click on the Fetch/Validate link next to the ORCID field. This will take you to the ORCID site and allow you to create a new iD or authenticate a pre-existing iD in Editorial Manager.

Response: Thank you for this reminder. We confirm that the corresponding author has an ORCID iD, and we have already validated it in Editorial Manager as required. The corresponding author's ORCID iD is now correctly linked and validated within the Editorial Manager system.

4. We note that Figure 2 and Supplementary figure 4.5.6 and 7 in your submission contain [map/satellite] images which may be copyrighted. All PLOS content is published under the Creative Commons Attribution License (CC BY 4.0), which means that the manuscript, images, and Supporting Information files will be freely available online, and any third party is permitted to access, download, copy, distribute, and use these materials in any way, even commercially, with proper attribution. For these reasons, we cannot publish previously copyrighted maps or satellite images created using proprietary data, such as Google software (Google Maps, Street View, and Earth). For more information, see our copyright guidelines: http://journals.plos.org/plosone/s/licenses-and-copyright.

We require you to either (1) present written permission from the copyright holder to publish these figures specifically under the CC BY 4.0 license, or (2) remove the figures from your submission.

Response: Thank you for bringing the potential copyright issue regarding the map images in Figure 2 and Supplementary Figures 4, 5, 6, and 7 to our attention. We understand the importance of adhering to the Creative Commons Attribution License (CC BY 4.0) and the journal's copyright policies regarding proprietary map data. To ensure full compliance, we have followed option (2) as suggested and have removed Figure 2 and Supplementary Figures 4, 5, 6, and 7 from our manuscript submission. We appreciate your guidance in ensuring our manuscript meets the journal's publication standards.

5. Please include captions for your Supporting Information files at the end of your manuscript, and update any in-text citations to match accordingly. Please see our Supporting Information guidelines for more information: http://journals.plos.org/plosone/s/supporting-information.

Response: Thank you for providing these clear instructions regarding the formatting of supporting information. We appreciate the guidance and the link to the specific guidelines. In accordance with your request, we have taken the following actions in our revised manuscript: We have compiled a complete list of captions for all supporting information files associated with our submission. This list of captions has been placed at the very end of the main manuscript text. We have carefully reviewed the entire manuscript and updated all in-text citations (e.g., "S1 Table," "S1 Figure") to ensure they accurately correspond to the final Supporting Information file designations and numbering as presented in the caption list. We believe these changes fully address the journal's requirements for supporting information presentation. Thank you again for pointing this out.

**# Reviewer's comments**

The manuscript titled “Global burden and cross-country inequalities of rheumatoid arthritis among the working-age population: a comprehensive analysis from 1990 to 2021 with projections to 2040” presents a valuable epidemiological analysis of rheumatoid arthritis (RA). The study utilizes data from the Global Burden of Disease (GBD) 2021 to examine temporal trends, regional disparities, and future projections. The research is relevant and well-structured, yet there are key areas requiring improvement, particularly in methodological transparency, result interpretation, and language clarity.

Response: We appreciate your time and insightful feedback on our manuscript titled “Global burden and cross-country inequalities of rheumatoid arthritis among the working-age population: a comprehensive analysis from 1990 to 2021 with projections to 2040.” We acknowledge your positive assessment of the study's value and structure, which is encouraging. We fully recognize the critical areas for improvement you identified, particularly concerning methodological transparency, result interpretation, and language clarity, and we concur that addressing these points will significantly enhance the quality of our manuscript. We are now prepared to address each specific comment and suggestion you provided in a detailed and constructive manner. We eagerly await the subsequent specific feedback and are committed to revising our manuscript accordingly to meet the high standards of *PLoS One*. All amendments are highlighted in red in the revised manuscript.

**Specific Comments by Section**
**Title & Abstract**

• Title: The title accurately represents the study, but "cross-country inequalities" could be more precise (e.g., "regional disparities in rheumatoid arthritis burden").

Response: Thank you for your feedback on the title. We agree that "cross-country inequalities" could be more precise. We will revise the title to "Global burden and regional disparities of rheumatoid arthritis among the working-age population: a comprehensive analysis from 1990 to 2021 with projections to 2040" to better reflect the focus of our study. Please refer to lines 1−3.

**Abstract:**

• Line 11: "Objective To evaluates" → "Objective: To evaluate".

Response: Thank you for pointing out this error. We will correct line 11 to read "Objective: To evaluate". We will also carefully proofread the entire manuscript to ensure the elimination of any similar errors. Please refer to line 11.

• Line 19: "the global burden of RA among the working-age population with 11,878,843.81 cases" → The wording is unclear. Rephrase to "In 2021, there were 11.88 million cases of RA in the working-age population globally."

Response: Thank you for highlighting the lack of clarity in this sentence. We agree with your suggested rephrasing and will revise line 19 to "In 2021, there were 11.88 million cases of RA in the working-age population globally." This more clearly and concisely presents the information. Please refer to lines 19−20.

• Line 25: "Regionally, high SDI regions exhibited the highest ASPR, ASIR, and age-standardized DALY rates, while middle SDI regions had the highest ASDR rate." → Clarify why middle SDI regions show the highest ASDR despite high SDI regions having the highest burden.

Response: Thank you for raising this important point regarding the seemingly counterintuitive finding in line 25. We recognize that the current phrasing lacks the necessary nuance to explain this observation. We rewrote this sentence and further elaborated on this potential explanation in the *Discussion* sections by referencing existing literature on healthcare access and quality across different SDI regions. Please refer to lines 25−29 and 398−407.

• Conclusion (Lines 31-33): The conclusion should better reflect the study’s policy implications, such as specific interventions.

Response: Thank you for reminding us of the current conclusion in the abstract. We appreciate the opportunity to further refine it based on your previous feedback regarding policy implications. We acknowledge that while the current conclusion mentions "targeted interventions," it lacks specificity regarding the nature of these interventions based on our findings. To address this more directly, we have made the revision to the conclusion. Please refer to lines 33−38.

**Introduction**
• Lines 36-41: The introduction effectively introduces the problem but could be more concise.

Response: Thank you for the suggestion to improve the conciseness of the introduction. We agree that the current phrasing in lines 36-41 could be more streamlined. We propose the following revised version: "Rheumatoid arthritis (RA) is a prevalent autoimmune disorder causing chronic, symmetrical joint inflammation, leading to cartilage and bone damage and impaired joint function [1]. Beyond joints, RA can cause systemic complications like cardiovascular, pulmonary, and mental health disorders, significantly impacting quality of life [2]."

We believe these changes make the introduction more concise while retaining the essential information about RA. We will apply this revision to the manuscript. Please refer to lines 42−46.

• Lines 42-51: The economic implications of RA are well stated, but referencing more recent economic studies would strengthen the argument.

Response: Thank you for the clarification. In response to the suggestion to include more recent economic studies in lines 42-51, we would like to clarify that we have already updated the cited references to include the most recent and relevant literature available on the economic implications of rheumatoid arthritis. These updated references encompass recent findings on the cost of illness, impact on work productivity, and broader societal economic burden associated with RA. Please refer to lines 50−55.

• Lines 52-66: The gap in research is well articulated, but a stronger transition into the study objectives is needed.

Response: Thank you for pointing out the need for a stronger transition into the study objectives. We acknowledge this suggestion. While the subsequent paragraph does explicitly state the study objectives, we agree that a more direct linkage to the research gaps outlined in lines 52-66 would enhance the clarity and flow of the introduction. To address this, we have slightly modified the last few sentences of the objectives paragraph to more directly reference the gaps. Please refer to lines 66−71.

**Methods**

Data Source (Lines 79-95):

• The use of GBD 2021 data is appropriate, but the authors should explicitly state whether any adjustments were made to the dataset or how missing data were handled.

Response: Thank you for raising this important point regarding the data source. We will explicitly state in the *Methods* section that we utilized the official, publicly available GBD 2021 results database as provided by the Institute for Health Metrics and Evaluation. To address the query about adjustments and missing data, we will clarify the following: "The dataset utilized in this research was accessed through the Global Health Data Exchange query tool, available at https://vizhub.healthdata.org/gbd-results/. As we utilized the final, processed estimates provided within the GBD 2021 results tool, no further adjustments or handling of missing data were performed by the authors." Please refer to lines 98−101.

• The authors mention DisMod-MR 2.1 (Line 84) but do not describe potential limitations of this model.

Response: Thank you for highlighting this point regarding the discussion of DisMod-MR 2.1 limitations. We acknowledge that while we mention the use of DisMod-MR 2.1 in the *Methods* section (line 84), we did not elaborate on its potential limitations within that specific section. However, we would like to clarify that a detailed discussion of the inherent limitations associated with the GBD methodology, including the DisMod-MR 2.1 model, is provided as the first point in the dedicated *Limitations* section of our manuscript. Please refer to lines 471−476.

Study Population (Lines 108-118):

• The age stratification is appropriate, but no justification is given for excluding those older than 64 years.

Response: Thank you for raising this question regarding the age range used in our analysis. We appreciate the opportunity to clarify our rationale. The primary focus of our study, as reflected in the title and objectives, is the burden of rheumatoid arthritis specifically within the working-age population. We defined this demographic group using the commonly accepted age range of 15 to 64 years.

We aimed to provide a detailed analysis of RA's impact on individuals during their most economically active years, a period where the disease can significantly affect work capacity, lead to early retirement, and impose substantial economic burdens. Therefore, our age stratification was deliberately chosen to align with this specific research focus.

However, we acknowledge that RA also poses a significant burden on individuals older than 64. If you feel that extending the age range to include older populations would substantially enhance the manuscript's scope and relevance, we are certainly willing to consider performing and incorporating this additional analysis.

Statistical Analysis (Lines 130-182):

• The EAPC metric is well explained, but the authors should discuss its limitations in estimating trends over long periods.

Response: Thank you for highlighting this important aspect of the EAPC metric. We acknowledge that while EAPC provides a useful summary measure of the average trend over a period, it does have limitations, particularly when applied over extended timeframes like the 32 years covered in our study (1990−2021).

The primary limitation, as you correctly imply, is that EAPC assumes a constant proportional change (log-linear trend) over the entire period. This single average rate can potentially mask significant fluctuations, changes in direction, or periods of acceleration/deceleration in the trend that may have occurred within the 32-year timeframe. Factors such as advancements in RA treatment, changes in diagnostic criteria, or shifts in population risk factors could lead to non-linear trends that are not fully captured by a single EAPC value.

We recognize the importance of acknowledging this methodological constraint. Therefore, we have included a discussion of this specific limitation of the EAPC metric in the *Limitations* section of our manuscript. Please refer to lines 481−484.

• The decomposition analysis needs more methodological details—specifically, how confounding factors were controlled.

Response: Thank you for requesting further clarification on the Das Gupta decomposition analysis, specifically regarding the control of confounding factors. We appreciate the opportunity to elaborate on this aspect of our methodology.

You are correct that we employed the Das Gupta decomposition method. This method is a standard demographic technique used to partition the difference in an overall rate or total number (e.g., total prevalent cases) between two time points (1990 and 2021 in our study) into components attributable to changes in underlying factors. In our application, we decomposed the change in the total number of RA prevalent cases, incident cases, deaths, and DALYs among the working-age population (15−64-year-old) into three specific components:

(1) Population Aging: The contribution due to changes in the age structure within the 15−64-year-old age group (e.g., a shift towards older ages within this range).

(2) Population Growth: The contribution due to the change in the overall size of the 15−64-year-old population.

(3) Epidemiological Change: The contribution due to changes in the age-specific rates of RA prevalence, incidence, mortality, or DALYs, reflecting changes independent of demographic shifts.

Regarding the crucial point about controlling for confounding factors, it is important to understand the nature of the Das Gupta decomposition. This method is primarily a mathematical attribution technique, not a statistical model designed to adjust for confounding variables in the way that, for example, multivariate regression analysis does. The Das Gupta method takes the observed (or, in our case, GBD-estimated) age-specific population counts and age-specific RA rates for 1990 and 2021 as inputs. It then calculates how much of the total change in the RA burden is mathematically attributable to changes in population size, age structure, and the rates themselves.

Therefore, the Das Gupta decomposition itself does not directly control for confounding factors like changes in diagnostic criteria, treatment efficacy, healthcare access, or underlying risk factor prevalence over the 32-year period. These factors would influence the epidemiological change component (i.e., the changes in age-specific RA rates).

However, the input data for our decomposition—the age-specific estimates of RA prevalence, incidence, mortality, and DALYs—are derived from the GBD 2021 study. The GBD methodology employs sophisticated modeling strategies to generate these estimates. This process aims to synthesize all available data, adjust for methodological differences between studies, and account for known drivers of disease burden to produce the most reliable and comparable estimates possible. To the extent that the GBD modeling successfully accounts for temporal trends and adjusts for certain factors, some level of confounding control is inherent in the input data we used for the decomposition.

In summary, the Das Gupta analysis attributes observed changes to demographic vs. epidemiological factors based on GBD estimates; it doesn't independently control for confounders during the decomposition calculation. The control for confounding relies on the robustness of the underlying GBD 2021 estimation process that generated the age-specific rates used in our analysis.

**Results**

Lines 185-238 (Global and Regional Burden):

• Data presentation is strong, but some statistics appear redundant.

Response: Thank you for your remark regarding the presentation of data in the *Results* section (Lines 185-238). In reviewing this section, we recognized that while aiming for comprehensiveness, we might have included certain statistics in the text that could be considered redundant given their presence in the detailed tables and figures. Therefore, we have already revised this section to enhance its conciseness and reduce redundancy. Please refer to lines 190−236.

• The statement that "RA burden remains concentrated in high SDI countries" (Line 228) should be more nuanced—low SDI countries may have underreporting issues.

Response: Thank you for raising this important point regarding the interpretation of RA burden across different SDI regions. We strongly agree that the statement "RA burden remains concentrated in high SDI countries" requires careful contextualization due to potential data limitations in lower SDI settings.

We recognized this critical issue during our analysis and interpretation. Therefore, we have explicitly addressed this limitation in the *Limitations* section of our manuscript. We explicitly caution readers that comparisons of RA burden across countries and SDI regions should be interpreted with these potential data limitations and reporting biases in mind. Please refer to lines 476−479.

Lines 253-271 (National Burden):

• The interpretation of the burden in countries like Equatorial Guinea needs further clarification—why does it have the fastest increase in ASPR? Possible factors should be discussed.

Response: Thank you for pointing out the need for further clarification regarding the rapid increase in the ASPR of rheumatoid arthritis observed in Equatorial Guinea. We agree that such a specific and pronounced finding warrants a discussion of potential contributing factors beyond simply reporting the statistic. In response to your comment, we have now incorporated possible factors into the manuscript's *Discussion* section, exploring potential reasons for this observation. Please refer to lines 408−417.

**Figures & Tables:**

• The figures are clear but lack explicit references in some parts of the text.

Response: Thank you for pointing out the instances where explicit figure references were missing in the text. We agree that consistently linking the narrative descriptions to the corresponding figures is crucial for clarity and reader navigation. To address this, we have thoroughly reviewed the manuscript text.

• The tables should be formatted to ensure consistency in decimal places (e.g., some values are reported with two decimals, others with three).

Response: Thank you for meticulously reviewing our manuscript and pointing out the inconsistency in decimal places within the tables. We agree that maintaining uniformity in data presentation is essential for clarity and professionalism. In response to your comment, we have carefully reviewed all tables and have standardized the number of decimal places (S5 Table). We believe this revision enhances the readability and professional presentation of our results and addresses your concern effectively.

**Discussion**

• Lines 368-396: The discussion is comprehensive but contains excessive restatement of results.

Response: Thank you for this feedback on the discussion section (lines 368-396). We acknowledge that there might have been some redundancy in restating the results within the discussion. In response to this point, we confirm that we have carefully reviewed lines 368-396 and have made revisions to address the excessive restatement of results. Please refer to lines 363−383.

• Lines 397-414: The comparison to previous studies is useful, but more direct citations of GBD 2021 findings would strengthen credibility.

Response: Thank you for your feedback regarding the comparison to previous studies (lines 397-414) and the suggestion to include more direct citations of GBD 2021 findings. We appreciate the point about strengthening credibility through direct citations. We would like to clarify that we have cited the GBD 2021 Rheumatoid Arthritis Collaborators study, which serves as the primary source for the GBD 2021 data on rheumatoid arthritis. Furthermore, as there are currently limited published studies that specifically analyze the GBD 2021 data of RA, we have also referenced other relevant and available studies that address aspects of RA burden, trends, and disparities. This allows us to contextualize our findings within the broader existing literature.

• Lines 415-443: The section on gender differences is well-developed, though the discussion of X-chromosome-related immunity (Lines 417-421) should cite more recent studies.

Response: Thank you for your feedback on the section addressing gender differences, and for specifically pointing out the need for more recent citations regarding X-chromosome-related immunity.

We acknowledge this suggestion and confirm that we have now updated the citations in Lines 417-421 to include more recent and relevant studies that support the discussion on the role of X-chromosome-related immunity in the observed gender differences in rheumatoid arthritis. Please refer to lines 418−426.

• Lines 444-462 (Risk Factors): The discussion on smoking and dietary habits is relevant, but more clarity on whether these factors explain observed regional variations is needed.

Response: Thank you for raising this important point. We acknowledge that the current text in Lines 444-462 does not explicitly address whether these factors explain the higher burden in high-income regions.

Our understanding is that while smoking is a known risk factor for RA, its prevalence and impact might vary across regions. Similarly, dietary patterns differ significantly worldwide. However, establishing a direct causal link between these specific risk factors and the higher burden in high SDI regions requires careful consideration and, ideally, region-specific data on the prevalence of these risk factors and their attributable risk for RA. Further research is needed to investigate the prevalence of risk factors across different SDI regions and their contribution to the observed disparities. We revise the discussion to reflect this nuance. Please refer to lines 459−465.

**Limitations**

Lines 464-477: The limitations are well acknowledged, but additional concerns should be mentioned:

• 1. Potential biases due to underreporting in lower-income countries.

Response: Thank you for highlighting this crucial limitation. We acknowledge the significant potential for bias due to underreporting of rheumatoid arthritis cases in lower-income countries. This is a critical factor that could affect the accuracy of the estimated global burden and the observed regional disparities. We confirm that we have now explicitly added this concern to the *Limitations* section of our manuscript. Please refer to lines 476−479.

• 2. The reliance on DisMod-MR 2.1 for estimates without validation using independent datasets.

Response: Thank you for pointing out the limitation regarding the reliance on DisMod-MR 2.1 estimates. We appreciate the opportunity to clarify how we have addressed this in our manuscript. As you correctly stated, the first limitation we have outlined already encompasses the concern about the DisMod-MR 2.1 model.

This statement directly addresses the reliance on the model and acknowledges that the estimates are based on limited primary data in many countries. The model itself is a standardized tool used by the GBD for generating estimates in the absence of complete data, and as researchers using GBD data, we can't independently validate or modify the model's outputs. Our limitation statement reflects this reality and highlights that the accuracy of the GBD estimates, and consequently our findings, is inherently tied to the robustness and input data of the DisMod-MR 2.1 model.

We believe this adequately acknowledges the limitation associated with the use of model-based estimates without independent validation using separate datasets, which is a common challenge when working with comprehensive global burden of disease data. Please refer to lines 471−476.

**Conclusion**

• Lines 478-492: The conclusion effectively summarizes the study but should highlight specific policy recommendations.

Response: Thank you for your feedback regarding the *Conclusion* section. In response to your suggestion, we have rewritten the conclusion. We believe this revised conclusion now more effectively highlights the practical implications of our study and directly addresses your valuable comments. Please refer to lines 490−505.

• Suggest incorporating a final sentence on the necessity for improved healthcare access in low- and middle-SDI regions.

Response: Thank you for your feedback regarding the *Conclusion* section. In response to your suggestion, we have rewritten the conclusion. We believe this revised conclusion now more effectively highlights the practical implications of our study and directly addresses your valuable comments. Please refer to lines 490−505.

**Language & Clarity**

There are multiple grammatical and syntactical errors throughout the manuscript. Examples:

• Line 10: “To evaluates” → “To evaluate”.

Response: Thank you for pointing out this error. We will correct line 11 to read "Objective: To evaluate". We will also carefully proofread the entire manuscript to ensure the elimination of any similar errors. Please refer to line 11.

• Line 19: “the global burden of RA among the working-age population with 11,878,843.81 cases” → unclear wording.

Response: Thank you for highlighting the lack of clarity in this sentence. We agree with your suggested rephrasing and will revise line 19 to: "In 2021, there were 11.88 million cases of RA in the working-age population globally." This more clearly and concisely presents the information. Please refer to lines 19−20.

• Line 121: “It serves to capture the social and economic conditions that influence health outcomes in specific locations” → awkward phrasing.

Response: Thank you for identifying the awkward phrasing in Line 121. In response to your comment, we confirm that we have removed this specific description from the manuscript. We believe this deletion improves the overall conciseness and flow of the text in this section.
