# Supplementary material for: Global burden and regional disparities of rheumatoid arthritis among the working-age population: A comprehensive analysis from 1990 to 2021 with projections to 2040
Source: PLoS One. 2025 Jun 4;20(6):e0325127. doi: 10.1371/journal.pone.0325127 (PMC12136291; doi:10.1371/journal.pone.0325127)
Supplement: Attachment — Submitted filename: Response_to_Reviewers_auresp_2.docx [file pone.0325127.s024.docx]

Dear Editor,

Thank you for your letter and insightful advice on our manuscript entitled “Global burden and regional disparities of rheumatoid arthritis among the working-age population: a comprehensive analysis from 1990 to 2021 with projections to 2040”. Accordingly, we have revised the manuscript. All amendments are highlighted in red in the revised manuscript. In addition, point-by-point responses to the comments are listed below this letter.

We hope that the revision is acceptable for publication in your journal.

Yours sincerely,

The Authors

May 6^th^, 2025

**# Journal Requirements:**

Please review your reference list to ensure that it is complete and correct. If you have cited papers that have been retracted, please include the rationale for doing so in the manuscript text, or remove these references and replace them with relevant current references. Any changes to the reference list should be mentioned in the rebuttal letter that accompanies your revised manuscript. If you need to cite a retracted article, indicate the article's retracted status in the References list and also include a citation and full reference for the retraction notice.

Response: Thank you for this important instruction. We have thoroughly reviewed our reference list to ensure its completeness and correctness. We confirm that, to the best of our knowledge, none of the references cited in our manuscript have been retracted. Therefore, no changes have been made to the reference list at this time.

**# Reviewer's comments**

**Reviewer #1**

(No Response)

Response: We appreciate your time and effort in reviewing our manuscript. Your acknowledgment encourages us, and we believe this suggests that the manuscript is well-received overall. We remain open to any further feedback or suggestions you might have as we move forward in the publication process. Thank you again for your valuable consideration.

**Reviewer #2**

Lines 237-239: to please check Eastern Europe.
Response: Thank you for pointing out this error regarding the ASDR in Eastern Europe and Southern Latin America. Upon careful review of our data, we have confirmed that the ASDR in Eastern Europe was indeed higher than that in Southern Latin America. This was an oversight on our part, and we have already corrected this statement in lines 237-239 of the revised manuscript to accurately reflect our findings. We apologize for this inaccuracy and appreciate you bringing it to our attention, as it has allowed us to improve the precision of our results reporting.

Line 247: Please check Central Asia, which has a higher rate than the region mentioned in the text.
Response: Thank you for your meticulous review and for identifying this error concerning the EAPC of the age-standardized DALYs rate in Central Asia and Andean Latin America. Upon re-examination of our data, we have confirmed that the EAPC for Central Asia was indeed higher than that for Andean Latin America. This was an inaccuracy in our initial reporting, and we have now corrected this statement in lines 246-247 of the revised manuscript to accurately reflect these values. We appreciate you drawing our attention to this detail, as ensuring the precision of our results is paramount.

Figures to be labeled adequately (similar to the tables).
Response: Thank you for this important feedback regarding the labeling of our figures. We agree that clear and comprehensive labeling is essential for the proper interpretation of visual data. We will thoroughly review all figures in our manuscript and ensure that they are adequately labeled with clear axis labels and legends that provide sufficient information to understand the content of each figure independently. We will strive for consistency in labeling style between figures and tables to enhance the overall clarity and professionalism of our manuscript.

Lines 291-301: Consider adding, even though in females there is gradual increase in the incidence and the prevalence of RA, the DALYs interestingly show decrementing rates from 50 years of age and onwards.

Response: Thank you for this insightful suggestion regarding the trends in DALYs among females aged 50 years and older. We agree that the observation of decreasing DALY rates in this group, despite increasing incidence and prevalence, is an important point to highlight. We have now incorporated this observation into our manuscript in lines 297-299, as suggested. This addition helps to provide a more nuanced interpretation of the burden of rheumatoid arthritis in females across different age groups. We appreciate your contribution in identifying this noteworthy trend.
